# Prevention and Treatment of Chemotherapy-Induced Peripheral Neuropathy (CIPN) with Non-Pharmacological Interventions: Clinical Recommendations from a Systematic Scoping Review and an Expert Consensus Process

**DOI:** 10.3390/medsci11010015

**Published:** 2023-01-30

**Authors:** Nadja Klafke, Jasmin Bossert, Birgit Kröger, Petra Neuberger, Ute Heyder, Monika Layer, Marcela Winkler, Christel Idler, Elke Kaschdailewitsch, Rolf Heine, Heike John, Tatjana Zielke, Beeke Schmeling, Sosamma Joy, Isabel Mertens, Burcu Babadag-Savas, Sara Kohler, Cornelia Mahler, Claudia M. Witt, Diana Steinmann, Petra Voiss, Regina Stolz

**Affiliations:** 1 Department of General Practice and Health Services Research, University Hospital Heidelberg, 69120 Heidelberg, Germany; 2 Institute for General Practice and Interprofessional Care, University Hospital Tübingen, 72076 Tübingen, Germany; 3 National Center for Tumor Diseases, University Hospital Heidelberg, 69120 Heidelberg, Germany; 4 Women’s Clinic, Community Hospital Karlsruhe, 76133 Karlsruhe, Germany; 5 Center for Integrative Medicine, Cantonal Hospital St. Gallen, 9007 St. Gallen, Switzerland; 6 Department of Naturopathy and Integrative Medicine, Robert-Bosch-Krankenhaus, 70376 Stuttgart, Germany; 7 Center for Integrative Oncology, Die Filderklinik, 70794 Filderstadt-Bonlanden, Germany; 8 Anthroposophic Nursing Network in Germany, Academy for Nursing Professions at the Filderklinik, Die Filderklinik, 70794 Filderstadt-Bonlanden, Germany; 9 Clinic for Radiation Therapy and Special Oncology, Hannover Medical School, 30625 Hannover, Germany; 10 Department of Internal and Integrative Medicine, Evang. Kliniken Essen-Mitte, Faculty of Medicine, University of Duisburg-Essen, 45136 Essen, Germany; 11 Department of Health, Zurich University of Applied Sciences, 8401 Winterthur, Switzerland; 12 Department of Nursing Science, Institute of Health Sciences, University Hospital Tübingen, 72076 Tübingen, Germany; 13 Institute for Complementary and Integrative Medicine, University Hospital Zürich and University of Zürich, 8091 Zürich, Switzerland

**Keywords:** chemotherapy-induced peripheral neuropathy (CIPN), complementary therapies, supportive therapy, scoping review, consensus process, cancer, integrative oncology, interprofessional healthcare, non-pharmacological interventions

## Abstract

**Background:** Most individuals affected by cancer who are treated with certain chemotherapies suffer of CIPN. Therefore, there is a high patient and provider interest in complementary non-pharmacological therapies, but its evidence base has not yet been clearly pointed out in the context of CIPN. **Methods:** The results of a scoping review overviewing the published clinical evidence on the application of complementary therapies for improving the complex CIPN symptomatology are synthesized with the recommendations of an expert consensus process aiming to draw attention to supportive strategies for CIPN. The scoping review, registered at PROSPERO 2020 (CRD 42020165851), followed the PRISMA-ScR and JBI guidelines. Relevant studies published in Pubmed/MEDLINE, PsycINFO, PEDro, Cochrane CENTRAL, and CINAHL between 2000 and 2021 were included. CASP was used to evaluate the methodologic quality of the studies. **Results:** Seventy-five studies with mixed study quality met the inclusion criteria. Manipulative therapies (including massage, reflexology, therapeutic touch), rhythmical embrocations, movement and mind–body therapies, acupuncture/acupressure, and TENS/Scrambler therapy were the most frequently analyzed in research and may be effective treatment options for CIPN. The expert panel approved 17 supportive interventions, most of them were phytotherapeutic interventions including external applications and cryotherapy, hydrotherapy, and tactile stimulation. More than two-thirds of the consented interventions were rated with moderate to high perceived clinical effectiveness in therapeutic use. **Conclusions:** The evidence of both the review and the expert panel supports a variety of complementary procedures regarding the supportive treatment of CIPN; however, the application on patients should be individually weighed in each case. Based on this meta-synthesis, interprofessional healthcare teams may open up a dialogue with patients interested in non-pharmacological treatment options to tailor complementary counselling and treatments to their needs.

## 1. Introduction

Chemotherapy-induced peripheral neuropathy (CIPN) develops due to neurotoxic treatments, in particular taxanes, vinca alkaloids, platinum agent, proteasome inhibitors, and thalidomide [1]. Such treatments attack the cellular and sub cellular level and cause altered ion channel activity (sodium, potassium, calcium) as well as changes in the intracellular systems, which are responsible for oxidative stress, neuroinflammation, and mitochondrial dysfunction [2,3]. In contrast to nociceptive pain, which occurs when a painful stimulus activates the peripheral nociceptors, neuropathic pain is not the result of damaged tissue, but is caused by inner structural deficits in the peripheral neurons and sensory nerves [2,3]. Unpleasant CIPN symptoms appear in a variety of ways. Patients often experience numbness in their feet and palms as well as paresthesia, acroataxia, and the loss of motor functions, which contribute to the fact that patients with CIPN have a high risk for falling injuries [4]. For many patients, even opening a water bottle is painful or they feel like “walking on glass” [4] (a statement of some experts participating in the symposium, see phase 2 under 2.2 “Structured expert consensus process”). Up to 71% of patients undergoing acute treatment (e.g., Oxaliplatin, Docetaxel) experience CIPN [5], and it is one of the major symptoms that affects why patients may decide to cease their treatment. Patients who develop CIPN are both at younger and older age, experience restrictions in their quality-of-life functions, and therefore need effective treatment options [6]. Up to 42% of patients experience CIPN two years after they started their taxane- and platinum-based chemotherapy [7], and they are also in need for effective strategies for improving their symptoms and increasing their quality of life [8]. 

Currently, there are not enough conventional CIPN treatments available, and the most prescribed medication is Duloxetine, even though its effect size has been reported to be moderate [9,10]. With the help of a systematic review, Hou et al. 2018 [1] identified 26 other treatment options in 35 included studies that were also on laser therapy and acupuncture. The results, however, need to be considered with caution, as most studies had small sample sizes and a variety of outcome measures. Hence, there is a need for pointing out further treatment options to alleviate CIPN symptoms in order to relieve the suffering of those affected. This is also shown in the fact that research indicates that up to 80% of patients with cancer have an interest in so-called natural, non-pharmacological, complementary interventions for self-managing their symptoms and actively take control in their own symptom management [11,12]. Surveys have also identified an increase of cancer patients’ use of complementary therapies from the timepoint before to after a cancer diagnosis [13]. In this course of time, for example, the use of biologic products for general symptom management and for coping with the new life situation has tripled to 52% [13]. Thus, healthcare teams should know how to consult patients suffering from CIPN on complementary treatment options when conventional drugs like Duloxetine or Gabapentine [14] are not effective enough or when patients’ preference is oriented towards complementary and integrative health care (CIH).

Previous literature reviews have examined key complementary treatments that can be applied to relieve general cancer pain [15] or neuropathic pain resulting from drug-based tumor therapy [16]. The latter systematic review, however, has not included nursing interventions, like external applications, which are relevant to consider when aiming to relieve patients’ symptoms and treat them in a holistic and natural way. Even though Brami et al. [16] did suggest applying complementary therapies to prevent or treat CIPN—for example, Vitamin E, L-Glutamine, Goshajinkigan, and Omega-3—the authors think that the oral intake of such natural products is not the only option available. In the last years, there have been other studies published reporting that henna applications [17] or cryocompression [18,19] are also beneficial, to name just a few more options, and they can be applied for CIPN symptom management.

Therefore, the purpose of this article is to provide interprofessional healthcare teams with a comprehensive literature review and clinical recommendations of the best available evidence on complementary treatments for the prevention of CIPN and for the supportive management of CIPN during or after conventional treatment. 

## 2. Materials and Methods

The methodology for outlining the clinical recommendations of evidence-based complementary prevention and treatment options followed a two-phase guideline [20,21]. In Phase 1, a scoping review to find external evidence by published studies was conducted, and in Phase 2, a structured expert consensus process on complementary interventions for CIPN was performed. This was followed by a synthetization of both phases in terms of their non-pharmacological treatment options.

According to Sackett’s approach of evidence-based medicine, individual clinical decisions should be made by considering the best available scientific evidence, the personal expertise of the health professionals, and the needs and values of the persons to be treated [22]. Ideally, the highest level of evidence is available for the specific clinical decision question from randomized controlled trials (RCTs), systematic reviews, or meta-analyses. For some questions, however, only evidence from lower levels is available, so that the next possible evidence must be sought and considered [22]. In the hierarchy of evidence, assessments by experts are assigned to the lowest level of evidence [23].

Based on clinical experience with naturopathic medicine, especially in German-speaking hospitals, we experienced that many different symptom management methods are used as supportive care for CIPN. Furthermore, due to the different levels of evidence and standardization of these methods, the authors wanted to first clarify these commonly used methods in a consensus meeting and then compare those results with the systematic literature search. The authors also see that approaches based on consensus and literature reviews make a significant contribution to the prevention and treatment of various symptoms in patients with cancer in the field of nursing and integrative care practice [21,24]. Therefore, the authors think that this study will contribute to the literature, especially in terms of comparing the consensus-based results of the practices used in German-speaking countries by an expert consensus team with the results of a systematic literature search.

### 2.1. Phase 1: Scoping Review

#### 2.1.1. Literature Search

A review of the literature was performed following the *Preferred Reporting Items for Systematic Reviews and Meta-Analyses Extension for Scoping Reviews (PRISMA-ScR) Checklist* (Supplementary S1) [25]. As recommended, the frameworks by Arksey and O’Malley [26] and Levac et al. [27] were used as a guidance, as presented in the synthesis and enhancement of the Joanna Briggs Institute (JBI) [28]. A primary search on CIPN (or the risk of developing CIPN) as the main outcome was performed using the terms “(cancer) AND (neuropathies or peripheral neuropathy or CIPN) AND (complementary therapies or non-pharmacological therapies)”. An additional search was conducted on general pain as the main outcome and also including other chronically ill patients, to get further insights for transferability of the results to CIPN. In line with the definition by the National Center for Complementary and Integrative Health (NCCIH) [29] and by Witt et al. 2017 [30], the authors understand non-pharmacological interventions in the field of integrative oncology to be evidence-based and complementary to conventional therapy, which can help alleviate patients’ symptoms and enhance their quality of life and resilience. A primary search was performed using the terms “(cancer) AND (neuropathies or peripheral neuropathy or CIPN) AND (complementary therapies)”. Search strategies were adapted for the databases of Pubmed/MEDLINE, PsycINFO, PEDro, Cochrane Central, and CINAHL, ending on 31 July 2021. The full search strategy can be found in Supplementary S2 and was reported in the review protocol registered at PROSPERO 2020 (CRD 42020165851) [31].

#### 2.1.2. Study Screening and Selection Criteria

The inclusion criteria were designed to include all possible study types (e.g., RCTs, controlled studies, cohort studies, case studies, pilot studies, expert opinions, reviews, qualitative studies, clinical guidelines). Articles were included in this review if adult patients with cancer (search one) or chronically ill patients were the focus of interest of the research (extended search two). There were no restrictions by population characteristics (e.g., type of cancer, stage of cancer, adults, gender, comorbidities, country, geographic location, ethnicities, cultural groups). Only studies published in English, French, or German language were included. Study protocols and pre-clinical studies were excluded. Studies with children were also excluded as such patients require special pediatric treatment. 

#### 2.1.3. Data Extraction and Reporting

**Figure 1:**
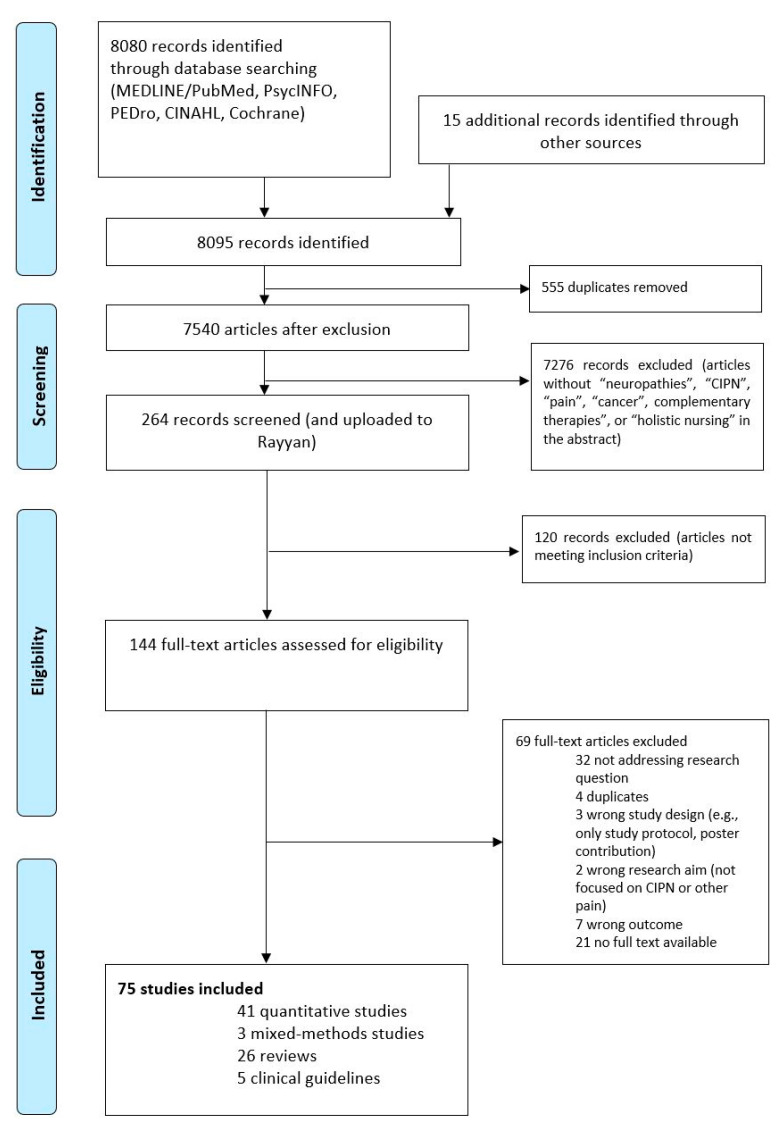
Flowchart of article selection process.

Titles and abstracts were retrieved by two review authors (NK, RS) working independently, and then imported to EndNote X9 © (Clarivate Analytics, PA, USA) and deduplicated. Selected records were then uploaded to Rayyan (Qatar Computing Research Institute, Doha, Qatar) [32] and screened for eligibility. Then, a core team of four researchers (RS, BK, JB, NK) screened titles and abstracts, with relevant articles selected for full-text review. This was conducted in duplicate, so that there was a mutual review and further coordination if necessary. Disagreements on inclusion or exclusion were resolved by regularly discussing and solving the “conflicts” displayed in Rayyan. In Figure 1, the search and screening and selection process of the articles are documented according to PRISMA-ScR. A data charting form was developed to capture relevant data from studies (see Tables S1 and S2 in the Supplementary S3 and S4).

#### 2.1.4. Risk of Bias Assessment

All included studies were assessed using the Critical Appraisal Skills Program (CASP) [33], which consists of a set of criteria to proof, and is available for different study types. For instance, the *CASP Randomized Controlled Trials Checklist* (2017) entails 11 questions regarding the quality of the study (Supplementary S5, Table S3: Critical appraisal of the included studies). Microsoft Excel and FreeHand were used for graphical display, data cleaning, formatting, and analysis. 

### 2.2. Phase 2: Structured Expert Consensus Process

The methodology is based on the systematic and structured process of expert consensus used by the AWMF (Arbeitsgemeinschaft der Wissenschaftlichen Medizinischen Fachgesellschaften e.V. = Association of the Scientific Medical Societies) to develop scientific clinical guidelines [34]. For this second phase, a two-day workshop was planned under the theme “Supportive management for CIPN in patients with cancer” and took place in September 2018 at the Evang. Kliniken Essen-Mitte in Essen, North Rhine-Westphalia, Germany. Sixteen experts with a naturopathic background and experience in cancer treatment and care from nine different clinical settings in Germany and Switzerland discussed non-pharmacological treatment options for CIPN. The expert panel consisted of 11 nurses, 4 physicians, and 1 psychologist. The aim of the symposium was to share experiences and also to develop clinical and research recommendations for the supportive treatment of CIPN. As there is no professional society for naturopathic care in Germany, renowned experts from clinics with many years of experience in naturopathy were invited to the symposium. In the meantime, the expert group has become a working group of the German Society for Nursing Science section of oncological nursing (https://dg-pflegewissenschaft.de/sektionen/klinische-pflege/onkologische-pflegeforschung-2/, accessed on 1 December 2022).

#### Criteria for Consensus Finding

On the first day, all supportive adjunct therapies for CIPN were discussed under the question “What measures do you use to prevent or treat CIPN in clinical practice?” in the expert panel. Since all measures have already been applied in clinical use for many years, only safe measures were mentioned. In total, 17 complementary treatments were summarized on a collecting board. On the second day, all the complementary treatments were further consented. For arranging and classifying the nursing interventions, the following criteria were considered as highly relevant: safety, clinical experience, effort of training, and practical feasibility. It was consented to rate all complementary treatments on these criteria as follows:

- S = safe;
- CE = clinical experience (rated on a numerical scale 0 to 5, with 0 = no effect and 5 = maximum effect);
- ET = effort of training (education requirements in addition to a nursing grade; 0 = no additional instructions or education needed, 1 = instructions needed, 2 = application under guidance, 3 = repeated practice needed, 4 = basic training of rhythmical embrocation (200 h) recommended but partial skills can be acquired with less than 200 h, and 5 = basic training of rhythmical embrocation (200 h) needed);
- PF = practical feasibility (PFt = feasibility limited due to time requirements; PFtt = feasibility strongly limited due to time requirements; PFc = feasibility limited due to high costs (>30 EUR per month)).

The consensus process is based on the AWMF’s systematic and structured process for the development of scientific clinical guidelines [34]. It was moderated by two physicians (DS, PV) and each intervention was evaluated on the above-mentioned criteria. Each clinic had one vote, and when a clinic was represented by more than one participant, the participants had to coordinate their vote with each other.

## 3. Results

### 3.1. Search Results

A total of 264 potentially relevant articles were identified and screened, wherein 145 articles were assessed in detail, of which 75 studies (41 quantitative studies, 3 mixed-methods studies, 26 reviews, 5 clinical guidelines) were included in the scoping review (Figure 1). Most studies were conducted in North America (*n* = 27), Europe (*n* = 18), Asia (*n* = 16), the Middle East (*n* = 8), UK (*n* = 4), and Australia (*n* = 2). 

### 3.2. Consensus from the Expert Panel

During the two-day expert symposium (08/18), consensus was found for 17 non-pharmacological interventions (see Table 1, and detailed version in Table S4 in Supplementary S6), and some could also/only be applied for preventive use. The experts rated all nursing interventions as safe, and practical feasibility was given for 15 of them. Only for the therapeutic application of rhythmical embrocations (RE) practical feasibility was assessed as limited because RE are more time-consuming and require a high level of qualification. All other interventions can be performed by the patients themselves after instruction.

### 3.3. Preventative Options for CIPN

The review included 14 studies focused on CIPN (Table 1) that dealt with preventive interventions. Those were phytotherapy (Phy) (henna application) on hands and feet [17], Phy in Traditional Chinese Medicine [35], and Phy for hand and foot baths or fumigation [36]; sensorimotor training (SM), e.g., been bath [37], physical activity [38], cryotherapy (CT) [19,39,40], massage (M) [41], and cryocompression (CC) [18]; some nutritional interventions [16,42]; and acupuncture/acupressure [42]. Additionally, compression was recommended by one study [43] as well as by the expert panel. This recommended in total six interventions that can be used for prevention, but without an assessment of their effectiveness (Table 1).

### 3.4. Complementary Treatment Options for CIPN

**Figure 2:**
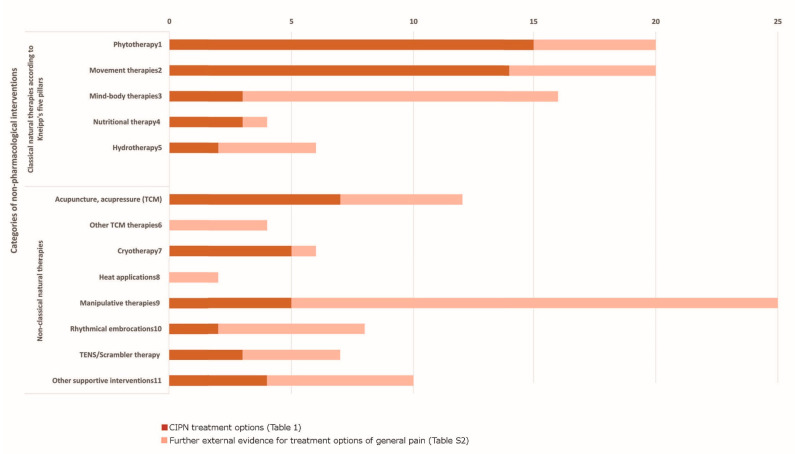
Overview of number of evidence sources for CIPN and general pain.

Key findings of the included studies are presented in Table 1 including a condensed result presentation of the review and the consensus process. The classification of *classical natural therapies* according to Kneipp and *non-classical natural therapies* [44] was applied to further categorize and analyze the complementary therapies that were reported in the studies. In total, 13 distinct categories were identified (Figure 2). 

Details of the interventions examined in the included studies can be found in Tables S1–S3; Table S1 (“Characteristics of selected studies included in the scoping review (nursing interventions for CIPN)”) and S2 (“Characteristics of other relevant studies for treating pain with complementary therapies in cancer patients of other patient populations”) can be found in Appendices 3 and 4). Overall, the study quality ranged from excellent (*n* = 29) [15,17,35,37,41,42,45,46,47,48,49,50,51,52,53,54,55,56,57,58,59,60,61,62,63,64,65,66] to satisfactory (*n* = 8) [19,67,68,69,70,71,72,73] and poor (*n* = 1) [74], while most studies had good study quality (*n* = 37) [16,18,38,39,40,43,75,76,77,78,79,80,81,82,83,84,85,86,87,88,89,90,91,92,93,94,95,96,97,98,99,100,101,102,103,104,105] (see Table S3 in Supplementary S5). 

**Figure 3:**
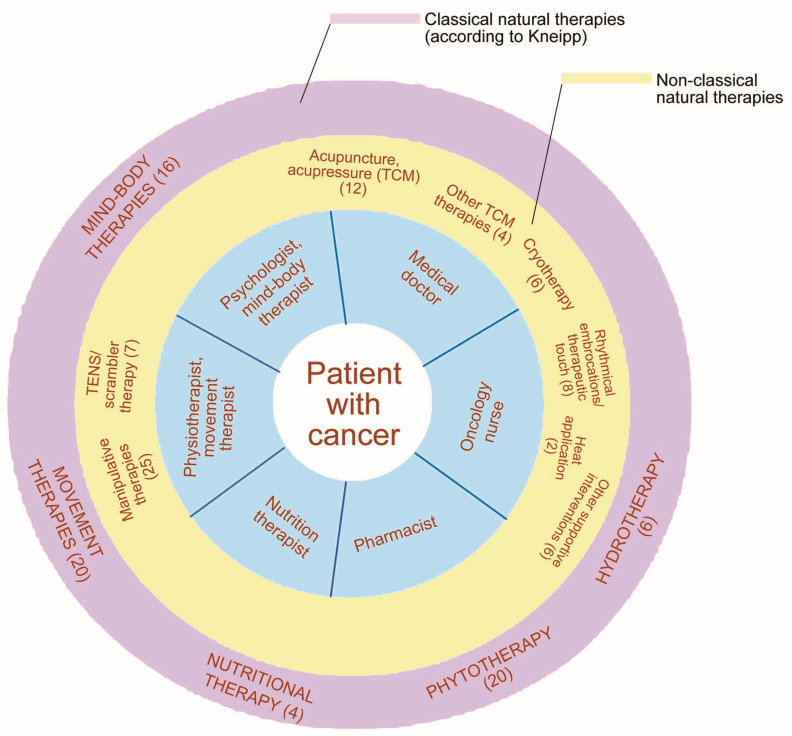
Interprofessional CIPN symptom management. Legend. Number in brackets refer to external study evidence and results of the consensus process (Tables S1, S2 and S5).

Figure 2 illustrates the evidence (Tables S1, S2 and S5) by referring to the number of studies/expert recommendations that the authors included for the 13 categories of non-pharmacological treatment options, which can be administered by doctors, oncology nurses, psycho-oncologists, physiotherapists, and all other members of interprofessional healthcare teams. Figure 3 visualizes how the different treatment options relate to the different health professionals. In 38 out of 75 of the studies, statements were made on the conceptual therapeutic approach. In the following, the evidence for the top seven most frequently identified complementary procedures are described.

Legend of Figure 2.

− 1 incl. aromatherapy, topical therapy, no oral phytotherapeutics, and flaxseed bath;
− 2 incl. physical therapy, sensorimotor training, exercise, closed kinematic chain exercise, resistance training, cardiovascular exercises, walking, cycling, whole-body-vibration, passive mobilization, coordination training, and tactile stimulation;
− 3 incl. relaxation, PMR, yoga, meditation, hypnosis, guided imagery, cognitive therapies, and distraction therapy, as well as Qi Gong and Tai Chi;
− 4 incl. vitamin and mineral supplements and dietary modification;
− 5 incl. alcaline bath and cold knee and/or arm showers;
− 6 incl. Tai Chi, Qi Gong, and massage acc. to TCM;
− 7 incl. cryocompression, cold applications, and hypothermia;
− 8 incl. hyperthermia;
− 9 incl. massage, reflexology, and foot reflexology;
− 10 incl. healing touch, Reiki, and therapeutic touch;
− 11 incl. compression, cupping (draining procedures), hydroelectric bath, music therapy, support groups, patient education, and nurse-led follow-up.
− *Note.* Study quality of the included studies varied—see Table S3 (Supplementary S5) for critical appraisal.

**Table 1:** Summary of studies and results of consensus process regarding the options (O) for prevention (p) or/and treatment (t) of CIPN.

O for p or t	Author ^1^	Study Design ^2^	p ^3^	t ^3^	Intervention	Outcome Measures	Result/Clinical Experience (CE) ^4^
Phytotherapy	Arslan et al. 2020 [17]	RCT (*n* = 60)	√	-	Henna application	CIPN assessment tool	Significant beneficial effect. Low cost, safe intervention, and well tolerated by patients.
	Fallon et al. 2015 in S3 clinical guideline Supportive therapy [37]	Proof of concept study	-	√	Application of menthol crème 1%	Brief Pain Inventory (BPI), Quantitative Sensory Testing	Significant reduction in pain symptoms.
	Izgu et al. 2019 [101]	Pilot RCT (*n* = 46)	-	√	Aroma hand and foot massage.	Neuropathic symptoms, numeric rating scale	Significant lower severity of pain based on NRS.
	Li et al. 2019 [35]	Meta-analysis	√	√	All types of Chinese herbal medicine in TCM	CIPN grade, Levi’s grade	Improvement of sensory nerve conduction velocity and motor nerve conduction velocity.
	Noh et al. 2018 [36]	Syst. Review of RCTs (*n* = 28)	√	√	All types of Phy used for medicinal purposes	Clinical improvement, nerve conduction study (NCS)	Potentially preventive and/or therapeutic effects for CIPN
	Noh and Park 2019 [50]	RCT (*n* = 31)	-	√	Aroma foot reflexology	CIPN assessment tool	Statistically significant reduction of symptoms.
	Rostami et al. 2019 [75]	RCT (*n* = 34)	-	√	Topical c. colocynthis oil	Functional Assessment of Cancer Therapy (FACT), Neurotoxicity score	Failed to improve the symptoms of CIPN compared with placebo.
	Consensus process	N/A	√	√	Aconit oil application	Clinical improvement	CE 3
	Consensus process	N/A		√	Solum oil application	Clinical improvement	CE 1
	Consensus process	N/A	√	√	Flaxseed bath	Clinical improvement	CE 4
	Consensus process	N/A	√	√	Arnica comp/Formica oil application	Clinical improvement	CE 3
	Consensus process	N/A	-	√	Arnica comp/Formica ointment (for stronger effect of Aconit)	Clinical improvement	CE 3–4
	Consensus process	N/A	-	√	Rosemary ointment	Clinical improvement	CE 3–4
	Consensus process	N/A	-	√	Peppermint oil application for heat sensations and paraesthesia	Clinical improvement	CE2
	Consensus process	N/A	-	√	Eucalyptus oil application for heat sensations and paraesthesia	Clinical improvement	CE 2
Movement therapies	Andersen et al. 2020 [38]	Single-blind ex-ploratory RCT (*n* = 48)	√	√	Physical therapy	Patient questionnaires, quantitative sensory testing	Improvement of CIPN pain for patients with breast cancer. Correlation to preservation of sensory function.
	Brami et al. 2016 [16]	Systematic review of RCTs (*n* = 13)	-	√	Physical activity	Nerve conduction velocity, (NCV), Neurological Symptom Score, Total Neuropathy Score, QoL	Evidence was reported for interventions consisting of physical activity components; for strength and endurance training; and for multimodal self-help strategies including physical activity, yoga, and mindfulness.
	Fernandes and Kumar 2016 [69]	Single-group pre-post prospective study (*n* = 25)	-	√	Closed kinematic chain exercise	Modified Total Neuropathy Score (mTNS), Berg Balance Score (BBS)	Significant change in values before and after the exercise.
	Kanzawa-Lee et al. 2020 [54]	Comprehensive inte-grative review(7 RCTs, 6 quasi-experimental studies)	-	√	Exercise with Aerobic, strength training, and balance training	CIPN, balance, and fitness	Empirical evidence is insufficient to definitively conclude that exercise interventions ameliorate CIPN.
	Kleckner et al. 2018 [48]	Secondary analysis of a phase III RCT (*n* = 355)	-	√	EXCAP©^®^ a standardized, individualized, moderate-intensity, home-based, six-week progressive walking and resistance exercise program	Patient-reported CIPN symptoms	Reduction of CIPN symptoms (hot/coldness in hands/feet, numbness, and tingling).
	McCrary et al. 2019 [84]	Prospective pilot intervention study, single group pre-post design (*n* = 35)	-	√	8-week multimodal exercises (resistance, balance, cardiovascular training)	Total Neuropathy Score—clinical version (TNSc), EORTC CIPN-20, functional assessment tools, disability, and QoL	Reduction of CIPN symptoms and related functional and quality of life deficits. No changes in sensory or motor neurophysiologic parameters.
	Schönsteiner et al. 2017 [89]	Randomized exploratory phase 2 study (*n* = 131)	-	√	Whole-body vibration including massage, passive mobilization, and physical exercise.	Functional Assessment of Cancer Therapy/Gynecologic Oncology Group neurotoxicity subscale (FACT/GOG-NTX), EORTC QLQ-C30 Quantitative sensory testing (QST)	Significantly and clinically relevant beneficial impact on symptom relief, physical fitness, and sensory function.
	Schwenk et al. 2016 [90]	Single blinded, randomized controlled pilot study (*n* = 22)	-	√	Interactive motor adaptation balance training program	VPT score, numeric rating scale for pain (NRS), neuropathy-related numbness in feet (NRS score), Short-Form Health Survey (SF-12), Falls, Efficacy Scale-International (FES-I)	Significant reductions in postural sway parameters in challenging semi-tandem position. No significant changes were noted for balance with “eyes closed”, gait speed, and fear of falling.
	Steinmann et al. 2011 in S3 clinical guidelineS3 Guideline Supportive therapy 2020 [37]	Overview article	√	√	Tactile Stimulation (e.g., been bath)	Clinical improvement	81% of patients consider tactile stimulation to be very effective or effective.
	Streckmann, Kneis et al. 2014 in S3 Guideline Supportive therapy 2020 [37]	RCT (*n* = 62)	-	√	Exercise (sensorimotor training, endurance, strength)	QOL; coordination, endurance, strength, therapy-induced side-effects.	Due to the highly significant physiological parameters, the study was terminated prematurely.
	Streckmann, Zopf et al. 2014 [60]	Systematic review of RCTs (*n* = 10), CCT (*n* = 8)	-	√	Exercise interventions	Side effects of Polyneuropathy	Number of patients with reduced deep sensitivity could be diminished. Only one RCT related to CIPN.
	S3 Guideline Supportive therapy 2020 [37]	S3 Guideline	-	√	Non-drug methods	Not described	Sensorimotor training and whole-body vibration represent new options for CIPN treatment. Clear evidence of improvement of functional limitation through non-medicinal procedures such as sports therapy, occupational therapy, physiotherapy, and physical therapy including electrotherapy.
	Tofthagen et al. 2012 [96]	Review of RCTs (*n* = 10), single-arm study (*n* = 1), cross-over-study (*n* = 1), quasi-experimental study (*n* = 1)	-	√	Strength training and balance training	Neuropathy symptoms, strength, balance	Recommendation of physical therapy as a treatment option, but no studies were identified that evaluate strength training and balance training for treatment of CIPN.
	Zimmer et al. 2018 [94]	RCT (*n* = 30)	-	√	Multimodal exercise program, (endurance, resistance, balance, coordination)	Trial Outcome Index (TOI),NCI-CTC/FACT/GOG-NTX	Regarding CIPN (TOI), there were significant differences between groups in the main analysis.
	Consensus process	N/A	-	√	Sugar oil peeling	Clinical improvement	CE 3
	Consensus process	N/A	√	√	Tactile stimulation	Clinical improvement	CE 2–3
Mind-body therapies	Brami et al. 2016 [16]	Systematic review of RCTs (*n* = 13)	-	√	Mind-Body modalities	NCV, Neurological Symptom Score, Total Neuropathy Score, QoL	Evidence was reported for self-management strategies including yoga and mindfulness.
	Galantino et al. 2019 [80]	Open-label, single-arm, feasibility trial	-	√	Yoga, Meditation	Functional Reach, Timed Up and Go, Patient Neurotoxicity Questionnaire (PNQ), (FACT-GOG-NTX)	Significant improvements were found in flexibility, balance, and fall risk.
	Kanzawa-Lee et al. 2020 [54]	Comprehensive inte-grative review(7 RCTs, 6 quasi-experimental studies)	-	√	Yoga, exercises	CIPN, balance, and fitness	Empirical evidence is insufficient to definitively conclude that exercise interventions ameliorate CIPN.
Nutritional therapy	Brami et al. 2016 [16]	Systematic review of RCTs (*n* = 13)	√	√	Glutamine, Goshajinkigan, vitamin E, Omega 3, Acetyl-l-carnitine, Alpha-lipoic-acid	NCV, Neurological Symptom Score, Total Neuropathy Score, QoL	Vitamin E, Glutamine, Goshajinkigan, and Omega-3 may prevent CIPN. Acetyl-l-carnitine may worsen CIPN; Alpha-lipoic-acid activity is unknown.
	Greenlee et al. 2017 [42]	Clinical practice guideline based on a systematic literature review of RCTs.	√	√	Omega-3, fatty acids, vitamin E, alpha-lipoic acid, dietary modification	-	Acetyl-l carnitine is not recommended to prevent CIPN. Insufficient evidence that Omega-3, fatty acids, and vitamin E help to reduce neuropathy.
	Rostock et al. 2013 [88]	Four arm RCT (*n* = 60)	-	√	Vitamin B complex	Detailed questionnaire, NRS	Positive effects. No statistically significant results.
Hydrotherapy	Consensus process	N/A	-	√	Alkaline bath for hand/foot, then Aconit oil application	Clinical improvement	CE 3
	Consensus process	N/A	-	√	Cold knee and/or arm showers	Clinical improvement	CE 3
Acupuncture/Acupressure	Brami et al. 2016 [16]	Systematic review of RCTs (*n* = 13)	-	√	Electroacupuncture	NCV, Neurological Symptom Score, Total Neuropathy Score, QoL	Not superior to placebo.
	Deng et al. 2013 [53]	Systematic review of meta-analyses (*n* = 4), syst. Reviews (*n* = 14), RCT (*n* = 16)	-	√	Acupuncture	VAS, neuropathy symptoms, QoL.	Some improvement regarding VAS and neuropathy symptoms.
	Donald et al. 2011 [68]	Retrospective Evaluation (*n* = 18)	-	√	Acupuncture	CIPN symptoms.	82% (*n* = 14) reported improvement of neuropathy symptoms.
	Greenlee et al. 2017 [42]	Clinical practice guideline based on a systematic literature review of RCTs.	√	√	Acupuncture, electroacupuncture	-	Insufficient evidence that electroacupuncture help to reduce neuropathy.
	Rostock et al. 2013 [88]	Four arm RCT (*n* = 60)	-	√	Electroacupuncture	Detailed questionnaire, NRS Scale	Positive effects. No statistically significant results.
	S3 guideline complementary medicine in the treatment of oncology patients [57]	S3 guideline	-	√	Acupuncture, electroacupuncture	BPI, Total Neuropathy Score, NCS, Functional Assessment, QoL.	Data are available from a meta-analysis and two RCTs on the efficacy of A- for CIPN.
	Wong et al. 2016 [93]	Prospective phase 2 study (*n* = 40)	-	√	Acupuncture like TENS	Numbness score, mTNS, Edmonton Symptoms Assessment Scale (ESAS)	Statistically significant difference at 6 months from the baseline pain score.
Cryotherapy	Bandla et al. 2020 [18]	Proof-of-concept study (*n* = 26)	√	-	Cryocompression	Total neuropathy score (TNS), NCS	Potentially improve efficacy of preventing CIPN. Safe and tolerable.
	Beijers et al. 2020 [39]	RCT (*n* = 180)	√	-	Frozen glove and sock	CIPN20	Significant reduction of CIPN symptoms. Dropout of one-third of patients.
	Griffiths et al. 2018 [19]	RCT (*n* = 29)	√	-	Frozen glove and sock	Neuropathic Pain Symptom Inventory, BPI.	No significant differences in neuropathy or pain. Drop-out rate, more than 50 %.
	Sundar et al. 2017 [40]	Prospective pilot study (*n* = 20)	√	-	Continuous-flow limb hypothermia.	Visual analog scale (VAS), subjective tolerance scale, NCS,	No significant difference in NCS. Well tolerated by all patients.
	Consensus process	N/A	√	-	Frozen gloves and socks	Clinical improvement	Cannot be assessed.
Manipulative therapies	Brami et al. 2016 [16]	Systematic review of RCTs (*n* = 13))	-	√	Massage, touch therapy	MD Anderson Symptom Inventory	Greatly reduced CIPN symptoms from grade 2 to 1 and significantly improved quality of life.
	Cunningham et al. 2011 [74]	Case report	-	√	Massage	MD Anderson Symptom Inventory	Greatly reduced CIPN symptoms from grade 2 to 1 and significantly improved quality of life.
	Izgu et al. 2019 [41]	RCT (*n* = 40)	√		Massage	Self-Leeds Assessment of Neuropathic Symptoms and Sign (S-LANSS), EORCT QLQ CIPN20, NCS.	Massage successfully prevented CIPN, improved the QoL, and showed beneficial effects on the NCS findings.
	Sarisoy, et al. 2020 [76]	RCT (*n* = 40)	-	√	Foot-massage	VAS, Doleur Neuropatique/Neuropatic pain (DN4), Pittsburg Sleep Quality Index (PSQI)	Positive effect on CIPN pain.
	Schönsteiner et al. 2017 [89]	Randomized exploratory phase 2 study (*n* = 131)	-	√	Whole-body vibration including massage, passive mobilization, and physical exercise.	(FACT/GOG-NTX), EORTC QLQ-C30 Quantitative sensory testing (QST)	Significantly and clinically relevant beneficial impact on symptoms relieve, physical fitness, and sensory function.
Rhytmical embrocations	Consensus process	N/A	-	√	Aconit oil—rhythmical embrocation	Clinical improvement	CE 4
	Consensus process	N/A	-	√	Arnica comp/Formica oil—rhythmical embrocation	Clinical improvement	CE 4
TENS/Scrambler therapy	Coyne et al. 2013 [67]	Expanded trial, single arm trial (*n* = 39)	-	√	Scrambler therapy	NRS, BPI, European Organization for Treatment and Cancer CIPN20 (EORTCCIPN20)	Clinically important and statistically significant improvements were seen in average, least, and worst pain.
	Gewandter et al. 2019 [65]	Single-arm study (*n* = 29)	-	√	TENS	EORTC-CIPN20, Utah Early Neuropathy Score	Significant improvements were observed with the EORTC-CIPN20.
	Loprinzi et al. 2020 [71]	RCT, two arm phase II pilot trial (*n* = 50).	-	√	Scrambler therapy, TENS	EORTC CIPN20, NAS questionnaire regarding CIPN-associated pain	Scrambler therapy improves CIPN symptoms more than TENS.
Other supportive interventions	Kotani et al. 2021 [43]	Double-blind phase 2 trial (*n* = 56)	√	-	Compression	Incidence of Grade ≥ 2 CIPN.	No significant reduction of CIPN incidence.
	Rostock et al. 2013 [88]	Four arm RCT (*n* = 60)	-	√	Hydroelectric bath	Detailed questionnaire, NRS	Positive effects. No statistically significant results.
	Consensus process	N/A	√	-	Compression	Clinical improvement	Cannot be assessed.
	Consensus process	N/A	-	√	Copper ointment (0.4%)	Clinical improvement	E 2

Legend. ^1^ The number in brackets refer to the comprehensive reference list (see full article). ^2^ N/A: not applicable. ^3^ √: meets criteria; -: does not meet criteria, ^4^ CE = Clinical experience (rated on a numerical scale 0 to 5 with 0 = no effect and 5 = maximum effect.

### 3.5. Manipulative Therapies

This section reports the results on non-classical naturopathic treatments of manipulative therapies, which include massage (M), reflexology (R), and R of the feet. The medical literature on M therapy yielded 25 results, of which 5 studies were especially related to the therapy of CIPN [16,41,74,76,89] and 19 studies focused on cancer pain [15,45,46,49,51,56,58,63,64,66,77,78,83,87,99,100,102,103,105] and 1 [72] on any medical condition, which may result from the presence of CIPN. Within the 25 studies, there are 10 RCTs [41,45,46,49,76,77,78,83,87,89], 11 reviews (including systematic reviews/meta-analysis) [15,16,51,56,58,64,72,99,100,102,103], 1 case report [74], 1 quasi-experimental study [105] and 2 recommendations from guidelines [63,66]. The quality of most studies, including the two guidelines, were rated excellent (*n* = 11) [15,41,45,46,49,51,56,58,63,64,66] according to the CASP scheme [106] or good (*n* = 12) [16,76,77,78,83,87,89,99,100,102,103,105]. One study was rated satisfactory (*n* = 1) [72] and the case report [74] was rated as poor in terms of quality. More details on the assessment of study quality according to CASP can be found in the Supplementary S5 (Table S3).

#### Massage, Reflexology, and Foot Reflexology

During M, mechanical touch techniques are used to act on the muscles and connective tissues. The main purpose of classical M is said to be the treatment and prevention tension; the stimulation of blood and lymphatic circulation and metabolism; and the influence on circulation, blood pressure, respiration, and psyche. R is a form of M in which pressure is applied to the hands and feet [49,107]. Pain reduction through M is explained by desensitization of nociceptors [83].

Of the five studies with focus on CIPN, three assessed classical M. Classical M significantly prevented CIPN and neuropathic pain as well as improved nerve conduction and QoL when compared to usual care at week 12 (*n* = 1 study; *n* = 40 participants receiving adjuvant paclitaxel for breast cancer; intervention duration: 12 weeks) [41]. In one case report, which is also reported in a systematic review [16], classical M is associated with greatly reduced CIPN symptoms from grade 2 to 1 and markedly improved quality of life [74]. In one RCT, foot M was shown to reduce patients’ pain scores and have a positive effect on sleep quality, compared to clinical routine (*n* = 40 patients with non-Hodgkin’s lymphoma; intervention duration: four weeks) [76]. A program including M and mobilization as well as physical E and WBV had a significantly and clinically relevant beneficial impact on symptom relief, physical fitness, and sensory function (*n* = 1 study; *n* = 131 participants; intervention duration: 15 weeks) [89].

Of the four RCTs that assessed classical M for cancer pain [49,83,87,105], two found no statistically or clinically improvement in pain (*n* = 2 studies; *n* = 610 participants with cancer pain; intervention duration: two and four weeks) [49,87] and one found significantly higher reduction of physical discomfort in IG compared to routine health care (*n* = 1 study; *n* = 86 participant; intervention duration: five weeks) [83]. Foot massage showed positive effects on pain (*n* = 1 study; *n* = 87; intervention duration: three consecutive days) [105]. 

The four RCTs that assessed R in cancer patients [45,46,77,78] reported heterogeneous effects regarding pain. R was found to have positive effect on pain and overall well-being compared to aromatherapy-M (*n* = 1 study, 115 participants, intervention duration: four treatments) [46]. Reflexology complemented by PMR exercises was found to decrease pain and fatigue and increase quality of life (*n* = 1 study; *n* = 80 participants, intervention duration: eight weeks) [45]). No statistically significant effect could be shown for pain in cancer patients treated with foot R (*n* = 1 study; *n* = 36 participants; intervention duration: two times, 24 h apart) [77]. In contrast, an immediate positive effect of foot R for patients with metastatic cancer who reported pain was found (*n* = 1 study; *n* = 256 participants; intervention duration: four weeks) [78]. Evidence for R as a treatment for any medical condition could not be demonstrated convincingly in a systematic review (*n* = 18 RCTs; *n* = 949 participants) [72].

In the nine reviews and meta-analyses that assessed M or R in cancer patients [15,51,56,58,64,99,100,102,103], studies with different types of massage and reflexology were included. The results were heterogeneous. Improvement of pain through M was reported (*n* = 2 studies; *n* = 30 RCTs; *n* = 4448 participants) [56,58] as well as through foot reflexology (*n* = 1 study; *n* = 12 RCTs; *n* = 559 participants) [56]. Weak recommendations are suggested for M, compared to an active comparator, for the treatment of pain, fatigue, and anxiety. No recommendations were suggested for M therapy compared to no treatment or sham control (*n* = 1 study; *n* = 16 RCTs) [51]. Beneficial effects of classical M for pain of any origin are reported in a nursing guideline [66]. Due to the heterogeneous data from RCTs on the effectiveness of classical M in reducing pain in oncology patients, no recommendation can be made for or against the use of classical M to reduce pain in another guideline [63].

### 3.6. Rhythmical Embrocations (Including Healing Touch, Therapeutic Touch, Reiki)

RE are a gentle, gliding touch along archetypal forms of the body [108]. It is believed that RE strengthen life forces, enhance warmth, and harmonize rhythmic processes in the body. For application, RE utilizes massage oils and plant or metal ointments. Therapeutic touch (TT) describes a method of laying hands on a person by a therapist. Thereby it is assumed that there are human energy fields, which are in constant interactions with each other and with their environment. The therapist’s goal is to bring these energy fields back into balance [63,81,104].

The medical literature yielded six results of which five were related to cancer-related symptoms [15,63,81,87,104] and one to low back pain [86]. Among the studies, there was one systematic review [15], three RCTs [81,87,104], one guideline [63], and one observational study [86]. The quality of two studies [15,63] was rated excellent according to the CASP scheme, and four studies were rated good [81,86,87,104].

According to expert consensus, RE increases the effectiveness of two nursing interventions for CIPN (Aconit oil, Arnica comp. Formica oil) by one point on the five-point Likert scale from three to four (Table S4, Supplementary S6). Three RCTs assessed TT or healing touch (HeTo) in cancer patients. HeTo as well as classical M are more effective than presence alone or standard care in reducing pain, mood disturbance, and fatigue in patients receiving cancer chemotherapy (*n* = 1 study, *n* = 230 participants, intervention duration: 4 weeks) [87]. TT led to significantly higher well-being compared to rest period (*n* = 1 study; *n* = 20 participants, intervention duration: 4 consecutive days) [81]. TT significantly decreased pain and fatigue more than usual care, while the placebo group indicated a decreasing trend in pain and fatigue scores compared with the usual care group (*n* = 1 study; *n* = 90 participants; intervention duration: 5 days) [104]. One guideline states that the quality of the included studies [109,110,111] on TT is too low to provide meaningful results [63].

HeTo for cancer patients was assessed in one systematic review. HeTo seems promising, particularly in the short term, but cannot be recommended because of a paucity of rigorous trials. Future research should focus on methodologically strong RCTs to determine potential efficacy of these CAM interventions [15]. RE with Solum oil was shown in an observational study to be a promising and useful complementary method for the treatment of chronic low back pain [86].

### 3.7. Phytotherapy (including Herbal Medicines)

Phy is one of the oldest medical therapies and is indigenous to all continents and cultures. While Western medicine and traditional Persian medicine tend to use single medicinal plants, in Traditional Chinese Medicine (TCM), for example, combinations or mixtures of more than three herbal drugs are common [112]. This category includes most types of external applications (EAP). These are applications from aromatherapy (AT) (e.g., Aconit oil application), topical therapy (ToT) (e.g., Citrullus colocynthis), phytotherapy (Phy), or herbal medicine (e.g., flaxseed bath, astragalus, henna). Eleven nursing interventions recommended by the expert panel can be assigned to this category (see Table S4, Supplementary S6). Some nursing interventions combine the types of EAP, such as RE and AT (Aconit oil—therapeutic application) or hydrotherapy (HTK) and phytotherapy (Phy) (flaxseed bath).

Eight nursing interventions were recommended by the expert panel based on Phy (Solum oil application; flaxseed bath; Arnica comp/Formica oil application; Arnica comp/Formica oil—therapeutic application; Arnica comp/Formica ointment; Aconit oil application; Aconit oil—therapeutic application; alkaline bath for hand/foot, then Aconit oil application). In anthroposophic medicine and nursing, Aconite oil is considered a substance that can compensate for overstimulation of nerves and senses in neuropathic pain by its warming and relaxing agents. Solum oil forms a protective thermal mantle that prevents overstimulation of nerves and senses. Except for Solum oil, all interventions are rated 3 or 4 on the five-point Likert scale. Aconit oil application is used most frequently (five out of six institutions).

The literature search yielded seven results related to CIPN [17,35,36,37,50,75,101] and five results related to other types of pain [15,46,47,56,86]. Among the studies, there were two meta-analyses [35,56], two systematic reviews [15,36], five RCTs [17,46,47,50,75], one pilot CCT [101], one observational study [86] and a S3 guideline [37], which reports a non-randomized pilot study. The quality of nine studies was rated excellent according to the CASP scheme [15,35,36,37,46,47,50,56,75], and three studies were rated good [17,86,101]. More details on the assessment of study quality according to CASP can be found in the Supplementary S5 (Table S3).

Henna application (HA) on hands and feet for women under Oxaliplatin treatment showed significant beneficial effect on CIPN. The procedure is considered inexpensive and well tolerated (*n* = 1 study; *n* = 60 participants; intervention duration: three 15-day treatment courses) [17]. All types of Phy (e.g., hand/foot bath, compress) used in TCM for reducing CIPN were assessed in the meta-analysis. Herbs that activate blood, improve microcirculation, and dilate collaterals (e.g., astragalus, ginger) were found to have potential healing effects as well as improvement in sensory nerve conduction velocity (SNCV) and motor nerve conduction velocity (MNCV). This was found for all grades of CIPN for preventive and curative treatment, even though more research is needed (*n* = 20 studies; *n* = 1481 participants) [35]. In another review, some types of Phy were found to have potentially preventive and/or therapeutic effects for CIPN. Due to the characteristics of CIPN, the direct application would be considered an effective dosage form (*n* = 28 studies; *n* = 2174 participants) [36].

The safety and efficacy of topical Citrullus colocynthis (bitter apple) oil in the management of CIPN was evaluated in a RCT. No significant improvement could be shown. The intervention failed to improve the symptoms of CIPN compared with placebo (*n* = 1 study; *n* = 18 participants, intervention duration: two months) [75]. Solum oil administered as RE was shown in an observational study to be a promising and useful complementary method for the treatment of chronic low back pain [86].

#### Aromatherapy, Aromatherapy Massage, and Aromatherapy Reflexology

Aromatherapy (AT) is a part of Phy and deals with the application of essential oils with physical, psychosomatic, psychological, and physiological effects [113], which are processed in the limbic system [47,114,115]. 

Aromatherapy massage (AT-M) or reflexology (AT-R) combines two therapeutic approaches. The systematic, controlled application of essential oils through AT with the application of specific physical manipulations to the soft tissues of the body through M or stimulation of specific trigger points through reflexology (R). Three nursing interventions recommended by the expert panel focused on AT (rosemary ointment, peppermint oil for heat sensations and paresthesia, and eucalyptus oil application for heat sensations and paresthesia). These are used in one of the six institutions. Rosemary oil was rated between three and four on the five-point Likert scale, while peppermint oil and eucalyptus oil were rated two.

Compared to standard medical care, aromatherapy hand and foot massage (AT-M) significantly reduced incidence of neuropathic pain at week six; severity of neuropathic pain at weeks two, four, and six; and fatigue at week eight (*n* = 1 study; *n* = 46 participants with CIPN and fatigue receiving Oxaliplatin; intervention: 18 sessions of 40 min over six weeks) [101]. AT-R conducted by patients themselves resulted in statistically significant reduction of PNP symptoms in a RCT with pre-post design (*n* = 1 study, *n* = 63 participants, intervention duration: six weeks) [50]. 

One proof of concept pilot study (Fallon et al. 2015), discussed in the S3 guideline supportive therapy [37], showed significant reduction in CIPN pain symptoms plus an improvement in functionality and sensitivity by application of Menthol crème 1%. AT-M was shown to significantly decrease neuropathic pain severity and quality of life (QoL) in diabetic patients (*n* = 1 study; *n* = 46 participants; intervention duration: four weeks) [47]. Various types of massage were assessed in a meta-analysis [56]. AT-M had significant effects on cancer pain in two studies, but pain relief was short-lived (two weeks) (*n* = 12 RCTs; *n* = 559 participants) [56]. The beneficial effects of AT-M on self-selected problems measured by MYCaW score have been confirmed by a non-blinded, randomized study for patients with cancer (*n* = 1 study; *n* = 115 participants; intervention duration: four treatments) [46]. Bardia et al. investigated in their systematic review on complementary treatments (CTs) for cancer-related pain. One study with high quality based on the Jadad score added lavender AT to M and found no difference in effect on pain (*n* = 18 RCTs; *n* = 1499 participants) [15].

### 3.8. Movement Therapies

Movement therapy (MT) applies body movement that can help persons to cope with physical or mental illnesses, disabilities, or life challenges. The aim of MT, which can include various physical activities, is to enhance the person’s cognitive, physical, mental, social, and emotional well-being. There have been multiple studies investigating the effect of different forms of physical activity and MT on CIPN. They are thought to attenuate CIPN through its influence on blood circulation, inflammation, pain-inhibiting neurotransmitters, endogenous opioids, and coping and symptom interaction mechanisms [48,54]. 

In total, evidence was found for 18 studies with different study designs and different forms of exercises (Es), i.e., cardiovascular E, coordination training, cycling, closed kinematic chain Es, passive mobilization, resistance training, sensorimotor training, whole-body-vibration, and walking. Most studies were reviews (*n* = 8) [54,57,60,64,96,97,100,102] or were conducted with controlled designs (*n* = 6) [38,48,69,84,89,94], and clinical guidelines were also considered (*n* = 3) [37,66,98]. Study quality ranged from satisfactory (*n* = 1) [69] to good (*n* = 11) [38,84,89,90,94,96,97,98,100,102] to excellent (*n* = 7) [37,48,54,57,60,64,66]. Most of the included E studies focused on examining CIPN for patients with cancer (*n* = 12) [37,38,48,54,64,66,69,89,94,96,98,102], while some studies exclusively examined patients with breast cancer (*n* = 3) [38,48,64], patients with colorectal cancer (*n* = 1) [94], or patients with lymphoma (*n* = 1) [60], and two studies specifically focused on cancer survivors [84,98]. Some of the included studies (*n* = 3) focused on improving general pain in patients with cancer [57,66,100], while some studies (*n* = 3) mainly included diabetic patients with neuropathies only [60,96,97].

Closed kinematic chain exercises (CKC-Es) are based on the concept of moving specific joints and segments, creating a chain of events that affects the movement of neighboring joints of segments. By performing CKC-Es, the hand or foot is in contact with the surface on which one practices (e.g., squats, lunges, deadlifts, power cleans, leg presses, push-ups and derivates; pull-ups or chin-ups; and dips). CKC-Es significantly decreased the modified total neuropathy score (mTNS) and significantly increased the Berg Balance Score (BBS) in a single-group pre-post prospective study (*n* = 1 study; *n* = 25 individuals with CIPN; intervention duration: 15 sessions over 3 weeks) [69]. Future research might investigate on long-term clinically significant effects and consider larger sample sizes.

An interactive sensor-based balance training (BT) significantly reduced a sway of hip, ankle, and center of mass when compared to a CG (*n* = 1 study; *n* = 22 participants; intervention duration: two sessions per week (45 min) for four weeks) [90]. The challenges of postural performance, by coordinating the ankles as well as the dynamic weight shifting, may have contributed to those effects and can be further assessed in larger trials. The beneficial effects of BT have been confirmed in a systematic review on exercise intervention studies for neuropathic patients [60]. The results indicate that BT have a stronger effect on peripheral neuropathies than exercise intervention focusing on strength or endurance or both. Only one included study (Streckmann, Kneis et al. 2014) in the review by Streckmann, Zopf et al. [60] and referred to in one clinical guideline [37] focused specifically on CIPN and indicated that BT and in particular sensorimotor training (SMT) may have the most beneficial effect for supporting patients with cancer during therapy for improving quality of life outcomes and also for improving their peripheral deep sensitivity.

Physical therapy significantly decreased CIPN when compared to a CG after chemotherapy and three months post-chemotherapy (*n* = 1 study; *n* = 48 breast cancer patients (stage I-III); intervention duration: four visits by a physical therapist to develop a home E and education program at the beginning of chemotherapy) [38]. Nerve gliding Es (for elongating the nerve) were completed three times daily for five to ten minutes and not only significantly reduced CIPN, but also significantly improved pain pressure threshold and grip dynamometry.

The beneficial effects of exercises have been confirmed by another study assessing a multimodal exercise intervention consisting of resistance training (RT), balance training (BT), and cardiovascular exercises (CardEs) (*n* = 1 study; *n* = 29 cancer survivors; intervention duration: three times per week for a duration of eight weeks) [84]. CIPN symptoms improved significantly from pre-E to post-E. Such findings are encouraging and could be confirmed by a larger trial. Current clinical guidelines for the supportive therapy for patients with cancer [37] and survivors [98] recommend E therapies as well.

An exercise program including progressive walking (W) and resistance training (RT) has an effect on patients’ CIPN symptoms [48] (*n* = 1 study; *n* = 355 individuals affected with cancer; intervention duration: six weeks). Compared to the CG, symptoms of hot/coldness in hands/feet and numbness and tingling were significantly reduced in the IG. This effect may have developed as exercises can reduce chronic inflammation, and inflammatory processes appear to play a role in the etiology and treatment of CIPN. Thus, the authors clearly call on interprofessional healthcare teams to prescribe exercise therapies for their patients.

A multimodal exercise program including endurance (EN), resistance (RT), and balance training (BT) on CIPN helped patients to keep their CIPN symptoms stable (*n* = 1 study; *n* = 24 metastasized colorectal cancer patients; intervention duration: two times per week for 60 min) [94]. Compared to the CG, patients in the IG did not experience a worsening of their symptoms. Another integrated exercise program including massage (M), passive mobilization (PM), and physical exercise (E) evaluated whole-body vibration (WBV) (by applying the vibration platform Galileo-Fitness) (*n* = 1 study; *n* = 131 patients with CIPN; intervention duration: 15 training sessions within 15 weeks) [89]. Patients in the WBV condition plus the integrated E program performed better with regard to the primary outcome, the chair-rising test (CRT) (a test where patients are asked to stand up from a chair and then cross their arms in front of the chest for five times as fast as possible). All patients completing the study experienced less symptoms and pain and improved their CRT over time. The authors conclude that this program could be well integrated into daily clinical practice, but a standardized assessment of CIPN is needed as well as adequate education of nursing staff. One review assessed WBV [97]. Of the five included studies, four studies were on diabetic peripheral neuropathy, and one study was on HIV-associated distal symmetrical polyneuropathy. Three of the five studies found a beneficial effect of WBV on neuropathic pain as well as for improvements in strength and balance; however, this was not confirmed by two others of the included studies. As the methodology for all included studies was reported to be low, and none was focused on patients with cancer or survivors, the authors conclude that there is a high need to further explore the effect of WBV in high-quality trials in cancer populations.

Those encouraging results and recommendations, however, have been dimmed by some reviews including a range of studies investigating if MT contribute to CIPN and pain relief. One current review synthesized evidence for the effects of exercise on CIPN symptoms [54], and only clinical trials and meta-analyses have been searched, so that the results yielded in 13 included studies investigating four different types of exercise (E): Yoga (Y), aerobic (Ae) E, strength training (STr), and balance training (BT). It was concluded that none of the studies met 100% of the CONSORT checklist criteria, and only two of the studies were considered as moderate-quality evidence. Even though all the seven studies demonstrated that AeE led to significant CIPN benefits, the authors recommended interpreting the results with caution and suggested that more evidence is needed to conclude that E interventions influence CIPN symptoms. Nevertheless, healthcare professionals including oncologists, oncology nurses, psycho-oncologists, nutritional therapists, physiotherapist, and pharmacists can inform and encourage patients and survivors of practicing physical E to improve their balance, fitness, and better manage their symptomatic burden. The authors of a Cochrane review [57], which already dates back several years, came to a similar conclusion, and due to a large heterogeneity of included E programs (40 trials on STr, resistance training (RT), walking (W), cycling (Cy), Y, QG, and TC), it was difficult to draw concrete conclusion and recommendations. In a systematic review of systematic reviews [64], effects of multiple rehabilitation interventions, including E and physical activity (PhA), complementary and alternative medicine, Y, lymphoedema treatment, and psychosocial interventions, could be demonstrated for general pain and other symptom outcomes. Here again, the effect concepts have not been differentiated. All 37 included studies were evaluated with the AMSTAR 2 tool, with 21 were having low, 14 having moderate, and 2 having high methodological quality. In other reviews, however, the general benefits of non-pharmacological interventions including physical therapy (PT) to reduce CIPN [102] and general pain [100] have been pointed out and are more comprehensible due to the applied categorization system.

One review assessed strength- and balance-training (STr and BT) programs in patients at high risk of falls [96]. Overall, 3 out of 13 studies found that Str and BT were safe and effective at reducing falls and improving strength and balance in adult patients with diabetes-related peripheral neuropathy. Future research could use this as a basis to conduct further studies with these safe and effective interventions for cancer patients with CIPN. One clinical guideline assessed preventative options for CIPN [37]. Regular MT, in particular for training the fingers and toes, as well as sensorimotor training (SMT) with bean baths, or electrotherapy with two- or four-cell baths (here one or two extremities—feet/lower legs or hands/forearms—are immersed in a water bath and a larger plate electrode—200 to 300 cm^2^—is placed lumbar or cervical, respectively) have been presented as non-pharmacological options for preventing CIPN. Tactile stimulation by application of special naturopathic procedures, such as beeswax kneading, hedgehog ball massage, and quartz sand baths, has also been recommended by the expert panel based on yearlong experience as options for early prevention and/or treatment of CIPN.

### 3.9. Mind–Body Therapies

According to the National Center of Complementary and Integrative Health (NCCIH), mind–body therapies (MBT) or modalities (MBM) are “practices that focus on the interactions among the brain, mind, body and behavior with the intent to use the mind to affect physical functioning and promote health” [116]. MBT focus on training the mind–body connection and include modalities as relaxation techniques, meditation, mindfulness-based therapies, body scanning, yoga, progressive muscle relaxation, guided imagery, autogenic training, hypnosis, biofeedback, and cognitive therapies. Existing evidence shows that mind–body therapies are frequently used in oncology and that they have an effect [117]. Patients use them to improve the quality of life, to strengthen their immune system, to reduce stress, and to stimulate hope. Smaller numbers of patients use these therapies to treat specific symptoms such as pain and fatigue [118].

The medical literature on MBT yielded 16 studies, of which 3 studies were on CIPN [16,54,80]. The other studies (*n* = 13) were focused on other pain treatment. The most frequently described MBM of the studies included was related to yoga (Y) [52,54,57,64,73,80,92], followed by distraction therapies (DT) [66,70,100,102] and relaxation (Rel) [15,66,70,95,100]. Further studies focused on progressive muscle relaxation (PMR) [45], cognitive behavioral interventions such as problem-solving therapies [79], meditation [80], self-management therapies [70], Qi Gong [70], Tai Chi [70], hypnosis [15], imagery [100], and mind–body practices [16]. Within the 21 results, there are 10 systematic reviews [15,16,52,54,57,64,73,95,100,102], 4 RCTs [45,70,79,92], 1 study with an open-label, single-arm, mixed-methods design [80], and 1 expert standard for pain management in nursing [66]. The quality of most studies was rated either excellent [15,45,52,54,57,64,66] or good [16,79,80,92,95,100,102]. The quality of two studies was assessed with satisfactory [70,73]. More details on the assessment of study quality according to CASP can be found in the Supplementary S5 (Table S3).

#### 3.9.1. Yoga

Yoga (Y) is an ancient Indian system focusing on the physical, mental, and spiritual practices with the aim to calm one’s thoughts and belief concepts. Thus, Y has a strong meditative component that also includes pranayamas, or breathing techniques.

An intervention consisting of somatic Yoga (Y) and meditation (Me) improved QoL, flexibility, and balance (*n* = 1 study; *n* = 10 participants with cancer survivors suffering of CIPN; intervention duration: twice a week for eight weeks for 1.5 h) [80]. Two systematic reviews assessed MBT. Furthermore, 1 of 13 RCTs in one systematic review [16] found MBT including self-management strategies like Y and Me to reduce CIPN symptoms. Another systematic review including seven RCTs and six quasi-experiments [54] recommended balance training including Y as promising preventive interventions and treatments for CIPN. Additionally, 16 studies assessed MBT for other pain and patient-reported outcomes. Y sessions improved psychological distress as well as fatigue, nausea and vomiting, pain, shortness of breath, insomnia, loss of appetite, and constipation compared to a CG at week 6 (*n* = 1 study; *n* = 40 breast cancer patients receiving adjuvant radiotherapy; intervention duration: three Y per week for six weeks) [92]. Two systematic reviews focused on Y practices. In nine RCTs, Y was compared with a CG and was found to significantly reduce distress, anxiety, depression, and moderately reduce fatigue, as well as moderately increase general HRQoL, emotional function, and social function in patients with breast cancer [52]. However, studies lacked long intervention durations; only two studies lasted 12 weeks or longer, all others were shorter and ranged from 6 to 10 weeks. Another systematic review of systematic reviews [64] recommended Y for treating anxiety during active cancer treatment and for improving fatigue, sleep disturbances, gastrointestinal symptoms, and depression. The practice of Y was recommended for at least 3 months. Another Cochrane review assessed exercise interventions including Y on HRQoL and associated outcomes in cancer survivors [57]. The totality of the movement interventions had an effect on HRQoL at 12 weeks and 6 months follow-up compared to a CG. Exercise interventions also reduced anxiety at 12 weeks follow-up, fatigue at 12 weeks and between 12 weeks and 6 months follow-up, and pain at 12 weeks follow-up compared to a CG. In another review educational article, Y was also recommended for patients with spinal cord injury (SCI) suffering of neuropathic pain (NP) [73]. Even though the pathogenesis of SCI induced NP is different compared to CIPN, the evidence shows that Y has also the potential to alleviate other pain types. 

#### 3.9.2. Distraction Therapies and Relaxation

By applying distraction therapy (DT) it is possible to divert one’s attention from an unpleasant experience and focus on a positive sensory or cognitive stimulus instead. Computational tasks, music, images of nature, and inhaling of aromas can reduce the pain experience by distracting and relaxing oneself [16]. Distraction therapy (DT) has been assessed (*n* = 4 studies) with mixed results. Pain scores decreased significantly in a single pre-test and post-test group (*n* = 1 study; *n* = 34 female cancer patients with breast or gynecologic cancer; intervention duration: participants were free to use it as often as needed) after the end of a 48-hour period [70,100]. Another educational review article [102] recommended to apply DT as a non-drug therapy for peripheral neuropathy; however, a reference specifically relating to CIPN was missing; thus, no concrete conclusion can be drawn. DT and relaxation (Rel) have also been recommended by a German expert standard for pain management in nursing with reference to one systematic review on non-pharmacological and non-invasive chronic pain interventions (Skelly et al. 2018) [66].

#### 3.9.3. Additional MBMs

##### Progressive Muscle Relaxation and Relaxation

Progressive muscle relaxation (PMR) and reflexology (R) significantly decreased pain intensity and fatigue and improved QoL compared with PMR alone (*n* = 1 study; *n* = 80 women with gynecologic cancer, intervention duration: two sessions totaling 60 min were conducted at each of the 16 home visits over 8 weeks) [45]. Two systematic reviews assessed non-pharmacological treatments for pain [15,95]. Overall, 3 of 18 RCTs in one systematic review found relaxation/imagery and hypnosis to have significant benefits on cancer pain management [15,95]. 

##### Problem-Solving Therapies

Problem-solving therapy (PST) significantly reduced symptom limitations including pain when compared to a CG (*n* = 1 study; *n* = 237 individuals affected with cancer; intervention duration: 18-week cognitive behavioral intervention with 10 contacts) from week 10 onwards [79]. 

### 3.10. Acupuncture/Acupressure (TCM)

Acupuncture (A) has been used in all forms of medicinal healing in Chinese medicine. A is based on the essential theory of harmonizing imbalances in the body [53]. It is assumed that A triggers pain-inhibiting and regulation-promoting mechanisms at the neuronal, vegetative, and hormonal levels via local and systemic points of action [42,53,63]. The concept of A from Traditional Chinese Medicine (TCM) is the regulation of the energy flow of the pathways by the insertion of needles into defined body parts. These are supposed to positively influence disturbances of the organism. Electric current between two A points is used in electro-A. Acupressure (AP) is a variation of A in which the A points are stimulated by finger pressure and is suitable for self-treatment [63].

The literature search yielded seven results related to CIPN [16,42,53,63,68,88,93] and five results related to cancer pain [64,95,98,100,102]. Among the studies were six [16,53,64,95,100,102] reviews, one four-arm RCT [88], one retrospective service evaluation [68], one prospective Phase 2 study [93], and three guidelines [42,63,98]. The quality of four studies was rated excellent [42,53,63,64] according to the CASP scheme, seven studies were rated good [16,88,93,95,98,100,102], and one was rated satisfactory [68]. Further details on the assessment of study quality according to CASP can be found in Supplementary S5 (Table S3).

In the studies described in one review, significant effects could be described in the application of EA in different outcomes. Regarding CIPN, EA was not superior to placebo (*n* = 13 RCTs; *n* = 1370 participants) [16]. In another review, it was confirmed that A has been frequently studied for cancer-related pain, but the data regarding alleviation of CIPN have not yet been convincing (*n* = 4 meta-analyses, 14 systematic reviews, 16 RCTs) [53]. One RCT investigated the use of electro-A for CIPN, but because the sample was small, no final results could indicate a clear improvement for CIPN (*n* = 1 study; *n* = 60 participants; intervention duration: three weeks) [88]. Improvement of CIPN symptoms was reported for EA (*n* = 1 study; *n* = 27; intervention duration: six to eight weeks) [93] and for A (*n* = 1 study; *n* = 18 participants; intervention duration: six weeks) [68]. According to one guideline, A can be considered for CIPN [63]. According to another guideline, there is insufficient evidence that EA help to reduce neuropathy in breast cancer patients [42]. Positive effects for A for joint pain in cancer survivors were described in one guideline [98]. Another review referred to a study on acupuncture by Lim et al. 2011; here, A was tolerated, but little to no effects could be found in terms of pain reduction (*n* = 11 studies; *n* = 1047 participants) [100].

Two further studies have described A for general pain, thus not differentiating in neuropathic pain [42,70,98]. AP can be used for nausea or tumor pain [63,64] but has not been described for CIPN. Two further reviews related to A state that little data exist [102], and evidence on A is sparse on CIPN [95]. In one review, A was found to be beneficial to reduce chemotherapy-induced nausea and vomiting (*n* = 37 systematic reviews) [64].

### 3.11. TENS/Scrambler Therapy

Transcutaneous electrical nerve stimulation (TENS) reduces the enhanced central excitability of nociceptive neurons and decreases release of the excitatory neurotransmitter glutamate in the spinal cord [65]. TENS is usually applied at the site of pain, stimulating large-diameter (A-β) afferent fibers, resulting in decreased transmission cell activity and subsequently decreased pain perception, according to the gate-control theory [119]. Scrambler therapy (ST) is an electro-analgesia therapy for the non-invasive treatment of chronic neuropathic and cancer pain. 

The literature search yielded three results related to CIPN [65,67,71] and four to cancer pain [66,98,100,102]. Within these studies, two systematic reviews [100,102], three four-arm RCTs [65,67,71] and two guidelines [66,98] were included. The quality of two studies was rated as excellent according to the CASP scheme [65,66], three studies as good [98,100,102], and two as satisfactory [67,71]. Further details on the assessment of study quality according to the CASP scheme can be found in Supplementary S5 (Table S3).

#### 3.11.1. TENS

A wireless, patient-controlled TENS unit significantly improved CIPN and associated outcomes (numeric rating scale of pain, tingling, numbness, and cramping) after patients’ completion of chemotherapy (*n* = 1 study; *n* = 26 patients with CIPN symptoms; intervention duration: 2–6 h per day stimulation for 6 weeks). 

Two systematic reviews assessed TENS. One of the included studies in an educational review on CIPN describes the physical effects resulting of TENS: increase of endorphin release and block noxious sensory impulses trough distraction, strongly supporting that physical therapists may administer TENS [102]. One of eleven studies in one systematic review [100] found TENS to have the potential to reduce cancer bone pain. One expert standard discussed TENS for pain management with the conclusion that there is currently not enough evidence of TENS efficacy for adults with neuropathic pain [66]. One clinical guideline on cancer survivorship recommended TENS as well as ST as non-pharmacological treatment options for general cancer pain, pointing out that oncologists might refer to such therapy options when addressing patients’ needs in consultations [98]. 

#### 3.11.2. Scrambler Therapy

Positive effects with regard to ST have been reported (*n* = 2 studies). Standardized ST significantly and clinically importantly reduced pain resulting of CIPN at 14 days as well as 1, 2, and 3 months (*n* = 1 study; *n* = 39 oncology patients; intervention duration: 45-min daily treatment for 10 consecutive days) [67]. Scrambler-treated patients experienced significantly greater improvement in pain, numbness, and tingling compared to TENS-treated patients (*n* = 1 study; *n* = 50 patients with CIPN; intervention duration: 30 min per day for 10–14 days) [71]. 

### 3.12. Conceptual Therapeutic Approach

In 6 out of the 75 studies, statements were made on the conceptual therapeutic approach [17,35,37,41,50,75]. These were as follows: use of analgesic, anti-inflammatory effects [17,75]; regulation of muscles, joints, tendons, and ligaments in the body [41]; promote blood circulation [35]; influence the energy of life [35,50]; improve or normalize nerve activity through mechanical stimuli [37]; and activation of TRP (transient receptor potential) ion channels [37].

### 3.13. Side Effects and/or Interactions

All reported therapies have been described as well tolerated if administered by trained healthcare staff; however, it should be noted that sporadically some side effects may occur. Patients receiving TENS have reported contact dermatitis, worsening or new paresthesia, or pain and cramping or tightness in the lower limps [65,71,100]. Contraindications to the use of TENS include gestational adherence and sensitive skin. In addition, the electrodes should not be placed near or over a pacemaker or implanted defibrillator, on the front of the neck and on the front of the chest [66]. Scrambler therapy has been reviewed as a safe and analgesic intervention for CIPN, and its use can be considered in clinical care [120,121,122]. Few adverse events have been reported for massage (M): for example, cerebrovascular accidents, hematoma, nerve damage, and various pain syndromes [53,69]. One study also reported respiratory infection and gastrointestinal bleed [51]. Additionally, acupuncture (A) is not entirely risk free, as rare cases of pneumothorax and infections have been reported [53,123]. Regarding Yoga (Y), only one study has reported adverse events in patients with back pain [52]. Otherwise, Y can be considered as a safe mind–body intervention and can in particular be recommended to patients for improving their quality of life as well as their mental health status [124]. Adverse events for cupping (C) are rare as well, but there have been three cases of fainting (vaso-vagal syncope) reported with wet C [55]. No recommendation can be made for Acetyl-l-carnitine (AlC), as this supplement may worsen CIPN [16,42,63,125].

## 4. Discussion

This two-phase approach with a systematic scoping review and a structured consensus process aimed to provide interprofessional healthcare teams with a comprehensive review and recommendations for clinical practice of the best available evidence on complementary treatments for the supportive management of CIPN. In total, 13 non-pharmacological interventions were identified. The evidence of the scoping review showed that patients with cancer may benefit from treatments that healthcare providers practice or recommend for patients like massage (M), reflexology (R), therapeutic touch (TT), rhythmical embrocations (REH), or aromatherapy (AT) or AT-M. One advantage of complementary treatments is that patients may practice, after a short briefing and education session, those therapies by themselves, like movement therapies (MT) ranging from cardiovascular exercises (CardE) to walking (W), as well as mind–body therapies (MBT) like meditation (Me), Yoga (Y), or relaxation (Rel) techniques. Interprofessional healthcare teams, however, should be aware that each potential intervention should be carefully considered regarding its external study evidence and its study quality, as well as the individual patients’ needs and preferences and their possibly prior experiences with complementary therapies.

The recommendations by the integrative oncology expert panel (Table 1 and Tables S1 and S5) demonstrated a highly consistent benefit in preventing or relieving CIPN symptoms patients with cancer can experience when using complementary treatments by nursing interventions in the form of phytotherapeutic (Phy) interventions, cryotherapy (CT), hydrotherapy (HTK), and tactile stimulation (TS). Additional supportive interventions are presented in Table S2 of the scoping review. Here, the authors aimed to match the expert recommendations from practice with external study evidence and wanted to highlight even more treatment possibilities. In total, we could include 16 studies with different study designs to demonstrate that there is incipient nascent evidence for different nursing interventions in the context of CIPN symptom management. The effects for henna application (HA), cryocompression (CC), frozen gloves (FG), cryotherapy (CT), classical massage (M), foot massage (FM), foot reflexology (FR), reflexology (R), and herbal medicines (Phy) and sensorimotor training (SM) are encouraging and have been presented as possible further complementary treatment options. Previous reviews [15,18] or clinical guidelines [42,63] have not been able to clearly point out such recommendations, probably due to their publication date or because they only considered RCTs, and non-RCTs were left out for a reason due to quality/risk of bias concerns. Thus, the authors think that the above mentioned non-pharmacological interventions are priority areas for future rigorous RCTs to confirm efficacy prior to being routinely recommended to patients. 

With the help of a scoping review methodology, the authors were able to also include studies with a non-RCT study design and pilot studies, as well as studies with a quasi-experimental design, case reports, or controlled studies, to point out a comprehensive map of treatments that are available and might help in individual patients. Of course, the presented therapy options cannot be applied with a “watering can principle” or a “one-size-fits-all” approach. According to the EbM approach, the choice of intervention is selected in consideration of the patient’s perspective and the individual clinical expertise of the health professionals [22]. For all presented complementary treatments, appropriate training is necessary, and a precise understanding of the interventions as well as the indications and contraindications is an important precondition before applying those interventions in everyday care. As a first step into the field of complementary therapies, interested healthcare professionals can find an overview of effective therapies here, but the second step needs to be a comprehensive training in such therapies to precisely get to know which patients can benefit from which complementary therapies in which situations.

### 4.1. Interprofessional Teamwork

Cancer treatment and care involves interprofessional and interdisciplinary teamwork and collaboration around and with the cancer patient (see Figure 3). Within comprehensive cancer care, it is even more important that healthcare professionals collaborate with each other to find together the most effective and best treatment strategy for the cancer patient and his/her family. Thus, with Table S2, the authors aimed to demonstrate other advances in complementary treatment options that can be conducted by other healthcare professionals like doctors, psychologists, nutrition therapists, midwives, pharmacists, or physiotherapists. If nurses get to know that their patients are interested in other complementary therapies—for instance, acupuncture (A), mind–body therapies (MBT), movement therapies (MT), TENS/scrambler therapy (ST), and nutritional therapy (NT)—to better cope with the CIPN symptoms, they might refer their patients to their healthcare professionals’ colleagues and vice versa. In total, Table S2 has 59 entries with references to external study evidence. All included studies involved different healthcare professionals (except nurses as they are considered in Table S1) and primarily focused on improving the outcome CIPN. In some cases, however, the authors also included studies if they focused on pain only [15,67,86], or included study participants other than patients with cancer [47,86,97]. The authors did this additional effort and described the concept of effect to demonstrate which complementary therapies were examined in similar clinical contexts with the according outcomes, so that those results could be used as basis in future research on CIPN.

### 4.2. Challenges of Categorizing Non-Pharmacological Therapies

In the thriving field of complementary therapies, it is highly relevant to differentiate between therapies that indeed have been studied in research and have demonstrated clinical effects compared to those therapies that have not been studied in research. The authors of the present study wanted to report proven treatment options, which might be considered in some patient cases, so that patient safety and well-being can be guaranteed. In addition, the framework of Kneipp has been in use for many years, especially in German-speaking countries, and has also become an established system in academic teaching of complementary therapies [126,127]. For collating and assembling the results from the expert symposium and the many study references, the authors found this classification system very useful, as every expert recommendation or study reference could be assigned to one of the 13 categories. Sometimes, there were overlaps of categories, for example in the case of Qi Gong (QG) or Tai Chi (TC), as those therapies originate of a Traditional Chinese Medicine (TCM) context. As these also can be applied detached from TCM, they were considered in two categories: “mind–body therapies (MBTs)” (classical natural therapies) AND “other TCM categories” (non-classical natural therapies). The authors would have done the same with Yoga (Y), and would have made a frequency count in two categories: “mind–body therapies (MBT)” AND “Ayurvedic medicine”, as this technique is a holistic practice system of its own (hence, it would count in the category “MBT”), but is also closely connected with Ayurvedic medicine and can be applied in Ayurvedic contexts too (hence, it would further count in the category “Ayurvedic medicine”). At this time point, however, there have been no further studies or recommendations published for CIPN management from Ayurvedic medicine, so these are just explanations in the subjunctive and a call to do further research in this field, as it is highly requested by patients [128,129].

In the current review, the systematic categorization of classical natural therapies according to Kneipp and the non-classical natural therapies were applied to better point out the effect concept of the categorized interventions. According to this established categorization, for instance, the authors of the current review would not have categorized Y, QG, or TC exclusively to MT, but rather to MBT (or non-classical natural therapies and Ayurvedic medicine in the case of Y or TCM in the case of QG and TC), as the effect concept is different compared to classical MT like AeT or RT. Future research on CIPN interventions might focus more on patient-reported outcomes measures (PROMs) and validated grading systems for neuropathy in the first instance, so that patient-oriented outcomes (PROs) can be measured and compared within and between the studies. Such data are also relevant for clinical consultations in which healthcare professionals can then better address the current needs and demands of their patients.

### 4.3. Further Integration of Non-Pharmacological Therapies in the Healthcare System

There is a high demand and interest in complementing conventional care by patients [13,130] and by providers [131,132]. It is urgent that the structures in the healthcare systems be adjusted and enhanced to meet patients’ needs and to guarantee patient safety. If patients are not informed and counselled by their healthcare team in the clinics or by their GPs, they consult other external providers who often do not have adequate education and take a lot of money out of the patient’s pocket [133]. Studies indicate that the majority of nurses have a positive attitude towards complementary therapies, but most of them feel unsure about how to talk about those therapies with their patients [134]. The topic of indication-based application of complementary therapies has not been anchored enough in medical and healthcare education, but this is highly needed, so that healthcare professionals are educated at an early stage on evidence-based complementary therapy options and can communicate this with their patients in an open, self-confident, and competent dialogue. Within this clinical practice guideline, the authors aim to inform and educate interested healthcare providers on the treatment possibilities for CIPN symptom management. We have presented the evidence for the seven most frequently mentioned complementary therapies: (1) manipulative therapies (2) phytotherapy, (3) movement therapies, (4) mind–body therapies, (5) acupuncture/acupressure, (6) rhythmical embrocations, and (7) TENS/scrambler therapy. Three of the five Kneipp’s pillars have been presented as the top treatment options, as there has been much evidence identified for those pillars. Interested healthcare professionals might start by engaging themselves with a basic training in the five pillars of classical natural therapies on which the naturopathic health concept according to Kneipp is based [126,127].

### 4.4. Directions for Future Research

The review does not only aim to inform and extend clinical practice, but also identifies important areas for future research. This includes in particular the important question if CIPN can be prevented. Currently, the majority of research focuses on treating CIPN, and some researchers are of the opinion that there are no effective preventive applications available for CIPN [76,132]. The current results, however, show recommendations for 20 prophylactic interventions (see Table 1) that can be investigated further in systematic research. There have also been some studies reported on Phy for preventive use [29,44]; however, such reports need to be considered with caution, as in non-Western countries, there is often a mixture of substances usually tested in studies and, thus, transferability is difficult [105]. More research on preventive measures is therefore highly recommended, mainly because the conventional therapies are not sufficiently satisfactory.

The consensus results (the preventive and complementary treatment options for CIPN) of the expert panel can be used in clinical practice and can also be used as a basis for designing future research in this area. The applied classification system of classical and non-classical natural therapies clearly illustrates that, in the context of CIPN management, there is much experiential evidence for nursing interventions (around EAP/AT/ToT/Phy) and much external evidence for MT or/and MBT. The latter therapy options have been broadly considered in research studies, and it is expected that future research of nursing interventions will follow a similar line, and that suitable research designs and contexts will be encouraged by appropriate requests for research proposals promoted by healthcare organizations and institutes. As long as there is not much external evidence of nursing studies nor clinical guidelines focusing on recommendations for action in this field, it is even more important to make the practical experience of the many years of experiential knowledge of oncology nurses visible and applicable to other clinics and nurses. Stolz et al. [20] reported on a systematic methodology combining both evidence types with which it is possible to generate clinical recommendations. Following this approach, the authors were able to comprehensively overview all possible complementary therapy options for CIPN management, thereby considering the field from an interprofessional perspective; thus, the authors could present in detail which therapies can be conducted by which healthcare professionals (Figure 3). Future research may be especially recommended for cryotherapy interventions, however, only for cryocompression and continuous-flow hypothermia [18,40]. In contrast to the high drop-out rates up to 50% for cryotherapy with frozen gloves [19], cryocompression and continuous-flow hypothermia [18,40] were safe and well-tolerated interventions to prevent CIPN and should be investigated in a larger trial. 

For future intervention research, the authors strongly recommend including a qualitative–quantitative process analysis running parallel to the main outcome study, so that it is possible to (1) include the perspective of the patients and the health professionals and (2) consider context and individual aspects that cannot be measured with quantitative data [135]. Another important aspect for future supportive care research, especially in the area of CIPN, is to include more patient-reported outcomes measures (PROMs, which are experienced by patients in everyday life) and patient-reported experience measures (PREMs, like patient satisfaction with conventional and complementary treatment), so that patient care interventions can be assessed in a validated and reliable way.

### 4.5. Strengths and Limitations

The present study has some limitations. First, this review mainly considered published English, French, and German language literature about adult patients with cancer. The results could have been even more comprehensive if we included opinions and expertise from professionals in the languages and databases of other interesting cultures like Asia, the Middle East, South America, and Africa, with a great tradition in naturopathic tradition. However, this review did include studies conducted in non-English- and non-German-speaking countries, such as China, Finland, France, Iran, Italy, Netherlands, Singapore, Republic of Korea, Sweden, Taiwan, and Turkey. Second, even though the authors oriented on the guidelines for conducting a scoping review to be able to include a wider range and consider a variability of different studies, the review did not include a search of the many written and electronic sources of information for patients or for health professionals. In total, the authors included 17 expert recommendations, 70 studies, and 5 clinical guidelines relevant for the treatment of polyneuropathies resulting from chemotherapy treatment, which is a considerable data pool, but it could have been more extensive if the authors were able to consider other contexts and sources too. With the help of a scoping review study design [26,27,136], the authors aimed to also include studies with a non-randomized study design, pilot studies, and qualitative studies focusing on the patient perspective and their experiences. However, as the results show, the authors included 42 quantitative studies with different designs, but the search only yielded 3 mixed-methods studies (controlled studies with a qualitative component). The authors would have expected more qualitative results, especially in studies reporting about innovative complementary medicine interventions. Furthermore, even though the core research team has many years of experience in academic work as well as in patient care in Western countries, it could have been affected by reporting bias [137], with the tendency of researchers to submit positive, but not negative results for publication. 

Despite these limitations, this is, to the best of the authors’ knowledge, the first comprehensive review with clinical recommendations that reports about supportive treatment options for patients with cancer affected by polyneuropathies. In addition, by examining not only the nursing interventions, but also the interventions conducted by medical doctors, psychologists, physiotherapists, and nutritional therapists, a stronger invocation for considering applying such complementary therapies in routine care and treatment is provided. The authors decided to appraise the quality of the studies, as it is common with systematic reviews and increasingly with scoping reviews, by applying CASP [33] to have a better insight into the quality of the included studies and to know if some studies need to be excluded. The CASP appraisal form was, however, not always suitable for the different studies considered. As there were no appraisal forms available, for example for case reports or retrospective studies, those were appraised with the CASP-RCT form, even though not all questions (in particular questions 4–7) were applicable. The rating would therefore have been different with other appraisal tools.

## 5. Conclusions

The best available evidence of non-pharmacological treatments for CIPN management in patients with cancer are summarized herein. Based on the literature and the long-term practical experience of the involved experts, the interventions shown here have high potential to be widely implemented into routine care. For complementary methods for CIPN to be implemented into safe and evidence-based practice, healthcare providers would need to expand clinical practice with additional training. Patients would need more information that empowers them to actively ask their healthcare team for these treatments more often. To support this, future research in symptom care and treatment would be useful to make the expert knowledge “tangible”. Future prospective trials are also needed to validate the efficacy and safety of the here presented non-pharmacological interventions in individuals affected with cancer, so targeted interventions can be developed and implemented to treat and prevent the symptoms of complex CIPN.

## Data Availability

Not applicable.

## Supplementary Materials

The following are available online at https://www.mdpi.com/article/10.3390/medsci11010015/s1, Supplementary S1: “PRISMA-ScR checklist”, Supplementary S2: “Search strategy”, Supplementary S3: “Table S1. Characteristics of selected studies included in the scoping review (nursing interventions for CIPN)”, Supplementary S4: “Table S2. “Characteristics of other relevant studies for treating pain with complementary therapies in cancer patients of other patient populations”, Supplementary S5: “Table S3. Critical appraisal”, Supplementary S6: “Table S4. Expert judgements of nursing procedures for treating CIPN based on consensus process”.

## References

[1] Hou S., Huh B., Kim H.K., Kim K.-H., Abdi S. (2018). Treatment of Chemotherapy-Induced Peripheral Neuropathy: Systematic Review and Recommendations. Pain Physician.

[2] Colvin L.A. (2019). Chemotherapy-induced peripheral neuropathy: Where are we now?. Pain.

[3] Areti A., Yerra V.G., Komirishetty P., Kumar A. (2016). Potential Therapeutic Benefits of Maintaining Mitochondrial Health in Peripheral Neuropathies. Curr. Neuropharmacol..

[4] Kolb N.A., Smith A.G., Singleton J.R., Beck S.L., Stoddard G.J., Brown S., Mooney K. (2016). The Association of Chemotherapy-Induced Peripheral Neuropathy Symptoms and the Risk of Falling. JAMA Neurol..

[5] Molassiotis A., Cheng H.L., Lopez V., Au J.S.K., Chan A., Bandla A., Leung K.T., Li Y.C., Wong K.H., Suen L.K.P. (2019). Are we mis-estimating chemotherapy-induced peripheral neuropathy? Analysis of assessment methodologies from a prospective, multinational, longitudinal cohort study of patients receiving neurotoxic chemotherapy. BMC Cancer.

[6] Staff N.P., Grisold A., Grisold W., Windebank A.J. (2017). Chemotherapy-induced peripheral neuropathy: A current review. Ann. Neurol..

[7] Bandos H., Melnikow J., Rivera D.R., Swain S.M., Sturtz K., Fehrenbacher L., Wade J.L., Brufsky A.M., Julian T.B., Margolese R.G. (2018). Long-term Peripheral Neuropathy in Breast Cancer Patients Treated with Adjuvant Chemotherapy: NRG Oncology/NSABP B-30. J. Natl. Cancer Inst..

[8] Zajączkowska R., Kocot-Kępska M., Leppert W., Wrzosek A., Mika J., Wordliczek J. (2019). Mechanisms of Chemotherapy-Induced Peripheral Neuropathy. Int. J. Mol. Sci..

[9] Smith E.M.L., Pang H., Cirrincione C., Fleishman S., Paskett E.D., Ahles T., Bressler L.R., Fadul C.E., Knox C., Le-Lindqwister N. (2013). Effect of duloxetine on pain, function, and quality of life among patients with chemotherapy-induced painful peripheral neuropathy: A randomized clinical trial. JAMA.

[10] Hershman D.L., Lacchetti C., Dworkin R.H., Smith E.M.L., Bleeker J., Cavaletti G., Chauhan C., Gavin P., Lavino A., Lustberg M.B. (2014). Prevention and management of chemotherapy-induced peripheral neuropathy in survivors of adult cancers: American Society of Clinical Oncology clinical practice guideline. J. Clin. Oncol..

[11] Boon H.S., Olatunde F., Zick S.M. (2007). Trends in complementary/alternative medicine use by breast cancer survivors: Comparing survey data from 1998 and 2005. BMC Womens Health.

[12] Keene M.R., Heslop I.M., Sabesan S.S., Glass B.D. (2019). Complementary and alternative medicine use in cancer: A systematic review. Complement. Ther. Clin. Pract..

[13] Buckner C.A., Lafrenie R., Dénommée J.A., Caswell J.M., Want D.A. (2018). Complementary and alternative medicine use in patients before and after a cancer diagnosis. Curr. Oncol..

[14] Teoh D., Smith T.J., Song M., Spirtos N.M. (2018). Care After Chemotherapy: Peripheral Neuropathy, Cannabis for Symptom Control, and Mindfulness. Am. Soc. Clin. Oncol. Educ. Book.

[15] Bardia A., Barton D.L., Prokop L.J., Bauer B.A., Moynihan T.J. (2006). Efficacy of complementary and alternative medicine therapies in relieving cancer pain: A systematic review. J. Clin. Oncol..

[16] Brami C., Bao T., Deng G. (2016). Natural products and complementary therapies for chemotherapy-induced peripheral neuropathy: A systematic review. Crit. Rev. Oncol. Hematol..

[17] Arslan S., Bahceli P.Z., Ilik Y., Artaç M. (2020). The preliminary effects of henna on chemotherapy-induced peripheral neuropathy in women receiving oxaliplatin-based treatment: A parallel-group, randomized, controlled pilot trial. Eur. J. Oncol. Nurs..

[18] Bandla A., Tan S., Kumarakulasinghe N.B., Huang Y., Ang S., Magarajah G., Hairom Z., Lim J.S.J., Wong A., Chan G. (2020). Safety and tolerability of cryocompression as a method of enhanced limb hypothermia to reduce taxane-induced peripheral neuropathy. Support. Care Cancer.

[19] Griffiths C., Kwon N., Beaumont J.L., Paice J.A. (2018). Cold therapy to prevent paclitaxel-induced peripheral neuropathy. Support. Care Cancer.

[20] Stolz R., Klafke N., Kröger B., Boltenhagen U., Kaltenbach A., Heine R., Idler C., Layer M., Kohler S., Winkler M. (2021). Creating evidence for naturopathic nursing interventions in oncology—A systematic approach. Z Evid. Fortbild. Qual. Gesundhwes..

[21] Steinmann D., Babadağ Savaş B., Felber S., Joy S., Mertens I., Cramer H., Paul A., Layer M., Klafke N., Stolz R. (2021). Nursing Procedures for the Prevention and Treatment of Mucositis Induced by Cancer Therapies: Clinical Practice Guideline Based on an Interdisciplinary Consensus Process and a Systematic Literature Search. Integr. Cancer Ther..

[22] Sackett D.L., Rosenberg W., Gray J.A.M., Haynes R.B., Richardson W.S. (1996). Evidence based medicine: What it is and what it isn’t. BMJ.

[23] CEBM Oxford Centre for Evidence-Based Medicine: Levels of Evidence (March 2009). https://www.cebm.ox.ac.uk/resources/levels-of-evidence/oxford-centre-for-evidence-based-medicine-levels-of-evidence-march-2009.

[24] Assaf C., Booken N., Dippel E., Guenova E., Jonak C., Klemke C.D., Nicolay J.P., Schlaak M., Wobser M., Trautinger F. (2022). Chlormethin-Gel zur Behandlung der Mycosis fungoides: Ein Expertenkonsens aus Deutschland, Österreich und der Schweiz (DACH-Region) zum Therapiemanagement. J. Dtsch. Dermatol. Ges..

[25] Tricco A.C., Lillie E., Zarin W., O’Brien K.K., Colquhoun H., Levac D., Moher D., Peters M.D., Horsley T., Weeks L. (2018). PRISMA Extension for Scoping Reviews (PRISMA-ScR): Checklist and Explanation. Ann. Intern. Med..

[26] Arksey H., O’Malley L. (2005). Scoping studies: Towards a methodological framework. Int. J. Soc. Res. Methodol..

[27] Levac D., Colquhoun H., O’Brien K.K. (2010). Scoping studies: Advancing the methodology. Implement. Sci..

[28] Peters M., Godfrey C., McInerney P., Munn Z., Tricco A.C., Khalil H., Aromataris E., Munn Z. (2020). Chapter 11: Scoping Reviews (2020 Version). JBI Manual for Evidence Synthesis.

[29] National Center for Complementary and Integrative Health Complementary, Alternative, or Integrative Health: What’s in a Name? 2021. https://www.nccih.nih.gov/health/complementary-alternative-or-integrative-health-whats-in-a-name.

[30] Witt C.M., Balneaves L.G., Cardoso M.J., Cohen L., Greenlee H., Johnstone P., Kücük ., Mailman J., Mao J.J. (2017). A Comprehensive Definition for Integrative Oncology. J. Natl. Cancer Inst. Monogr..

[31] Stolz R., Klafke N. (2020). Complementary Nursing Procedures to Treat Polyneuropathy in Cancer Patients Undergoing Chemotherapy. https://www.crd.york.ac.uk/prospero/display_record.php?ID=CRD42020165851.

[32] Ouzzani M., Hammady H., Fedorowicz Z., Elmagarmid A. (2016). Rayyan-a web and mobile app for systematic reviews. Syst. Rev..

[33] Critical Appraisal Skills Programme (2019). CASP (Randomised Controlled Trials Checklist, Systematic Review Checklist, Qualitative Studies Checklist, Cohort Study Checklist). https://casp-uk.net/casp-tools-checklists/.

[34] AWMF (2022). AWMF-Regelwerk Leitlinien: Strukturierte Konsensfindung. https://www.awmf.org/leitlinien/awmf-regelwerk/leitlinien-entwicklung/awmf-regelwerk-03-leitlinienentwicklung/ll-entwicklung-strukturierte-konsensfindung.html.

[35] Li Z., Jin H., Yan Q., Sun L., Wasan H.S., Shen M., Ruan S. (2019). The Method of Activating Blood and Dredging Collaterals for Reducing Chemotherapy-Induced Peripheral Neuropathy: A Systematic Review and Meta-Analysis. Evid. Based Complement. Alternat. Med..

[36] Noh H., Yoon S., Park B. (2018). A Systematic Review of Herbal Medicine for Chemotherapy Induced Peripheral Neuropathy. Evid. Based Complement. Alternat. Med..

[37] AWMF Leitlinienprogramm Onkologie der Arbeitsgemeinschaft der Wissenschaftlichen Medizinischen Fachgesellschaften e.V. (AWMF), Deutschen Krebsgesellschaft e.V. (DKG) und Deutschen Krebshilfe (DKH). Supportive Therapie bei onkologischen PatientInnen—Langversion 1.3. 2020. https://register.awmf.org/assets/guidelines/032-054OLl_S3_Supportiv_2020-07-abgelaufen.pdf.

[38] Andersen Hammond E., Pitz M., Steinfeld K., Lambert P., Shay B. (2020). An Exploratory Randomized Trial of Physical Therapy for the Treatment of Chemotherapy-Induced Peripheral Neuropathy. Neurorehabilit. Neural Repair.

[39] Beijers A., Bonhof C., Mols F., Ophorst J., de Vos-Geelen J., Jacobs E., van de Poll-Franse L., Vreugdenhil G. (2020). Multicenter randomized controlled trial to evaluate the efficacy and tolerability of frozen gloves for the prevention of chemotherapy-induced peripheral neuropathy. Ann. Oncol..

[40] Sundar R., Bandla A., Tan S.S.H., Liao L.-D., Kumarakulasinghe N.B., Jeyasekharan A.D., Ow S.G.W., Ho J., Tan D.S.P., Lim J.S.J. (2017). Limb hypothermia for preventing paclitaxel-induced peripheral neuropathy in breast cancer patients: A pilot study. Front. Oncol..

[41] Izgu N., Metin Z.G., Karadas C., Ozdemir L., Çetin N., Demirci U. (2019). Prevention of chemotherapy-induced peripheral neuropathy with classical massage in breast cancer patients receiving paclitaxel: An assessor-blinded randomized controlled trial. Eur. J. Oncol. Nurs..

[42] Greenlee H., DuPont-Reyes M.J., Rn L.G.B., Carlson L.E., Cohen M.R., Deng G., Johnson J.A., Mumber M., Seely D., Zick S.M. (2017). Clinical practice guidelines on the evidence-based use of integrative therapies during and after breast cancer treatment. CA A Cancer J. Clin..

[43] Kotani H., Terada M., Mori M., Horisawa N., Sugino K., Kataoka A., Adachi Y., Gondou N., Yoshimura A., Hattori M. (2021). Compression therapy using surgical gloves does not prevent paclitaxel-induced peripheral neuropathy: Results from a double-blind phase 2 trial. BMC Cancer.

[44] Volger E., Brinkhaus B., Volger E., Brinkhaus B. (2017). Einteilung der Naturheilverfahren. Kursbuch Naturheilverfahren für die Ärztliche Weiterbildung.

[45] Dikmen H.A., Terzioglu F. (2019). Effects of reflexology and progressive muscle relaxation on pain, fatigue, and quality of life during chemotherapy in gynecologic cancer patients. Pain Manag. Nurs..

[46] Dyer J., Thomas K., Sandsund C., Shaw C. (2013). Is reflexology as effective as aromatherapy massage for symptom relief in an adult outpatient oncology population?. Complement. Ther. Clin. Pract..

[47] Gok Metin Z., Arikan Donmez A., Izgu N., Ozdemir L., Arslan I.E. (2017). Aromatherapy massage for neuropathic pain and quality of life in diabetic patients. J. Nurs. Scholarsh..

[48] Kleckner I.R., Kamen C., Gewandter J.S., Mohile N.A., Heckler C.E., Culakova E., Fung C., Janelsins M.C., Asare M., Lin P.-J. (2018). Effects of exercise during chemotherapy on chemotherapy-induced peripheral neuropathy: A multicenter, randomized controlled trial. Support. Care Cancer.

[49] Kutner J.S., Smith M.C., Corbin L., Hemphill L., Benton K., Mellis B.K., Beaty B., Felton S., Yamashita T.E., Bryant L.L. (2008). Massage therapy versus simple touch to improve pain and mood in patients with advanced cancer [with consumer summary]. Ann. Intern. Med..

[50] Noh G.O., Park K.S. (2019). Effects of aroma self-foot reflexology on peripheral neuropathy, peripheral skin temperature, anxiety, and depression in gynaecologic cancer patients undergoing chemotherapy: A randomised controlled trial. Eur. J. Oncol. Nurs..

[51] Boyd C., Crawford C., Paat C.F., Price A., Xenakis L., Zhang W., Group E.f.M.T.W. (2016). The impact of massage therapy on function in pain populations—A systematic review and meta-analysis of randomized controlled trials: Part II, cancer pain populations. Pain Med..

[52] Buffart L.M., Van Uffelen J.G.Z., I Riphagen I., Brug J., Van Mechelen W., Brown W.J., Chinapaw M.J.M. (2012). Physical and psychosocial benefits of yoga in cancer patients and survivors, a systematic review and meta-analysis of randomized controlled trials. BMC Cancer.

[53] Deng G.E., Rausch S.M., Jones L.W., Gulati A., Kumar N.B., Greenlee H., Pietanza M.C., Cassileth B.R. (2013). Complementary therapies and integrative medicine in lung cancer: Diagnosis and management of lung cancer, 3rd ed: American College of Chest Physicians evidence-based clinical practice guidelines. Chest.

[54] Kanzawa-Lee G.A., Larson J.L., Resnicow K., Smith E.M.L. (2020). Exercise Effects on Chemotherapy-Induced Peripheral Neuropathy: A Comprehensive Integrative Review. Cancer Nurs..

[55] Kim J.-I., Lee M.S., Lee D.-H., Boddy K., Ernst E. (2011). Cupping for treating pain: A systematic review. Evid. Based Complement. Altern. Med..

[56] Lee S.-H., Kim J.-Y., Jong-Yeop K., Kim S., Lim S. (2015). Meta-analysis of massage therapy on cancer pain. Integr. Cancer Ther..

[57] Mishra S.I., Scherer R.W., Snyder C., Geigle P.M., Berlanstein D., Topaloglu O. (2012). Exercise interventions on health-related quality of life for cancer survivors (Cochrane review) [with consumer summary]. Cochrane Database Syst. Rev..

[58] Pan Y.Q., Yang K.H., Wang Y.L., Zhang L.P., Liang H.Q. (2013). Massage interventions and treatment-related side effects of breast cancer: A systematic review and meta-analysis. Int. J. Clin. Oncol..

[59] Stier-Jarmer M., Throner V., Kirschneck M., Frisch D., Schuh A. (2021). Effekte der Kneipp-Therapie: Ein systematischer Review der aktuellen wissenschaftlichen Erkenntnisse (2000–2019). Complement. Med. Res..

[60] Streckmann F., Zopf E.M., Lehmann H.C., May K., Rizza J., Zimmer P., Gollhofer A., Bloch W., Baumann F.T. (2014). Exercise intervention studies in patients with peripheral neuropathy: A systematic review. Sports Med..

[61] Van Vu D., Molassiotis A., Ching S.S.Y., Le T.T. (2017). Effects of Qigong on symptom management in cancer patients: A systematic review. Complement. Ther. Clin. Pract..

[62] Wayne P.M., Lee M., Novakowski J., Osypiuk K., Ligibel J., Carlson L., Song R. (2018). Tai Chi and Qigong for cancer-related symptoms and quality of life: A systematic review and meta-analysis. J. Cancer Surviv..

[63] AWMF Leitlinienprogramm Onkologie (Deutsche Krebsgesellschaft, Deutsche Krebshilfe, AWMF): Komplementärmedizin in der Behandlung von onkologischen PatientInnen, Langversion 1.1. https://www.leitlinienprogramm-onkologie.de/fileadmin/user_upload/Downloads/Leitlinien/Komplement%C3%A4r/Version_1/LL_Komplement%C3%A4r_Langversion_1.0.pdf.

[64] Möller U.O., Beck I., Rydén L., Malmström M. (2019). A comprehensive approach to rehabilitation interventions following breast cancer treatment—A systematic review of systematic reviews. BMC Cancer.

[65] Gewandter J.S., Chaudari J., Ibegbu C., Kitt R., Serventi J., Burke J., Culakova E., Kolb N., Sluka K.A., Tejani M.A. (2019). Wireless transcutaneous electrical nerve stimulation device for chemotherapy-induced peripheral neuropathy: An open-label feasibility study. Support. Care Cancer.

[66] Deutsches Netzwerk für Qualitätsentwicklung in der Pflege (DNQP) (2020). Expertenstandard Schmerzmanagement in der Pflege. Schriftenreihe des Deutschen Netzwerks für Qualitätsentwicklung in der Pflege.

[67] Coyne P.J., Wan W., Dodson P., Swainey C., Smith T.J. (2013). A Trial of Scrambler Therapy in the Treatment of Cancer Pain Syndromes and Chronic Chemotherapy-Induced Peripheral Neuropathy. J. Pain Palliat. Care Pharmacother..

[68] Donald G.K., Tobin I., Stringer J. (2011). Evaluation of acupuncture in the management of chemotherapy-induced peripheral neuropathy. Acupunct. Med..

[69] Fernandes J., Kumar S. (2016). Effect of lower limb closed kinematic chain exercises on balance in patients with chemotherapy-induced peripheral neuropathy: A pilot study. Int. J. Rehabil. Res..

[70] Kwekkeboom K.L. (2001). Pain management strategies used by patients with breast and gynecologic cancer with postoperative pain. Cancer Nurs..

[71] Loprinzi C., Le-Rademacher J.G., Majithia N., McMurray R.P., O’Neill C.R., Bendel M.A., Beutler A., Lachance D.H., Cheville A., Strick D.M. (2020). Scrambler therapy for chemotherapy neuropathy: A randomized phase II pilot trial. Support. Care Cancer.

[72] Ernst E. (2009). Is reflexology an effective intervention? A systematic review of randomised controlled trials. Med. J. Aust..

[73] Telles S., Sayal N., Nacht C., Chopra A., Patel K., Wnuk A., Dalvi P., Bhatia K., Miranpuri G., Anand A. (2019). Yoga: Can it be integrated with treatment of neuropathic pain?. Ann. Neurosci..

[74] Cunningham J.E., Kelechi T., Sterba K., Barthelemy N., Falkowski P., Chin S.H. (2011). Case report of a patient with chemotherapy-induced peripheral neuropathy treated with manual therapy (massage). Support. Care Cancer.

[75] Rostami N., Mosavat S.H., Heydarirad G., Arbab Tafti R., Heydari M. (2019). Efficacy of topical *Citrullus colocynthis* (bitter apple) extract oil in chemotherapy-induced peripheral neuropathy: A pilot double-blind randomized placebo-controlled clinical trial. Phytother. Res..

[76] Sarısoy P., Ovayolu O. (2020). The Effect of Foot Massage on Peripheral Neuropathy-Related Pain and Sleep Quality in Patients with Non-Hodgkin’s Lymphoma. Holist. Nurs. Pract..

[77] Stephenson N., Dalton J.A., Carlson J. (2003). The effect of foot reflexology on pain in patients with metastatic cancer. Appl. Nurs. Res..

[78] Wyatt G., Sikorskii A., Tesnjak I., Frambes D., Holmstrom A., Luo Z., Victorson D., Tamkus D. (2017). A randomized clinical trial of caregiver-delivered reflexology for symptom management during breast cancer treatment. J. Pain Symptom Manag..

[79] Doorenbos A., Given B., Given C., Verbitsky N., Cimprich B., McCorkle R. (2005). Reducing symptom limitations: A cognitive behavioral intervention randomized trial. Psychooncology.

[80] Galantino M.L., Tiger R., Brooks J., Jang S., Wilson K. (2019). Impact of somatic yoga and meditation on fall risk, function, and quality of life for chemotherapy-induced peripheral neuropathy syndrome in cancer survivors. Integr. Cancer Ther..

[81] Giasson M., Bouchard L. (1998). Effect of therapeutic touch on the well-being of persons with terminal cancer. J. Holist. Nurs..

[82] Koch B. (2015). Evaluation of Efficacy of Home-Based Kneipp Hydrotherapy in Patients with Polyneuropathic Compaints of the Lower Extremities.

[83] Listing M., Reißhauer A., Krohn M., Voigt B., Tjahono G., Becker J., Klapp B.F., Rauchfuß M. (2009). Massage therapy reduces physical discomfort and improves mood disturbances in women with breast cancer. Psychooncology.

[84] McCrary J.M., Goldstein D., Sandler C.X., Barry B.K., Marthick M., Timmins H.C., Li T., Horvath L., Grimison P., Park S.B. (2019). Exercise-based rehabilitation for cancer survivors with chemotherapy-induced peripheral neuropathy. Support. Care Cancer.

[85] Moore S., Corner J., Haviland J., Wells M., Salmon E., Normand C., Brada M., Smith I. (2002). Nurse led follow up and conventional medical follow up in management of patients with lung cancer: Randomised trial. BMJ.

[86] Ostermann T., Blaser G., Bertram M., Michalsen A., Matthiessen P.F., Kraft K. (2008). Effects of rhythmic embrocation therapy with solum oil in chronic pain patients: A prospective observational study. Clin. J. Pain.

[87] Post-White J., Kinney M.E., Savik K., Gau J.B., Wilcox C., Lerner I. (2003). Therapeutic massage and healing touch improve symptoms in cancer. Integr. Cancer Ther..

[88] Rostock M., Jaroslawski K., Guethlin C., Ludtke R., Schröder S., Bartsch H.H. (2013). Chemotherapy-Induced Peripheral Neuropathy in Cancer Patients: A Four-Arm Randomized Trial on the Effectiveness of Electroacupuncture. Evid. Based Complement. Altern. Med..

[89] Schönsteiner S.S., Bauder Mißbach H., Benner A., Mack S., Hamel T., Orth M., Landwehrmeyer B., Süßmuth S.D., Geitner C., Mayer-Steinacker R. (2017). A randomized exploratory phase 2 study in patients with chemotherapy-related peripheral neuropathy evaluating whole-body vibration training as adjunct to an integrated program including massage, passive mobilization and physical exercises. Exp. Hematol. Oncol..

[90] Schwenk M., Grewal G.S., Holloway D., Muchna A., Garland L., Najafi B. (2016). Interactive Sensor-Based Balance Training in Older Cancer Patients with Chemotherapy-Induced Peripheral Neuropathy: A Randomized Controlled Trial. Gerontology.

[91] Uehleke B., Wöhling H., Stange R. (2008). A prospective “Study by Correspondance” on the effects of Kneipp hydrotherapy in patients with complaints due to peripheral neuropathy. Schweiz. Z. Ganzheitsmed..

[92] Nagarathna R., Rekha M., Vanitha N., Kodaganuru G., Srinath B., Vishweshwara M., Madhavi Y., Basavalingaiah S.A., Bilimagga R.S., Rao N. (2009). Effects of yoga on symptom management in breast cancer patients: A randomized controlled trial. Int. J. Yoga.

[93] Wong R., Major P., Sagar S. (2016). Phase 2 Study of Acupuncture-Like Transcutaneous Nerve Stimulation for Chemotherapy-Induced Peripheral Neuropathy. Integr. Cancer Ther..

[94] Zimmer P., Trebing S., Timmers-Trebing U., Schenk A., Paust R., Bloch W., Rudolph R., Streckmann F., Baumann F.T. (2018). Eight-week, multimodal exercise counteracts a progress of chemotherapy-induced peripheral neuropathy and improves balance and strength in metastasized colorectal cancer patients: A randomized controlled trial. Support. Care Cancer.

[95] Dy S.M. (2010). Evidence-based approaches to pain in advanced cancer. Cancer J..

[96] Tofthagen C., Visovsky C., Berry D.L. (2012). Strength and Balance Training for Adults with Peripheral Neuropathy and High Risk of Fall: Current Evidence and Implications for Future Research. Oncol. Nurs. Forum.

[97] Verhulst A.L., Savelberg H.H., Vreugdenhil G., Mischi M., Schep G. (2015). Whole-Body Vibration as a Modality for the Rehabilitation of Peripheral Neuropathies: Implications for Cancer Survivors Suffering from Chemotherapy-Induced Peripheral Neuropathy. Oncol. Rev..

[98] Sanft T., Denlinger C.S., Armenian S., Baker K.S., Broderick G., Demark-Wahnefried W., Friedman D.L., Goldman M., Hudson M., Khakpour N. (2019). NCCN Guidelines Insights: Survivorship, Version 2.2019: Featured Updates to the NCCN Guidelines. J. Natl. Compr. Cancer Netw..

[99] Kim J.-I., Lee M.S., Kang J.W., Choi D.Y., Ernst E. (2010). Reflexology for the symptomatic treatment of breast cancer: A systematic review. Integr. Cancer Ther..

[100] Hökkä M., Kaakinen P., Pölkki T. (2014). A systematic review: Non-pharmacological interventions in treating pain in patients with advanced cancer. J. Adv. Nurs..

[101] Izgu N., Ozdemir L., Bugdayci F. (2019). Effect of Aromatherapy Massage on Chemotherapy-Induced Peripheral Neuropathic Pain and Fatigue in Patients Receiving Oxaliplatin: An Open Label Quasi-Randomized Controlled Pilot Study. Cancer Nurs..

[102] Armstrong T., Almadrones L., Gilbert M.R. (2005). Chemotherapy-induced peripheral neuropathy. Oncol. Nurs. Forum.

[103] Jane S.W., Wilkie D.J., Gallucci B.B., Beaton R.D. (2008). Systematic review of massage intervention for adult patients with cancer: A methodological perspective. Cancer Nurs..

[104] Aghabati N., Mohammadi E., Pour Esmaiel Z. (2010). The effect of therapeutic touch on pain and fatigue of cancer patients undergoing chemotherapy. Evid. Based Complement. Altern. Med..

[105] Grealish L., Lomasney A., Whiteman B.J.C.N. (2000). Foot massage: A nursing intervention to modify the distressing symptoms of pain and nausea in patients hospitalized with cancer. Cancer Nurs..

[106] Long H.A., French D.P., Brooks J.M. (2020). Optimising the value of the critical appraisal skills programme (CASP) tool for quality appraisal in qualitative evidence synthesis. Res. Methods Med. Health Sci..

[107] Holey E.A., Cook E.M. (2012). Evidence-Based Therapeutic Massage E-Book: A Practical Guide for Therapists.

[108] Layer M. (2014). Praxishandbuch Rhythmische Einreibungen nach Wegman/Hauschka.

[109] Frank L.S., Frank J.L., March D., Makari-Judson G., Barham R.B., Mertens W.C. (2007). Does therapeutic touch ease the discomfort or distress of patients undergoing stereotactic core breast biopsy? A randomized clinical trial. Pain Med..

[110] Samarel N., Fawcett J., Davis M.M., Ryan F.M. (1998). Effects of dialogue and therapeutic touch on preoperative and postoperative experiences of breast cancer surgery: An exploratory study. Oncol. Nurs. Forum.

[111] Tabatabaee A., Tafreshi M., Rassouli M., Aledavood S., Majd H., Farahmand A. (2016). Effect of therapeutic touch in patients with cancer: A literature review. Med. Arch..

[112] Büntzel J., Micke O., Büntzel J. (2021). How to transfer traditional knowledge about medicinal herbs? or TCM plants: A black box for modern oncologists. J Cancer Res. Clin. Oncol..

[113] Steflitsch W., Wolz D., Buchbauer G., Stadelmann I. (2021). Aromatherapie in Wissenschaft und Praxis.

[114] Maßberg D., Hatt H. (2018). Human Olfactory Receptors: Novel Cellular Functions Outside of the Nose. Physiol. Rev..

[115] Kontaris I., East B.S., Wilson D.A. (2020). Behavioral and Neurobiological Convergence of Odor, Mood and Emotion: A Review. Front. Behav. Neurosci..

[116] (2017). National Center for Complementary and Integrative Health. https://www.nccih.nih.gov/health/mind-and-body-practices.

[117] Danon N., Al-Gobari M., Burnand B., Rodondi P. (2022). Are mind-body therapies effective for relieving cancer-related pain in adults? A systematic review and meta-analysis. Psychooncology.

[118] Carlson L.E., Zelinski E., Toivonen K., Flynn M., Qureshi M., Piedalue K.-A., Grant R. (2017). Mind-body therapies in cancer: What is the latest evidence?. Curr. Oncol. Rep..

[119] Siemens W., Boehlke C., Bennett M.I., Offner K., Becker G., Gaertner J. (2020). Transcutaneous electrical nerve stimulation for advanced cancer pain inpatients in specialist palliative care-a blinded, randomized, sham-controlled pilot cross-over trial. Support Care Cancer.

[120] Lesenskyj A.M.C., Cruciani R. (2017). Optimizing Neuropathic Pain Relief with Scrambler Therapy. Pract. Pain Manag..

[121] Tomasello C., Pinto R.M., Mennini C., Conicella E., Stoppa F., Raucci U. (2018). Scrambler therapy efficacy and safety for neuropathic pain correlated with chemotherapy-induced peripheral neuropathy in adolescents: A preliminary study. Pediatr. Blood Cancer.

[122] Karri J., Marathe A., Smith T.J., Wang E.J. (2022). The Use of Scrambler Therapy in Treating Chronic Pain Syndromes: A Systematic Review. Neuromodulation.

[123] Höxtermann M.D., Haller H., Aboudamaah S., Bachemir A., Dobos G., Cramer H., Voiss P. (2022). Safety of acupuncture in oncology: A systematic review and meta-analysis of randomized controlled trials. Cancer.

[124] Cramer H., Lauche R., Klose P., Lange S., Langhorst J., Dobos G.J. (2017). Yoga for improving health-related quality of life, mental health and cancer-related symptoms in women diagnosed with breast cancer. Cochrane Database Syst. Rev..

[125] Loprinzi C.L., Lacchetti C., Bleeker J., Cavaletti G., Chauhan C., Hertz D.L., Kelley M.R., Lavino A., Lustberg M.B., Paice J.A. (2020). Prevention and Management of Chemotherapy-Induced Peripheral Neuropathy in Survivors of Adult Cancers: ASCO Guideline Update. J. Clin. Oncol..

[126] Klafke N., Homberg A., Glassen K., Mahler C. (2016). Addressing holistic healthcare needs of oncology patients: Implementation and evaluation of a complementary and alternative medicine (CAM) course within an elective module designed for healthcare professionals. BMC Complement. Ther. Med..

[127] Homberg A., Klafke N., Loukanova S., Glassen K. (2020). Findings from a three-round Delphi study: Essential topics for interprofessional training on complementary and integrative medicine. BMC Complement. Med. Ther..

[128] Gupta M., Mamgain R.K., Mamgain P., Verma S.K., Pruthi D.S., Kandwal A., Saini S. (2020). The efficacy of an ayurvedic preparation of yashtimadhu (*Glycyrrhiza glabra*) on radiation-induced mucositis in head-and-neck cancer patients: A pilot study. J. Cancer Res. Ther..

[129] Rastogi S., Tiwari V., Jatav S.P., Singh N., Verma S., Verma S., Sharma K.G., Pandey P., Singh G. (2022). A survey of patients visiting an Ayurvedic teaching hospital for factors influencing the decision to choose ayurveda as a health care provider. J. Ayurveda Integr. Med..

[130] Wortmann J.K., Bremer A., Eich H., Wortmann H.K., Schuster A., Fühner J., Büntzel J., Muecke R., Prott F., Huebner J. (2016). Use of complementary and alternative medicine by patients with cancer: A cross-sectional study at different points of cancer care. Med. Oncol..

[131] Shorofi S.A., Arbon P. (2017). Complementary and alternative medicine (CAM) among Australian hospital-based nurses: Knowledge, attitude, personal and professional use, reasons for use, CAM referrals, and socio-demographic predictors of CAM users. Complement. Ther. Clin. Pract..

[132] Cırık V., Efe E. (2018). Pediatric Nurses’ Usage and Experience Toward Complementary Health Approaches. J. Altern. Complement. Med..

[133] Bhoo-Pathy N., Subramaniam S., Khalil S., Kimman M., Kong Y.-C., Ng C.-W., Bustamam R.S., Yip C.-H. (2021). Out-of-Pocket Costs of Complementary Medicine Following Cancer and the Financial Impact in a Setting with Universal Health Coverage: Findings from a Prospective Cohort Study. JCO Oncol. Pract..

[134] Chang H.Y., Chang H.L. (2015). A review of nurses’ knowledge, attitudes, and ability to communicate the risks and benefits of complementary and alternative medicine. J. Clin. Nurs..

[135] Bossert J., Mahler C., Boltenhagen U., Kaltenbach A., Froehlich D., Szecsenyi J., Wensing M., Joos S., Klafke N. (2022). Protocol for the process evaluation of a counselling intervention designed to educate cancer patients on complementary and integrative health care and promote interprofessional collaboration in this area (the CCC-Integrativ study). PLoS ONE.

[136] Peters M.D.J., Godfrey C.M., Khalil H., McInerney P., Parker D., Soares C.B. (2015). Guidance for conducting systematic scoping reviews. Int. J. Evid. Based Healthc..

[137] van der Steen J.T., ter Riet G., van den Bogert C.A., Bouter L.M. (2019). Causes of reporting bias: A theoretical framework. F1000Research.

