# Supplementary material for: Prevention and Treatment of Chemotherapy-Induced Peripheral Neuropathy (CIPN) with Non-Pharmacological Interventions: Clinical Recommendations from a Systematic Scoping Review and an Expert Consensus Process
Source: Med Sci (Basel). 2023 Jan 30;11(1):15. doi: 10.3390/medsci11010015 (PMC9944490; doi:10.3390/medsci11010015)
Supplement: Supplementary file 1 [file medsci-11-00015-s001.zip › Supplementary S2, Search strategy.pdf]

Supplementary S2. Search strategy

Medline

[((((("Neoplasms"[Mesh]) AND "Holistic Nursing"[Mesh]) OR "Nursing"[Mesh]) OR "Evidence-Based Nursing"[Mesh]) OR "Oncology Nursing"[Mesh]) OR "Complementary Therapies"[Mesh]) AND " Polyneuropathies "[Mesh]] OR [(((application[Title/Abstract]) AND cancer[Title/Abstract]) OR oncolog*[Title/Abstract]) AND neuropathy[Title/Abstract]] OR ((((compression[Title/Abstract]) AND cancer[Title/Abstract]) OR oncolog*[Title/Abstract]) AND neuropathy[Title/Abstract]] OR [(((footbath[Title/Abstract]) AND cancer[Title/Abstract]) OR oncolog*[Title/Abstract]) AND neuropathy[Title/Abstract]] OR [(((handbath[Title/Abstract]) AND cancer[Title/Abstract]) OR oncolog*[Title/Abstract]) AND neuropathy[Title/Abstract]] OR [(((oil[Title/Abstract]) AND cancer[Title/Abstract]) OR oncolog*[Title/Abstract]) AND neuropathy[Title/Abstract]] OR [(((aromatherapy[Title/Abstract]) AND cancer[Title/Abstract]) OR oncolog*[Title/Abstract]) AND neuropathy[Title/Abstract]] OR [(((Tactile[Title/Abstract]) AND cancer[Title/Abstract]) OR oncolog*[Title/Abstract]) AND neuropathy[Title/Abstract]] OR [(((showers[Title/Abstract]) AND cancer[Title/Abstract]) OR oncolog*[Title/Abstract]) AND neuropathy[Title/Abstract]] OR [(((flaxseed[Title/Abstract]) AND cancer[Title/Abstract]) OR oncolog*[Title/Abstract]) AND neuropathy[Title/Abstract]] OR [(((aconit[Title/Abstract]) AND cancer[Title/Abstract]) OR oncolog*[Title/Abstract]) AND neuropathy[Title/Abstract]] OR [(((arnica[Title/Abstract]) AND cancer[Title/Abstract]) OR oncolog*[Title/Abstract]) AND neuropathy[Title/Abstract]] OR [(((copper[Title/Abstract]) AND cancer[Title/Abstract]) OR oncolog*[Title/Abstract]) AND neuropathy[Title/Abstract]] OR [(((peppermint[Title/Abstract]) AND cancer[Title/Abstract]) OR oncolog*[Title/Abstract]) AND neuropathy[Title/Abstract]] OR [(((eucalyptus[Title/Abstract]) AND cancer[Title/Abstract]) OR oncolog*[Title/Abstract]) AND neuropathy[Title/Abstract]] OR [(((rosemary[Title/Abstract]) AND cancer[Title/Abstract]) OR oncolog*[Title/Abstract]) AND neuropathy[Title/Abstract]] OR [(((sugar oil[Title/Abstract]) AND cancer[Title/Abstract]) OR oncolog*[Title/Abstract]) AND neuropathy[Title/Abstract]] OR [(((hypothermia[Title/Abstract]) AND cancer[Title/Abstract]) OR oncolog*[Title/Abstract]) AND neuropathy[Title/Abstract]]

CINAHL

((((MH "Disease Management") OR TI Disease Management OR AB Disease Management) OR ((MH "Alternative Therapies+") TX alternative N0 (TI Therapies AB Therapies)) OR ((MH "Nursing Care+") OR TI Nursing Care OR AB Nursing Care) OR ((MH "Nursing Practice+") TX Nursing N0 (TI Practice AB Practice)) OR ((MH "Rehabilitation+") OR TI Rehabilitation OR AB Rehabilitation) OR (MH "Rehabilitation Nursing") OR ((MH “Preventive health care”) OR TI Preventive health care OR AB Preventive health care))) AND (((MH "Polyneuropathies") OR TI Polyneuropathies OR AB Polyneuropathies) OR TI "Chemotherapy-Induced Peripheral Neuropathy" OR AB "Chemotherapy-Induced Peripheral Neuropathy" OR TI "Chemotherapy-Induced Peripheral Neuropathies" OR AB "Chemotherapy-Induced Peripheral Neuropathies" OR TI CIPN OR AB CIPN) AND ((TI oncology patients OR AB oncology patients) OR ((MH neoplasm*) OR TI neoplasm* OR AB neoplasm) OR ((MH cancer patients) OR TI cancer patients OR AB cancer patients)))
