# Supplementary material for: Prevention and Treatment of Chemotherapy-Induced Peripheral Neuropathy (CIPN) with Non-Pharmacological Interventions: Clinical Recommendations from a Systematic Scoping Review and an Expert Consensus Process
Source: Med Sci (Basel). 2023 Jan 30;11(1):15. doi: 10.3390/medsci11010015 (PMC9944490; doi:10.3390/medsci11010015)
Supplement: Supplementary file 1 [file medsci-11-00015-s001.zip › Supplementary S3, Table S1.pdf]

## Supplementary S3

## Table S1. Characteristics of selected studies included in the scoping review (nursing interventions for CIPN)

| Treat-ments | Study[1]/Country (cultural context) | Study design | Outcome measures (Patient-reported and others) | Patients (gender, tumor entity) | Intervention group (IG)/control group (CG) | Who did conduct the intervention? | Concept | Results/Conclusion[2] |
|---|---|---|---|---|---|---|---|---|
| Phytotherapy | Arslan et al. 2020 [17]<br><br>Konya, Turkey | RCT | CIPN assessment tool (CIPNAT) | Women (n=60) receiving oxaliplatin-based treatment. | IG (n=30): **Henna application (HA)** for 8-10 hours, three 15-day treatment courses.<br><br>CG (n=30): routine care | Practitioner, health care staff | Henna's nectar exhibits biological activities such as antimicrobial, analgesic, anti-inflammatory, and antipyretic effects. | **HA** on hands and feet has a significant beneficial effect on peripheral neuropathy.<br>__________________________<br>Overall: **HA** is a low cost and safe intervention, well tolerated by patients. Henna may be effective in preventing CIPN. |
| | Fallon et al. 2015<br><br>Edinburgh, UK<br><br>In S3 clinical guideline Supportive therapy for cancer patients, February 2020 [37] | Proof of concept study | Measurement of pain symptoms (total Brief Pain Inventory score and improvements in functionality and sensitivity (Quantitative Sensory Testing (QST)). | Patients with chronic PNP (n=51) including n=31 with CIPN. | Twice a day application of **menthol** (**Men)** crème 1% on the painful area. 4-6 weeks. | Not described | Activation of TRP (transient receptor potential) ion channels. Molecular receptors for cooling have been identified in sensory nerves with evidence for their upregulation in neuropathic pain models. | Therapy of CIPN: Of the 38 evaluable patients, 31 had a significant reduction in pain symptoms: median 47/ 34; $p < 0.001$), plus an improvement in functionality and sensitivity.<br>__________________________<br>Overall: Expert consensus in S3 clinical guideline for consideration of topical therapy **(ToT)** of CIPN with 1% **Men** may be considered. |
| | Izgu et al.2019 [101]<br><br>Ankara, Turkey | Open-label, parallel-group, quasi-randomized controlled pilot study | Self-developed patient questionnaire (sociodemographic characteristics, type of cancer, treatment scheme, neuropathic symptoms), numeric rating scale (NRS), Douleur Neuropathique 4 Questions, Piper Fatique Scale | 46 cancer patients receiving oxaliplatin and suffering from CIPN and fatigue | IG (n=22) **Aromatherapy (AT)** hand and foot **Massage (M),** 18 sessions á 40 minutes (peppermint, chamomile, rosemary essential oils 1,5 % in coconut oil), six weeks.<br><br>IG (n = 24): standard medical care | Principle investigator (PI) who is qualified in massage therapy. | Because of their analgesic, antinociceptive, neuroprotective, and blood circulation-enhancing effects, peppermint, chamomile, and rosemary essential oils were used. | At week six, the rate of neuropathic pain was significantly lower in the IG, when compared with the CG. The severity of pain based on NRS was significantly lower in the IG than in the CG at week 2, 4, and 6. At week 8, fatigue in the IG was significantly lower in the IG than in the CG.<br>__________________________<br>Overall: **AT M** may be useful in the management of CIPN and fatigue. |
| | Li et al. 2019 [35]<br><br>Zhejiang, China | Meta-analysis | World Health Organization | Cancer Patients (>18 years), | IG: All types of chinese **herbal medicine** (**Phy)** in | Not reported | Influencing qi stagnation, promote blood circulation, dredging collaterals to | Chinese **Phy** was found to improve SNCV (sensory nerve conduction velocity) and MNCV (motor nerve conduction velocity). |

[1] The number in brackets refer to the comprehensive reference list (see full article).
[2] A list of all abbreviations can be found at the end of Table S1.

| Treat-ments | Study[1]/Country (cultural context) | Study design | Outcome measures (Patient-reported and others) | Patients (gender, tumor entity) | Intervention group (IG)/control group (CG) | Who did conduct the intervention? | Concept | Results/Conclusion[2] |
|---|---|---|---|---|---|---|---|---|
| | | 20 trials including 1481 participants. Number of patients in each arm was more than 15 | (WHO) grade, CIPN grade or Levi's grade]. | Chemotherapy, CIPN diagnosed by clinical assessment. | TCM there of **hand-footbath (HFB),** compress, decoction, Radix aconiti, tuber aconiti, salvia, ginger<br><br>CG: No treatment, placebo, conventional therapeutic agents | | remove meridian obstruction, improve microcirculation in nervous system, reducing the nerve injury. | For all grade CIPN as well as high grade CIPN - preventive and curative.<br>____________________________<br>Overall: Evidence is not sufficient to draw definitive conclusion. |
| | Noh et al. 2018 [36]<br><br>Seoul, Republic of Korea (South Korea) | Systematic Review of RCTs<br><br>28 trials, 2174 participants. | Clinical improvement, incidence rate, and nerve conduction study (NCS) | 18 years or older, diagnosed with cancer, and CIPN | IG: All types of **phytotherapy** (**Phy)** used for medicinal purposes (e.g., oral, intravenous infusion using fluid, intravenous injection, fumigation, external application, or hand and foot baths)<br><br>CG: no treatment, a placebo or conventional therapeutic agents | Not described | **Phy** based on unique theories of Yin and Yang, the five elements, and the visceral manifestation theory | Preventive Effects: **Phy** for **hand and foot baths (HFB)** or fumigation: significantly effective in decreasing the incidence rate.<br><br>Therapeutic Effects: **Phy** for **HFB** or fumigation: The wenloutong formula (contains Aconitituber) and the wenjing hu-oxue ton-gluo formula (contains aconiti tu-ber) were as significantly effective as warm water baths. The effectiveness rate of the wenjing tongluo formula was significantly improved in comparison to the placebo **HFB**. The effectiveness rate of the wenyang tongbiformula presented a significant improvement in comparison-to Vitamin B12 **(VB).**<br>____________________________<br>Overall: **Phy** have potentially preventive and/or therapeutic effects for CIPN. Due to the characteristics of CIPN—the direct application would be considered an effective dosage form. |
| | Noh & Park 2019 [50]<br><br>Seoul, South Korea | RCT | CIPN assessment tool, Hospital Anxiety-Depression scale (HADS), Peripheral skin temperature | Gynecologic cancer patients (n=66) who received chemotherapy (Taxane, platinum group) | IG (n=32): **Aroma foot-reflexology (AT-FR)** three times a week, 15 min for each foot over six weeks (mandarin, black pepper, pine, and tea tree essential oils in argan oil, 3 % dilution). | Participants themselves after instruction, biweekly phone monitoring by researcher. | Reflexology promotes blood circulation and lymphokinesis, metabolism. and physical and mental relaxation. Increased skin temperature induces psychological relaxation.<br><br>Essential Oils: Black pepper improves blood | No harm or unanticipated risk.<br><br>Statistically significant reduction of PNP symptoms ($t=5.26$, $p < .001$), increased skin temperature (($t=-4.41$, $p < .001$) and in IG.. Anxiety and depression decreased in the experimental group ($p < .001$). No significant difference between IG and CG with regard to mild to severe anxiety and depression.<br>____________________________<br>Overall: **AT-FR** can reduce CIPN. |

[1] The number in brackets refer to the comprehensive reference list (see full article).
[2] A list of all abbreviations can be found at the end of Table S1.

| Treat-ments | Study[1]/Country (cultural context) | Study design | Outcome measures (Patient-reported and others) | Patients (gender, tumor entity) | Intervention group (IG)/control group (CG) | Who did conduct the intervention? | Concept | Results/Conclusion[2] |
|---|---|---|---|---|---|---|---|---|
| | | | | | CG (n=31): identical aroma foot-reflexology 6 weeks later | | circulation and has pain reducing effects, pine improves relaxation, mandarin mood elevation. | |
| | Rostami et al. 2019 [75]<br><br>Teheran, Iran | RCT | Functional Assessment of Cancer Therapy/Gynecologic Oncology Group (FACT/GOG) - Neurotoxicity score | Cancer patients (n= 34) between 18 to 80 years, chemotherapy, CIPN<br><br>Exclusion: Diabetes, brain metastasis, renal failure, carpal tunnel syndrome | IG (n=18): **topical c. colocynthis (TCC)** (bitter apple fruit extract in sesam oil) two times per day for 2 months<br><br>CG (n=16):<br>Placebo oil (sesam oil) | Participants themselves after instruction | Traditional Persian medicine. **TCC** as a therapeutic agent with simultaneous antioxidative and anesthetic effects | No significant improvement in the total score of FACT/GOG-Ntx scale and placebo groups.<br><br>No significant improvement in FACT/GOG-Ntx in the sensory, motor, hearing, and functional domains in the two study groups.<br>________________________<br>Overall: **TCC** oil failed to improve the symptoms of CIPN compared with Placebo. |
| Movement therapy | Steimann et al. 2011, Lehmrade, Germany<br><br>In S3 clinical guideline Supportive therapy for cancer patients, February 2020 [37] | Overview Article | Subjective assessment of patients | Cancer Patients | **Sensorimotor training** (**SM**), **tactile stimulation** (**TS**), **exercises** (**E**) | Not described | **SM**, **TS**: To improve or normalise nerve activity through mechanical stimuli. | 81% of patients consider **TS** to be very effective or effective.<br>________________________<br>Overall: Expert consensus that **SM,** e.g., been bath for prevention of CIPN can be done. Instructions for regular functional training can already be given. Regular movement training, especially of the finger and toe functions. |

[1] The number in brackets refer to the comprehensive reference list (see full article).
[2] A list of all abbreviations can be found at the end of Table S1.

| Treat-ments | Study[1]/Country (cultural context) | Study design | Outcome measures (Patient-reported and others) | Patients (gender, tumor entity) | Intervention group (IG)/control group (CG) | Who did conduct the intervention? | Concept | Results/Conclusion[2] |
|---|---|---|---|---|---|---|---|---|
| Cryotherapy | Bandla et al. 2019 [18]<br><br>Singapore, Singapore | Proof-of-concept study | Total neuropathy score (TNS), nerve conduction studies (NCS) | Cancer patients (n=26) receiving taxane (21–80 years); no history of neuropathy; Eastern Cooperative Oncology Group (ECOG) performance status 0; and no history of hospitalization in the past 6 months | IG (n=13): four-limb **Cryocompression (CC)**, 16 °C and cyclic pressure of 5–15 mmHg, with each chemotherapy infusion for a maximum of 12 cycles.<br><br>CG (n=13): continuous-flow cooling and no cooling. | Not described | Coolant temperature of 22°C was determined to be the lowest tolerable temperature in healthy volunteers for a duration of 3 h (**CC** attains a lower temperature faster than **CT** indicating that compression increases the rate of cooling. | **CC** demonstrated significantly greater skin temperature reductions compared to continuous-flow cooling and control.<br><br>**CC** may potentially improve the efficacy of preventing CIPN compared to continuous-flow limb cooling.<br>___<br>Overall: **CC** is a safe and tolerable therapy for the relief of CIPN in cancer patients undergoing taxane treatment. However, this remains to be demonstrated in further studies. |
| | Beijers et al. 2020 [39]<br><br>Veldhoven, Netherlands | RCT | CIPN20 | Cancer patients (n=180)<br><br>Tumor:<br>Colorectal: 53<br>Prostate: 2<br>Breast: 31<br>Other: 4 | IG (n=90): **Frozen glove (FG)** and sock for 15 min prior to and 15 min after completion of the paclitaxel.<br><br>CG (n=90): Care without FGs | Patient himself in this study | Not described | IG experienced significantly reduced tingling in fingers/and less trouble opening a jar or bottle due to loss of strength in hands.<br>___<br>Overall: Wearing **FGs** might reduce some neuropathy symptoms in the hands. However, one-third of the IG discontinued the study before the end of treatment. Future studies should focus on the method of limb **Hypo** to prevent CIPN. |
| | Griffiths et al. 2018 [19]<br><br>Chicago, Illinois, USA | RCT | Neuropathic Pain Symptom Inventory, Brief Pain Inventory, quantitative sensory testing. | Breast cancer patients (n=29) receiving paclitaxel | IG: **Cryotherapy (CT)**. Frozen glove and sock for 15 min prior to and 15 min after completion of the paclitaxel.<br><br>CG: participants as their own paired control. | Clinicians | Not described | No significant differences in measures of neuropathy or pain between treated and untreated hands or feet.<br><br>High drop-out rate, more than half of the patient sample could not well tolerate **CT**<br>___<br>Overall: This RCT was unable to demonstrate benefit of **CT**. |
| | Sundar et al. 2017 [40]<br><br>Singapore, Singapore | Prospective pilot Study | Visual analog pain scale (VAS), subjective tolerance scale, | Breast cancer patients (n=20) scheduled to receive adjuvant weekly | Limb **Hypothermia (Hypo)** sessions comprised of a pre-cooling period (1 h), continued with paclitaxel infusion and | Not described | Reduction of nerve blood flow | Continuous-flow limb Hypothermia was well tolerated by all patients. Differences in nerve conduction parameter did not reach significance.<br>___ |

[1] The number in brackets refer to the comprehensive reference list (see full article).
[2] A list of all abbreviations can be found at the end of Table S1.

| Treat-ments | Study[1]/Country (cultural context) | Study design | Outcome measures (Patient-reported and others) | Patients (gender, tumor entity) | Intervention group (IG)/control group (CG) | Who did conduct the intervention? | Concept | Results/Conclusion[2] |
|---|---|---|---|---|---|---|---|---|
| | | | the Shivering Assessment Scale<br>nerve conduction studies (NCSs) | paclitaxel chemotherapy for 12 cycles | a post-cooling period (on average 30 min after the end of paclitaxel infusion) | | | Overall: New opportunity in neuroprotection and preventing CIPN via a simple, non-invasive, and non-pharmacological method. |
| Manipulative therapies | Izgu et al. 2019 [41]<br><br>Ankara, Turkey | RCT | Self-Leeds Assessment of Neuropathic Symptoms and Sign (S-LANSS), EORCT QLQ CIPN20. Nerve conduction studies (NCS). | Breast cancer patients (n=40) receiving adjuvant paclitaxel | IG: Classical **massage** (**M**) before each paclitaxel infusion<br><br>CG: patients received only usual care | Principal investigator with nursing background, who was qualified and experienced in **M** therapy. | Classical **M** (Swedish **M)** affects regulation of muscles, joints, tendons and ligaments in the body by manipulation of soft tissues with effleurage, petrissage, friction and tapotement techniques. | CIPN was lower in the IG compared to the CG at week 12.<br><br>Sensory and motor sub-scales scores of the QoL measure showed statistically significant differences over time in favor of IG.<br><br>Sensory action potential amplitude of the median nerve was significantly higher and the tibial nerve latency was significantly shorter in IG at week 12.<br>____________________________<br>Overall: Classical **M** successfully prevented CIPN, improved the QoL, and showed beneficial effects on the NCS findings. |
| | Sarisoy, et al. 2020 [76]<br><br>Gaziantep, Turkey | RCT | VAS, Doleur Neuropatique/Neuropatic pain (DN4), Pittsburg Sleep quality Index (PSQI) | Patients (n=40) with non-hodgkin-lymphoma | IG (n=20): **Foot-massage (FM)** 3 times a week, each foot 10 minutes, over four weeks with 2ml of olive oil<br><br>CG (n=20)**:** Clinical routine. | Applied from the researchers (nurses) | Not described | Pain score at first interview was significantly higher than after four weeks<br><br>Pain in the CG is significantly higher than in the IG group.<br>____________________________<br>Overall: **FM** has a positive effect on CIPN pain. |
| Others | Kotani et al. 2021 [43]<br><br>Kanokoden, Chikusa-ku, Nagoya, Japan | Double-blind phase 2 trial | PROs were assessed by using the CTCA v4.0 at each cycle. The primary outcome was the difference in the incidence of Grade ≥ 2 CIPN. | Patients (n=56) with early and recurrent breast cancer (with no prior paclitaxel exposure) receiving weekly paclitaxel with 80 mg/m$^2$ | Intervention: **compression (Com)** therapy (90 min) with surgical gloves – two one size smaller gloves on one hand<br>Control: two normal-size gloves on the other hand. | Patients were instructed by physicians and nurses. | Previous studies confirmed that **Com** reduces CIPN, but its effectiveness regarding prevention has not been assessed under circumstances which aimed to minimize bias. | **Com** was not effective in preventing CIPN.<br>____________________________<br>Overall: **Com** therapy with surgical gloves does not significantly reduce the incidence of paclitaxel-induced CIPN. Further research is needed in this area, as **Com**, as a low-cost and safe therapy, has been evaluated as effective in other studies. |

[1] The number in brackets refer to the comprehensive reference list (see full article).
[2] A list of all abbreviations can be found at the end of Table S1.

## List of abbreviations

| | | | | | | | |
|---|---|---|---|---|---|---|---|
| Acetyl-l-carnitine | AIC | External applications | EAP | Massage | M | Reflexology | R |
| Acupressure | AP | Fatty acids | FA | Meditation | Me | Rehabilitation intervention | RI |
| Acupuncture | A | Fine motor training | FMT | Menthol | Men | Reiki | Rei |
| Aerobic | Ae | Foot massage | FM | Mind-body modalities | MBM | Relaxation | Rel |
| Alpha-lipoic acid | ALA | Frozen gloves | FG | Mind-body therapies | MBT | Resistance training | RT |
| Aromatherapy | AT | Footbath | FB | Movement therapies | MT | Rhythmical embrocations | RE |
| Balance training | BT | Foot reflexology | FR | Music therapy | MuT | Scrambler therapy | ST |
| Cardiovascular exercise | CardE | Glutamine | G | Natural products | NP | Self-management strategies | SMT |
| Chinese medicine | CH | Goshajinkigan | Go | Nerve conduction studies | NCS | Sensorimotor training | SM |
| Closed kinematic chain exercises | CKC-E | Hand and foot baths | HFB | Non-pharmacological interventions | NPI | Sports therapy | SpT |
| Cognitive behavioural intervention | CBI | Healing touch | HeTo | Nurse-led follow-up | NLF | Support groups | SG |
| Complementary and Alternative Medicine | CAM | Heat application | He | Nutritional therapy | NT | Strength training | STr |
| Complementary and Integrative Medicine | CIM | Henna application | HA | Occupational therapy | OT | Tactile stimulation | TS |
| Complementary treatments | CTs | Homeopathy | Ho | Omega-3 | O3 | Tai Chi | TC |
| Compression | Com | Humor therapy | HT | Pain management strategies | PMS | Therapeutic touch | TT |
| Coordination training | CoT | Hydroelectric bath | Heb | Passive mobilisation | PM | Topical C. colocynthis | TCC |
| Cryocompression | CC | Hydrotherapy (Kneipp) | HTK | Patient education | PE | Topical therapy | ToT |
| Cryotherapy | CT | Hyperthermia | HyT | Physical activity | PhA | Traditional Chinese Medicine | TCM |
| Cupping | C | Hypnosis | H | Physical therapy | PT | Transcutaneous electrical nerve stimulation | TENS |
| Cycling | Cy | Hypothermia | Hypo | Physiotherapy | PhyT | Vitamin B12 | VB |
| Dietary modification | DM | Imagery | I | Phytotherapy (incl. herbal medicines, botanical agents) | Phy | Vitamin B complex | VBC |
| Dietary supplements | DS | Integrative therapies | ITs | Positioning | Po | Vitamin E | VE |
| Distraction therapy/training | DT | Kneipp therapy | KT | Problem-solving therapy | PST | Walking | W |
| Electroacupuncture | EA | Magnesium replacement | MR | Progressive muscle relaxation | PMR | Whole-body vibration | WBV |
| Electrotherapy | ET | Magnet/Laser therapy | MLT | Psychosocial approaches | PA | Yoga | Y |
| Exercise (all types) | E | Manual therapy | MaT | Qi Gong | QG | | |

[1] The number in brackets refer to the comprehensive reference list (see full article).
[2] A list of all abbreviations can be found at the end of Table S1.
