# Supplementary material for: Prevention and Treatment of Chemotherapy-Induced Peripheral Neuropathy (CIPN) with Non-Pharmacological Interventions: Clinical Recommendations from a Systematic Scoping Review and an Expert Consensus Process
Source: Med Sci (Basel). 2023 Jan 30;11(1):15. doi: 10.3390/medsci11010015 (PMC9944490; doi:10.3390/medsci11010015)
Supplement: Supplementary file 1 [file medsci-11-00015-s001.zip › Supplementary S4, Table S2.pdf]

Supplementary S4.

Table S2. Characteristics of other relevant studies for treating pain with complementary therapies in cancer patients or other patient populations

Treatments	Study[1]/ Country (cultural context)	Study design	Outcome measures (Patient-reported and others)	Patients (gender, tumor entity)	Intervention group (IG)/ control group (CG)	Who did conduct the intervention?	Concept	Results/Conclusion[2]
Acupuncture, acupressure (TCM)	Deng et al.<br>2013 [53]<br><br>New York City, New York, USA	Systematic review (4 meta-analyses, 14 systematic reviews, and 16 RCTs)	Symptoms of anxiety, mood disturbance, pain, quality of life.	Lung cancer patients	IG: **A**<br>CG: **Vitamin B12 (VB)**	Not described	**A** have been used in all forms of medicinal healing in Chinese medicine (**CH**). Although distinct versions of **A** (Japanese, Korean, French, and so forth) have developed over the years, the essential theory of harmonizing imbalances in the body remains.	One RCT, three prospective case series or reports, and one retrospective review<br><br>There is a paucity of data on whether A could be useful in the treatment of CIPN. Small case series show some improvement regarding VAS and neuropathy symptoms.<br>____________________<br>Overall: data for cancer-related pain are more abundant, the data only support potential benefits in breast and head and neck cancer.
Acupuncture, acupressure (TCM)<br><br>Nutritional therapy<br><br>Other supportive interventions	Rostock et al.<br>2013 [88]<br><br>Freiburg, Germany	RCT (four arm)	Electroneurographic test, NRS Scale	Male and female adult cancer patients (n = 60), who were in remission after chemotherapy with taxanes, platinum derivatives, or vinca alkaloids and who presented with symptoms of CIPN.	IG: (n = 14) 8 +1 sessions, the four treatments spanned 3 weeks, **electroacupuncture (EA)** were with point combination like trigger points, **hydroelectric bath (Heb)** (n = 14) with 35°C water, Patients dip their arms and feet into it for 15 minutes, Water was 50 Hz electric, **vitamin B complex (VBC)** (n = 15): 3 capsules of high-dosage **vitamin** B1/B6 (100 mg thiamine nitrate, 100 mg pyridoxine hydrochloride) per day for three weeks.<br><br>CG: placebo (n= 17): 3 lactose capsules per day identical in form, taste, and odor to the **VBC**.	Specially trained, highly experienced physicians	Not described	There were no statistically significant differences between the treatment groups by electroneurographic test results.<br><br>Health related quality of life also moderately improved in all groups, but without any statistical group differences at day 21.<br>____________________<br>Overall: **EA**, **Heb** and **VBC** give hints for positive effects for CIPN.
Acupuncture, acupressure (TCM)	Wong et al.<br>2016 [93]<br><br>Hamilton, Ontario, Canada	Prospective phase 2 Study	Modified total neuropathy score (mTNS), Numbness score<br>Edmonton Symptoms Assessment Scale (ESAS)	Patients with CIPN (n=40)	**IG:** (n= 27) **A**-like **TENS** twice weekly for 12 treatments over 6 to 8 weeks.<br><br>CGI: (n=13) **A**	Trained clinical trial nurses/therapist	Not described	Statistically significant difference at 6 months from the baseline pain score.<br>**A TENS** can improve CIPN symptoms.<br>____________________<br>Overall: A TENS is effective in reducing symptoms associated with CIPN.

[1] The number in brackets refer to the comprehensive reference list (see full article).
[2] A list of all abbreviations can be found at the end of Table S2.

Supplementary S4.

Treatments	Study[1]/ Country (cultural context)	Study design	Outcome measures (Patient-reported and others)	Patients (gender, tumor entity)	Intervention group (IG)/ control group (CG)	Who did conduct the intervention?	Concept	Results/Conclusion[2]
Acupuncture, acupressure (TCM)	S3 guideline complementary medicine in the treatment of oncology patients [63]<br><br>Nov 2020<br><br>AWMF, DKG, German Cancer Aid, PRIO<br><br>Ju et al. (2017) – China;<br>Greenlee et al. (2016) - USA<br>Molassiotis, (2019) – Hong Kong	1 Meta-analysis including 104 patients, 2 RCTs including 150 patients	Brief Pain Inventory, CTCAE, Total Neuropathy Score, nerve conduction studies (NCS); and patient-reported outcome measures (Functional Assessment of Cancer Therapy–Gynecologic Oncology Group–Neurotoxicity Quality of Life scale, and Symptom Distress Scale)	Cancer patients (Breast cancer Ovarian cancer, Head/neck cancer, breast cancer, colorectal cancer and myeloma patients) with CIPN	IG: Standard therapy + Mecobalamin + **A** treated with either electro-**A** or sham **A** for 12 weeks (1x per week) before starting chemotherapy, in addition to standard treatment (Ju et al. 2017)<br><br>During and after chemotherapy treated over 8 weeks additionally 2x per week with **A** (Molassiotis et al. 2019)<br><br>CG:<br>Standard therapy + Mecobalamin<br>Standard therapy (Ju et al. 2017) treated either with standard therapy only (Molassiotis et al.2019)	Acupuncturists	From a traditional point of view, **A** is able to regulate the flow of qi in the pathways, expel pathogenic factors, dissolve obstructions and treat disharmonies of the internal organs. From a Western scientific point of view, **A** can be understood as a stimulus event that triggers pain-Inhibiting and regulation-promoting mechanisms at the neuronal, vegetative and hormonal levels via local and systemic points of action	2 RCTs included.<br>Post-intervention, **A** was significantly superior to standard treatment<br><br>No group differences were found immediately after the end of 12 weeks<br><br>After a 16-week follow-up, those treated with Sham **A** had significantly less pain than the verum patients. In the IG, 1 mild adverse event was recorded<br><br>Significant Improvements in pain interference, neurotoxicity-related symptoms<br>__________<br>Overall: Data are available from a meta-analysis and two RCTs on the efficacy of A- for CIPN. **A** may be considered for these complaints.
Acupuncture, acupressure (TCM)	Donald et al. 2011 [68]<br><br>Manchester, UK	Retrospective service evaluation	CIPN symptoms. No validated questionnaires (sleeping, relaxing, stress, medication, mood)	18 patients (eight males, 44%, and ten females, 56%) with different cancers (Haemato-oncology, gastrointestinal, breast, gynecological) with CIPN	IG: A course of six weekly **A** session was offered to the patients	Acupuncturists	The similarity between the results for diabetic neuropathy and those described here suggests that there may be aspects of neuropathy that are common across differing aetiologias and that **A** could be effective in addressing this commonality.	82% (n=14) reported that **A** had improved their neuropathy symptoms<br><br>18% (n=3) reported no change and there were no reports of deterioration<br><br>35% (n=6) reported one additional benefit from the **A**<br><br>41% (n=7) documented more than one additional benefit.<br><br>24% per cent of the patients (n=4) had no additional benefits to report from the treatment.<br>__________<br>Overall: **A** may be worth further

[1] The number in brackets refer to the comprehensive reference list (see full article).
[2] A list of all abbreviations can be found at the end of Table S2.

Supplementary S4.

Treatments	Study[1]/ Country (cultural context)	Study design	Outcome measures (Patient-reported and others)	Patients (gender, tumor entity)	Intervention group (IG)/ control group (CG)	Who did conduct the intervention?	Concept	Results/Conclusion[2]
								exploration in the management of CIPN. Pain was not assessed with a validated questionnaire; small sample size is a limitation.
Movement therapies<br><br>Mind-body therapies<br><br>Nutritional therapy<br><br>Acupuncture, acupressure (TCM)<br><br>Manipulative therapies<br><br>TENS/Scrambler therapy	Armstrong et al. 2005 [102]<br><br>Bethesda, South Carolina, USA	Review	Not described	Cancer patients treated with chemotherapy	Conventional: **Exercise (E), Physical therapy (T), Massage (M), Transcutaneous electrical nerve stimulation (TENS)**<br><br>Unconventional (possibly helpful): **Magnesium replacement (MR), Distraction therapy/training (DT), Humor therapy (HT), Acupuncture (A)**<br><br>Unconventional (probably not helpful): **Magnet therapy, Dietary modification (DM)**	Physical therapists can administer **TENS**. Other providers of other therapies have not been described.	Some patients may derive benefit from **Complementary and Integrative Medicine (CIM)** because they feel the need to do something, whereas others may derive benefit from a placebo effect. With its significant impact on quality of life, CIPN treatment and prevention (including CIM) are important in the care of patients with cancer	**A:** little data exist<br><br>**E:** may produce general benefits<br><br>**TENS**: may increase endorphin release, block noxious sensory impulses through distraction.<br><br>**M:** significant reduction in pain<br><br>**MR**: no effect<br>____________________<br>Overall: No information on methods and number of included studies were reported.
Movement therapies<br><br>Mind-body therapies<br><br>Nutritional therapy<br><br>Acupuncture, acupressure (TCM)<br><br>Manipulative therapy	Brami et al. 2016 [16]<br><br>Paris, France	Systematic review (13 RCTs including 1370 patients)	Nerve conduction velocity, Neurological Disability Score, Neurological Symptom Score, Total Neuropathy Score, Quality of life.	Cancer patients treated with platinum-derived compounds, vinca alkaloids, taxanes, and theproteasome inhibitor bortezomib	IG: Patients received **natural products (NP)** and **complementary treatments** (**CTs**) to address CIPN.<br><br>CG: Patients were non-supplemented or received Placebo.	Healthcare practitioners	These modalities are clearly distinguished from alternative therapies that forego mainstream treatment and lack scientific Evidence of safety and efficacy	12 RCTs evaluated **NP** and one evaluated **electroacupuncture (EA).**<br><br>**Vitamin E (VE)** may help prevent CIPN, **Glutamine (G), Goshajinkigan (Go), and Omega-3 (O3)** trials also promising.<br><br>**Acetyl-l-carnitine (AlC)** may worsen CIPN, **Alpha-lipoic-acid** (**ALA)** activity is unknown.<br><br>**EA** was not superior to placebo. **Touch therapies** (massage) (**TT**) reduced greatly CIPN symptoms from grade 2 to 1.<br>Evidence was also reported for, **mind-body modalities** (**MBM**) and **physical activity** (**PhA**).<br>____________________

[1] The number in brackets refer to the comprehensive reference list (see full article).
[2] A list of all abbreviations can be found at the end of Table S2.

Supplementary S4.

Treatments	Study[1]/ Country (cultural context)	Study design	Outcome measures (Patient-reported and others)	Patients (gender, tumor entity)	Intervention group (IG)/ control group (CG)	Who did conduct the intervention?	Concept	Results/Conclusion[2]
								Overall: further research is needed given the lack of effective CIPN interventions.
Movement therapies<br><br>Acupuncture, acupressure (TCM)<br><br>TENS/Scrambler therapy	Denlinger et al. 2019 [98]<br><br>Philadelphia, Pennsylvania, USA	Overview Article of NCCN Guidelines for Survivorship	Different pain scores	Cancer survivors	**Physical therapy (PT):** 150 min/wk of **aerobic (Ae)** plus supervised **strength training (STr)** twice per week<br><br>**Acupuncture (A)**: Control: wait list<br><br>**Transcutaneous electrical nerve stimulation TENS**: no information<br><br>**Scrambler therapy (ST)**: no information<br><br>Control: Usual care	**TENS**:<br>No information<br><br>**ST:** pain specialist<br><br>**A**: no information<br><br>**PT:** no information	Not described	**TENS:** recommended as a non-pharmacological therapy for reducing cancer-related pain<br><br>**ST**: studies in patients with cancer pain show that it appears safe and may be effective at reducing pain<br><br>**A**: small but statistically significant reduction in joint pain at 6 weeks<br><br>**PT**: experienced greater improvements in worst joint pain scores, pain severity, and pain interference than those in the usual care arm.<br>________________________<br>Overall: The panel agreed that the trials represented high-quality data. The guidelines are meant to be a tool to help oncologists better address the needs of survivors who are undergoing surveil-lance and those on chronic treatments.
Movement therapies<br><br>Mind-body therapies<br><br>Acupuncture, acupressure (TCM)<br><br>Manipulative therapies<br><br>Other TCM therapies	Olsson Möller et al. 2019 [64]<br><br>Kristianstad, Sweden	Syst. Review of syst. Reviews (n=37)	Pain, fatigue and quality of life (QoL) were assessed	Patients ≥18 years, breast cancer, undergone breast cancer treatment	IG: Single or combined **Exercise (E)** and **physical activity (PhA)**, **CAM**: **Tai Chi (TC), acupressure (AP), massage (M), Yoga (Y)**, lymphoedema treatment, and psychosocial interventions<br><br>CG**:** Predefined CG	Not reported	A systematic way of providing individualized rehabilitation. "Who needs what and when."	37 Studies included.<br>**E** improved outcomes such as shoulder mobility, lymphoedema, pain, fatigue and quality of life (QoL).<br><br>**AP** may be beneficial to reduce chemotherapy-induced nausea and vomiting.<br><br>M can reduce fatigue.<br><br>Effects of **Y** were shown on QoL, anxiety, depression, sleep disturbance, fatigue and gastrointestinal symptoms.

[1] The number in brackets refer to the comprehensive reference list (see full article).
[2] A list of all abbreviations can be found at the end of Table S2.

Supplementary S4.

**Treatments**	**Study[1]/ Country (cultural context)**	**Study design**	**Outcome measures (Patient-reported and others)**	**Patients (gender, tumor entity)**	**Intervention group (IG)/ control group (CG)**	**Who did conduct the intervention?**	**Concept**	**Results/Conclusion[2]**
								The effect of **CAM** was shown on nausea, pain, fatigue, anger and anxiety.<br>Overall: More than one intervention could have positive effects on a specific symptom and that the effects depend not only on intervention type but also on how and when the intervention is provided. Results need to be interpreted with caution because of low methodological quality in included studies.
Cupping	Kim et al. 2011 [55]<br><br>Daejeon, Republic of Korea (South Korea)	Systematic review (7 RCTs)	Pain scores.	Adult patients with pain of all origin	IG: **Cupping (C)** with or without drawing blood<br><br>CG: Placebo or another treatment or no treatment	Not reported	Create suction on the skin over a painful area or **acupuncture (A)** point.<br>Interruption of blood circulation and congestion as well as stopping the inflammatory extravasations (escaping of bodily fluids such as blood) from the tissues. Others have postulated that **C** could affect the autonomic nervous system and help to reduce pain.	Two RCTs suggested significant pain reduction for **C** in low back pain compared with usual care and analgesia.<br><br>Two RCTs showed positive effects of **C** in cancer pain and trigeminal neuralgia compared with anticancer drugs and analgesics.<br><br>Two RCTs reported favorable effects of **C** on pain in brachialgia compared with usual care (*p*= .03) or heat pad (*p*< .001).<br><br>One RCT failed to show superior effects of **C** on pain in herpes zoster compared with anti-viral medication (P = .065). Currently there are few RCTs testing the effectiveness of **C** in the management of pain.<br>Overall: Some suggestive evidence for the effectiveness of C. Methodological quality were too low to draw firm conclusions.
Hydrotherapy	Koch 2015 [82]<br><br>Berlin, Germany	RCT waiting group design	Measurement instruments for numbness,	Adults (n=64) 18 to 75 years. 23 female, 41 male,	Home-based self-treatment with **hydrotherapy HTK** (local cold stimulus) over 4 weeks	Patients themselves after Instruction	The cold stimulus could influence the	No significant difference of symptom scores between both groups.

[1] The number in brackets refer to the comprehensive reference list (see full article).
[2] A list of all abbreviations can be found at the end of Table S2.

Supplementary S4.

Treatments	Study[1]/ Country (cultural context)	Study design	Outcome measures (Patient-reported and others)	Patients (gender, tumor entity)	Intervention group (IG)/ control group (CG)	Who did conduct the intervention?	Concept	Results/Conclusion[2]
			heaviness/swelling sensation, insensitivity, NRS Polyneuropathy Score PUS, Quality of life, SF 36.	age 63 ± 7,7. Slight to medium intensity of polyneuropathy. PUS > 3			functioning of neuronal nets, and stimulation of blood flow could improve neuronal functions	Overall: **HTK** self-care is easily applicable and was well accepted by the patients. Further studies with larger populations, should evaluate, whether a longer application period could induce more pronounced effects.
Hydrotherapy	Stier-Jarmer et al 2021 [59]<br><br>Munich, Germany	Systematic Review (n= 25 sources, 14 RCTs including 909 patients, 11 uncontrolled studies including 2356 patients)	Pain, polyneuropathic complaints quality of life.	Healthy and sick people	All five elements of **Kneipp therapy (KT): hydrotherapy (HTK), phytotherapy (Phy), exercise (E), dietary modification (DM), mind-body modalities (MBM).** Duration of intervention: 1 day to 12 months.	Not described	Not described	3 studies rated "strong", 13 "moderate" and 9 "weak". Nine (64%) of the controlled studies reported significant improvements after **KT** for chronic venous insufficiency, hypertension, mild heart failure, menopausal symptoms and sleep disorders as well as improved immune parameters in healthy subjects. **HTK** led in one observational study to significant improvements in patients with polyneuropathic complaints (paresthesia). No significant effect in one RCT.<br><br>Overall: **KT** seems to be effective in numerous complaints e.g. sleep disorders. With regard to disease-related polyneuropathic complaints no statistically significant effect.
Hydrotherapy	Uehleke et al. 2008 [91]<br><br>Berlin, Germany	Preliminary open prospective observational "study by correspondence"	Likert scales 0-6 rating PNP symptoms (pain, paresthesia, numbness, paresis, other complaints, general impairment and feeling)	27 patients with PNP (15 females, 12 males; mean age 68.2 years) with peripheral neuropathy (PNP)	Home-based self-treatment. Daily use of at least two out of four **hydrotherapy HTK** interventions (knee affusion, cold **footbath (FB)**, alternating foot-bath, wet socks)	Patients themselves	Cold stimulus could influence neuronal networking and that improvement of circulation and metabolism could improve local neuronal function.	Significant decrease in dysesthesia from a mean of 3.4 (baseline) to 2.9 after the cure.<br><br>Hypesthesia (numbness) improved from 3.5 to 3.1. There were no significant changes for total pain (2.6 to 2.5) and paresis (0.8 to 0.8).<br><br>Overall: Hints for effectiveness of self-treatment with **HTK** in patients with PNP.
Manipulative therapies	Dyer et al. 2013 [46]<br><br>London, UK	Non-blinded, randomised study	MYCaW scores; VAS, relaxation	Different cancers: breast patients (n= 115), CUP,	IG: **Aromatherapy-Massage AT-M** (n=58), **Reflexology (R)** (n=57), four treatments	An initial appointment	**AT** is the systematic, controlled use of	**R** was found to be no less effective than **AT-M** for MYCaW first concerns. There was no

[1] The number in brackets refer to the comprehensive reference list (see full article).
[2] A list of all abbreviations can be found at the end of Table S2.

Supplementary S4.

Treatments	Study[1]/ Country (cultural context)	Study design	Outcome measures (Patient-reported and others)	Patients (gender, tumor entity)	Intervention group (IG)/ control group (CG)	Who did conduct the intervention?	Concept	Results/Conclusion[2]
Phytotherapy		with a 1:1 allocation		gastro-intestinal, gynaecological, haematolo-oncology, head and neck, lung, neurology, sarcoma, skin, urology	No CG.	was booked with one of four participating therapists all experienced in delivering both interventions.	essential oils to promote and enhance the health and well-being of the individual (IFPA).<br>**M** involves the administration of combinations of specific physical manipulations applied in a systematic way.<br>**R** is a therapeutic method that uses manual pressure applied to specific areas, or zones.	statistical difference between groups<br>for MYCaW second concerns or overall well-being scores, proportions of patients gaining clinical benefit.<br>____________________<br>Overall: Results demonstrate clinical benefit for both interventions for each outcome, but cannot establish superior of one intervention over the other for the second MYCaW concern, overall wellbeing or for VAS R.
Phytotherapy	Gok Metin et al. 2017 [47]<br><br>Ankara, Turkey	Open-label RCT	Douleur Neuropathique questionnaire, Visual Analog Scale (VAS), Neuropathic Pain Impact on Quality of Life questionnaire.	Patients with diabetic neuropathy (n=46)	IG: (n = 21) received **Aromatherapy-Massage (AT-M)** three times per week for a period of 4 weeks.<br><br>CG: (n = 25) received only routine care.	Nursing intervention<br><br>Training and experience of nurses in **AT-M** is critical to achieving positive results.	**AT** is defined as the use of essential oils, extracted from plants, to increase QoL and well-being. Chemical components of essential oils pass through the nasal passages to the olfactory system and the limbic system of the brain. The amygdala and hippocampus are particularly important sites in the limbic system for the processing of essential oils.	Neuropathic pain scores significantly decreased in the intervention. Similarly, QoL scores significantly improved in the IG in the fourth week of the study.<br>____________________<br>Overall: **AT-M** is a well-tolerated, feasible, and safe nonpharmacological method that can be readily integrated into clinical settings by nursing staff.
Manipulative therapies	Kutner et al. 2008 [49]<br><br>Aurora, Colorado, USA	RCT Prospective, single-blind, multi-site randomized trial.	Immediate Memorial Pain Assessment Card (MPAC), Brief Pain Inventory (BPI)Neuropathy Pain Scale (NPS), .	380 adults with advanced cancer experiencing moderate-severe pain; 90% were enrolled in hospice	IG (n = 188): Six 30-minute **Massage** (**M**) over two weeks (effleurage, petrissage)<br><br>CG (n = 192): Simple touch sessions	Licensed **M**-therapists, at least 6-month experience treating advanced cancer or hospice	**M** may interrupt the cycle of distress through therapist intentionality (presence, communication and desire to produce a therapeutic	Both groups demonstrated statistically, but not clinically, significant sustained improvements in pain (BPI).<br><br>There were no statistically or clinically significant differences

[1] The number in brackets refer to the comprehensive reference list (see full article).
[2] A list of all abbreviations can be found at the end of Table S2.

Supplementary S4.

**Treatments**	**Study[1]/ Country (cultural context)**	**Study design**	**Outcome measures (Patient-reported and others)**	**Patients (gender, tumor entity)**	**Intervention group (IG)/ control group (CG)**	**Who did conduct the intervention?**	**Concept**	**Results/Conclusion[2]**
						patients, 500-hour program in **M**.	response), induction of a relaxation **(Rel)** response, increased blood and lymphatic circulation, potentiation of analgesic effects, decreased inflammation and edema, manual release of muscle spasms, increased endogenous endorphin release and competing sensory stimuli that override pain signals.	between study arms in sustained outcome pain measures.<br>____________________<br>Overall**: M** may have immediately beneficial effects on pain and mood among patients with advanced cancer. Given the lack of sustained effects and the observed improvements in both study arms, the potential benefits of attention and simple touch should also be considered in this population.
Manipulative therapies	S3 guideline complementary medicine in the treatment of oncology patients,<br>Nov 2020 [63]<br><br>Jane et al. (2011) - Taichung City, Taiwan;<br>Wilkie et al. (2000) San Francisco, USA;<br>Toth et al. (2013) - Boston, USA;<br>Taylor et al. (2003) Charlottesville, USA;<br>Kutner et al. (2008) - Denver, USA;<br>Mehling et al (2007), San Francisco, USA;<br>Rosen et al. (2013) - Boston, USA;<br>Collinge et al. (2013) - Eugene, USA;	9 RCTs	Numeric Rating Scale (NRS)	Patients with bony metastases, persons with advanced cancer, cancer patients undergoing surgery, cancer patients receiving chemotherapy, women with cancerous lesions patients with metastatic cancer, cancer patients undergo surgery	IG: Swedish **Massage** (**M)**, therapeutic **M**<br><br>CG: **Healing Touch (HeTo),** simple touch,<br>vibration therapy	Certified **M**-therapists	**M** describes one of the best known and oldest healing methods. **M** uses mechanical techniques of touch that are intended to influence the muscles and connective tissue. For cancer patients, it is intended to reduce anxiety and depression, alleviate physical symptoms.	2 studies found no effect, 2 studies found only a short-term effect, and one study found a longer-term effect.<br><br>The available studies do not indicate an added value of M compared to other touch-intensive interventions.<br>____________________<br>Overall: There are heterogeneous data from RCTs on the effectiveness of Swedish M in reducing pain in oncology patients. No recommendation can be made for or against the use of Swedish **M** to reduce pain in these patients.

[1] The number in brackets refer to the comprehensive reference list (see full article).
[2] A list of all abbreviations can be found at the end of Table S2.

Supplementary S4.

Treatments	Study[1]/ Country (cultural context)	Study design	Outcome measures (Patient-reported and others)	Patients (gender, tumor entity)	Intervention group (IG)/ control group (CG)	Who did conduct the intervention?	Concept	Results/Conclusion[2]
	Post-White et al. (2003) - Minneapolis, USA							
Manipulative therapies	Listing et al. 2009 [83]<br><br>Berlin, Germany	RCT	Short Form-8 Health SurveyTM (bodily pain), European Organization of Research and Treatment of Cancer quality of life questionnaire breast module (EORTC QLQ-BR23), Giessen Complaints Inventory (GBB), Berlin Mood Questionnaire (BSF)	Adults (n=86) with breast cancer. Tumor size pT2 (p5 cm), nodal state pN2 (p9 tumor positive axillary nodes), no distant metastases, disease onset p4 years, and time since last chemotherapy and/or radiation therapy 43 months	IG (n = 50)**:** Classical **Massage** (**M)** with rose or calendula oil. For a period of 5 weeks, the IG group received bi-weekly 30-min classical **M** in the back and head–neck areas.<br><br>CG (n = 36)**:** Routine health care	Trained licensed female **M**-therapists.	Pain reduction through **M** is explained by desensitization of nociceptors. The manipulation of muscles and fascia induces local biochemical triphosphate) and can increase the local blood changes release of neuropeptides as calcitonin-related peptides). These local changes may influence neural activity at the associated segmental level of the spinal cord, and may modulate the activities of the subcortical nuclei and the limbic system, which influence pain perception.	Significantly higher reduction of physical discomfort in IG at T2 and at T3.<br><br>Decrease in fatigue. Significant reductions by univariate comparisons for three of the four components of physical discomfort: bodily pain, pain of limbs, and breast symptoms.<br><br>The effect of treatment on mood disturbances was significantly higher if a patient was treated continuously by the same masseur.<br>________________________<br>Overall: Classical **M** seems to be an effective adjuvant treatment for reducing physical discomfort and fatigue, and improving mood disturbances in women with early stage of breast cancer.
Manipulative therapies	Cunningham et al. 2011 [74]<br><br>South Carolina, Charleston, USA	Case report	MD Anderson Symptom Inventory	45-year-old African–American male with a 14-year history of gastroesophageal reflux. He initially presented to cancer center with a 6-month history of epigastric discomfort and dysphagia with solids.	After about 8 months with CIPN in all extremities,<br>the patient began<br>a 6-week course of **Manual therapy (classical massage)** (**MaT)** given in three sessions<br>per week.<br><br>Treatment was<br>administered as a combination of effleurage (stroking with a light, soothing touch) and petrissage (deep kneading and wringing in a	Two licensed massage-therapists	The mechanisms by which **MaT** may impact CIPN are not understood. **M** is reported to induce positive short and longer-term changes in circulatory measures in the extremities of type 2 diabetes. Research is	Before first **MaT**: pain was rated as 4 and numbness and tingling as 10.<br><br>End of treatment: pain decreased to 0 in all extremities. Numbness decreased to 0, tingling decreased to 1.<br><br>Three weeks after completion of **MaT**, CIPN symptoms were stable and unchanged since his last treatment.<br>________________________

[1] The number in brackets refer to the comprehensive reference list (see full article).
[2] A list of all abbreviations can be found at the end of Table S2.

Supplementary S4.

Treatments	Study[1]/ Country (cultural context)	Study design	Outcome measures (Patient-reported and others)	Patients (gender, tumor entity)	Intervention group (IG)/ control group (CG)	Who did conduct the intervention?	Concept	Results/Conclusion[2]
				While on induction chemotherapy, he developed symptoms of neuropathy (tingling, tight feeling in the pads beneath the toes).	downward and outward motion) on each extremity (12. 5 minutes).		needed to understand the role of **MaT**, a nontoxic, non-pharmacologic intervention, in amelioration of CIPN and to determine whether improvements in CIPN symptoms are related either directly or indirectly to changes in blood flow. Ankle brachial index and systolic and diastolic pressures	Overall: MaT (classical Massage) was associated with greatly reduced CIPN symptoms from grade 2 to 1 and markedly improved quality of life.
Manipulative therapies	Boyd et al. 2016 [51]<br><br>Rochester, Minnesota, USA	Systematic review and meta-analysis (16 RCTs)	Functional outcomes related to pain: pain intensity/severity, activity, stress, mood (anxiety), sleep (fatigue), and HrQoL	In the 16 studies, 31.5% were male and 68.5% were female with the mean age of 57.2 across the studies	**Masssage (M)** techniques named as **M**, **M** therapy, Thai-**M**, therapeutic **M**, and lymphatic drainage were compared to several types of control/comparator arms including no treatment, standard care	Most providers were **M** therapists (n = 8) or some type of unspecified therapist (n = 3). Other types of providers included a nurse (n = 2), healing-arts specialist (n = 1), caregiver (n = 1), and a researcher trained in **M** (n = 1).	A large number of cancer patients are seeking out and utilizing **Complementary and Integrative Medicine (CIM)** therapies. In fact, **M** can help with not only physical relaxation **(Rel)** and relief from physical pain but also, emotional distress, functional ability, and overall quality of life.	12 high and 4 low quality studies were included.<br><br>**M**: effective for treating pain compared to no treatment active comparators. Compared to active comparators. **M**: beneficial to treat fatigue.<br><br>Effects on physical function and sleep were small and not significant.<br>________________________<br>Overall: Weak recommendations are suggested for **M**, compared to an active comparator, for the treatment of pain, fatigue, and anxiety. No recommendations were suggested for **M** therapy compared to no treatment or sham control based on the available literature to date.
Manipulative therapies	Pan et al. 2013 [58]<br><br>Lanzhou, China	Systematic review and meta-analysis (18 RCTs involving a total of 3889 patients)	Treatment-related symptoms (pain, fatigue, sleep disturbances, gastrointestinal symptoms and/or negative mood)	Female patients aged 18 years or older with a history of breast cancer receiving active breast	IG**:** Different Styles of **Massage (M)** used varied among all RCTs. Program length and intensity varied, ranging from 75- to 90-min, weekly over 3 weeks to 6 months.	Not reported	**M** is one of the oldest of the healing arts and offers a drug-free, non-invasive and humanistic approach working	18 studies with a total of 950 participants were included. Meta-analysis revealed a significant improvement in pain by **M**.

[1] The number in brackets refer to the comprehensive reference list (see full article).
[2] A list of all abbreviations can be found at the end of Table S2.

Supplementary S4.

Treatments	Study[1]/ Country (cultural context)	Study design	Outcome measures (Patient-reported and others)	Patients (gender, tumor entity)	Intervention group (IG)/ control group (CG)	Who did conduct the intervention?	Concept	Results/Conclusion[2]
				cancer treatments	CG**:** Self-initiated support without any treatment, usual health, health education, ten 20-min visits, modified **M** treatments without energy balancing features, multi-layered compression bandaging, self-administered treatment.		in concert with the body's own ability to heal itself. Chinese medicine (**CH**) highlights a method of manipulating the soft tissues of the body using pressure and traction to enhance function, aid in the healing process, decrease muscle reflex activity, inhibit motor-neuron excitability, and promote relaxation and well-being.	Patients receiving regular use of **M** had significantly greater reductions in anger and fatigue symptoms.<br><br>No significant differences in depression, anxiety, pain, upper limb lymphedema, cortisol and health-related quality of life.<br>________________________<br>Overall: Evidence demonstrates that there was mild evidence that **M** may be a useful intervention in alleviating negative emotions and fatigue in patients with breast cancer.
Manipulative therapies<br><br>Mind-body therapies	Dikmen & Terzioglu 2019 [45]<br><br>Konya, Turkey	Single-blind, randomized controlled study consisting of three IG and one CG	Brief Pain Inventory (BPI), The Brief Fatigue Inventory (BFI), Multidimensional Quality-of-Life Scaled Cancer (MQOLS-CA)	Participants (n = 80) included those diagnosed with uterine, ovarian, and cervical cancers at grades I-III and treated with the second or third cycle of chemotherapy	4 groups (<br><br>- **Reflexology (R) (n =20)**<br>- **Progressive muscle re laxation**(**PMR) (n = 20)**<br>- **R** + **PMR (n = 20) over 8 weeks**<br>- Control (n = 20)	Conducted by medical/nursing researchers	**R** interventions prevent obstructions and refresh bodies by dissolving the accumulation of lactic acid as microcrystals on foot, introducing them into blood circulation and allowing blood to run freely in veins. Therefore, **R** interventions are considered to decrease fatigue severity in cancer patients.	In **R** and **R** + **PMR** groups, a significant decrease in pain severity. In the PMR alone group, pain severity decreased significantly.<br>________________________<br>Overall: **R** and **PMR** are recommended to be performed together by oncology nurses in a safe way to help gynecologic cancer patients coping with adverse effects of chemotherapy, such as pain and fatigue symptoms.
Manipulative therapies	Ernst 2009 [72]<br><br>Exeter, UK	Systematic Review (18 RCTs including in total 949 patients	Treatment-related symptoms (e.g., pain, sleep quality, anxiety)	Patients with any medical condition	IG: **Reflexology (R)** treatment<br><br>CG: Placebo or conventional care alone	Trained reflexologists	**R** is based on the belief that the whole body is represented on the foot (mostly the soles of the feet), and that the	18 RCTs included. 3 RCTs considered cancer palliation:<br><br>First study: greater improvement of QoL (but not for pain measured with the VAS) in the IG after

[1] The number in brackets refer to the comprehensive reference list (see full article).
[2] A list of all abbreviations can be found at the end of Table S2.

Supplementary S4.

Treatments	Study[1]/ Country (cultural context)	Study design	Outcome measures (Patient-reported and others)	Patients (gender, tumor entity)	Intervention group (IG)/ control group (CG)	Who did conduct the intervention?	Concept	Results/Conclusion[2]
							internal organs can be stimulated by pressing particular areas of the foot (less commonly the hands).	cancer patients have received 3-foot **R** sessions<br><br>Second study: 1 session of **R** for breast and lung cancer patients significantly reduces anxiety (but not pain measured with the VAS)<br><br>Third study: no difference in the reduction of pain and anxiety compared to placebo after patients received 6 **R** sessions<br>______<br>Overall: Evidence does not demonstrate convincingly that **R** is an effective treatment for any medical condition.
Manipulative therapies<br><br>Rhythmical embrocations	Post-White et al. 2003 [87]<br><br>Minneapolis, Minnesota, USA	RCT, randomized, prospective, 2-period, crossover intervention study	Brief Pain Inventory (BPI), Brief Nausea Index (BNI), Profile of Mood States (POMS)	Patients (n=230) ≥18 years receiving chemotherapy and pain, nausea, or fatigue rated 3 or more on a scale of 0 to 10	IG**:** Therapeutic-**Massage (M) (n = 78)** and **Healing touch (HeTo) (n = 77)** (4weekly 45-minute sessions)<br><br>CG (n = 75)**:** Caring presence alone<br><br>Control Condition for both groups was standard cancer treatment alone	Certified and credentialed **M** and **HeTo** practitioners who also were registered nurses.	Therapeutic-**M** involves rhythmic and methodical stretching and compressing of the muscles and connective tissue through touch of the therapist's hands, with the benefit of increasing circulation, stimulating venous and lymphatic drainage, improving muscle tissue metabolism and elasticity, and promoting relaxation **(Rel)** through enhanced parasympathetic and reduced sympathetic nervous system activity.	Therapeutic-**M** and **HeTo** are more effective than presence alone or standard care in reducing pain, mood disturbance, and fatigue in patients receiving cancer chemotherapy.<br><br>No significant changes (all interventions) over time between groups on pain index or interference in living due to pain. Subjects receiving **M**, used less NSAID medications during the **M** than during the control period.<br><br>Significant decrease in total mood disturbance for all the treatment over time in comparison to CG.<br>______<br>Overall: Participants rated both interventions highly regarding overall helpfulness and satisfaction. There was no clear benefit of one intervention over the other.

[1] The number in brackets refer to the comprehensive reference list (see full article).
[2] A list of all abbreviations can be found at the end of Table S2.

Supplementary S4.

Treatments	Study[1]/ Country (cultural context)	Study design	Outcome measures (Patient-reported and others)	Patients (gender, tumor entity)	Intervention group (IG)/ control group (CG)	Who did conduct the intervention?	Concept	Results/Conclusion[2]
							**HeTo** is an energy therapy that involves the use of the practitioner's hands above and on the patient's body. The goal of **HeTo** is to restore harmony and balance in the energy system to help the person selfheal.	
Manipulative therapies	Stephenson et al. 2003 [77]<br><br>Greenville, North Carolina, USA	RCT	0 to 10 self-report pain scale recommended by Joint Commission on the Accreditation of Healthcare Organizations	Patients (n=36) on an oncology unit with metastasis	IG (n=19): **Foot-reflexology (FR)** was delivered two times, 24 hours apart<br><br>CG (n=17): no intervention.	Foot reflexologist from the International Institute of **reflexology (R)** using the Original Ingham method	Not described	Pain scores were lower by 2.4 more points in the IG than in the CG immediately after intervention.<br>___<br>Overall: no statistically significant effect at 3 hours after intervention, no significant effect at 24 hours after intervention.
Manipulative therapies	Wyatt et al. 2017 [78]<br><br>East Lansing, Michigan, USA	RCT	M.D. Anderson Symptom Inventory, Quality of Life Index (QLI), Multidimensional Scale of Perceived Social Support (MSPSS)	Patients (n=256) with breast cancer stage III or IV, undergoing Chemotherapy, target and/or hormonal therapy.	IG (n=128): Reflexology **(R)** with a duration of 15 minutes per foot, totaling a 30-minute session over four weeks.<br><br>CG (n=126): no intervention	Friends/family caregiver<br>A study reflexologist to train the family caregivers	Reflexes in feet correspond to organs, glands and system of the body	Significant reductions in summed symptom severity in the IG compared with attention control.<br>___<br>Overall: immediate positive effect of **FR** for patients with metastatic cancer who report pain.
Rhythmical embrocations	Giasson & Bouchard 1998 [81]<br><br>Montréal, Quebec, Canada	RCT	Well-being scale (validated scale based on Edmonton Symptom Assessment System, Visual Analog Scale (VAS) Scale)	Terminal cancer patients (n=20) between 18-70 years. French speaking, no symptoms of confusion. Palliative Care/ terminal care	IG (n = 10): Three non-contact **therapeutic touch (TT)** treatments on consecutive days using a protocol., Duration: 15-20 min.<br><br>CG (n = 10): Rest period, same duration, using a protocol.	Nurse with experience and supervised instruction in **TT**	Influencing energy fields without physical contact. Based on Roger`s nursing conceptual model.	Higher sensation of well-being in IG. Significant difference in the mean progression of well-being.<br><br>The results support the hypothesis that **TT** increase sensation of well-being in persons with terminal care.<br>___<br>Overall: The investigators experience in **TT** is a key issue. Limitation: Small sample. Data collection and study interventions (IG and CG) by the same person = author.

[1] The number in brackets refer to the comprehensive reference list (see full article).
[2] A list of all abbreviations can be found at the end of Table S2.

Supplementary S4.

Treatments	Study[1]/ Country (cultural context)	Study design	Outcome measures (Patient-reported and others)	Patients (gender, tumor entity)	Intervention group (IG)/ control group (CG)	Who did conduct the intervention?	Concept	Results/Conclusion[2]
Manipulatives therapies	Jane et al. 2008 [103]<br><br>Kwei-Shan, Tao-Yuan, Taiwan	Systematic review (7 quasi-experimental studies, 7 experimental studies 1 study with unknown design including in total 1943 patients	Treatment-related symptoms (e.g., pain, fatigue, mood, anxiety, depression).	Adult cancer patients	**Massage (M) types**: light Swedish **M**, full-body **M** with diverse stroking techniques (ie, petrissage, friction, and compression), back **M**, slow stoke back **M** (SSBM), foot **M**, and lymphatic draining	To ensure fidelity in delivering the **M**, most investigators either used one therapist or had a spot or manipulation check.	**M** is a systematic and scientific manipulation of soft tissues of the body. **M** therapy has been extensively documented as one of the oldest therapeutic approaches used to reduce pain, anxiety/disturbed mood, agitation, physiological arousal, sleep disruption, and muscle tension.	15 studies included. Effects of **M** are inconsistent, partially due to methodological issues related to less rigorous inclusion criteria, failure to consider potential confounding variables, inconsistent **M** doses and protocols, less than rigorous research designs, measurement errors, and inadequate statistical power.<br>___<br>Overall: Despite methodological flaws, trends suggest that **M** appeared to have an immediate or short-term (5-20 minutes) effect on improving pain intensity, nausea, fatigue, distressing symptoms, anxiety, self-report of relaxation (Rel), and physiological arousal.
Manipulatives therapies	Kim et al. 2010 [99]<br><br>Daejeon, Republic of Korea (South Korea)	Systematic review (1 RCT, 3 CCTs), in total n=281 patients	Nausea, physical or psychological symptom, and quality of life.	Adult breast cancer patients	IG: Any type of **Reflexology (R)** 20 – 60 min<br><br>CG: Any type of active control, a no treatment group or a placebo group)	Not reported	Not described	1 RT, 3 CCTs were included. One RCT showed significant differences in quality of life and mood when **R** was compared with self-initiated support.<br><br>Three CCTs tested **R** compared with no treatment or simple rest. All of them suggested favorable effects of **R** on pain, nausea, and vomiting.<br>___<br>Overall: Insufficient evidence for the effectiveness of **R** as a symptomatic treatment for breast cancer. The risk of bias in the primary data that exists is high.
Manipulative therapies	Grealish et al. 2000 [105]<br><br>Canberra, Australian Capital Territory, Australia	A quasi-experimental research design was used.<br><br>.	Self-assessment of pain (VAS 0-100), subjective experience of pain, nausea and relaxation.	Participants (n=87), (52 women and 35 men) between 18 to 88 years, primary cancer sites varied, with 32 subjects	**Foot-massage (FM)** sessions of 10 minutes duration. The participants were asked to position themselves comfortably in their beds, or they were assisted with this procedure. The experiment proceeded on three consecutive evenings between 7 and 8 p.m.	To ensure consistency in method, only two research nurses administered **FM**. Both nurses, trained	**FM** induces relaxation through the acts of touch. Once the subject is in a relaxed state, the subjective perceptions of pain	Pain levels decreased significantly during **M** treatment.<br><br>There also was evidence that **M** reduces feelings of nausea.

[1] The number in brackets refer to the comprehensive reference list (see full article).
[2] A list of all abbreviations can be found at the end of Table S2.

Supplementary S4.

Treatments	Study[1]/ Country (cultural context)	Study design	Outcome measures (Patient-reported and others)	Patients (gender, tumor entity)	Intervention group (IG)/ control group (CG)	Who did conduct the intervention?	Concept	Results/Conclusion[2]
				reporting metastatic disease		in the techniques required for the study, were experienced in the administration of **M**.	and nausea are decreased.	No control for other medication, with 37% receiving medications for pain and 19% for nausea.<br>____________________<br>Overall: Generalizability is limited. It has been shown that a simple 10-minute nursing intervention can have an effect on subjective symptoms such as pain and nausea. Further research on **M** as a nursing intervention for patients living with a cancer diagnosis is warranted.
Mind-body therapies<br><br>Acupuncture/ Acupressure	Dy 2010 [95]<br><br>Lutherville, Maryland, USA	Review	Pain	Cancer populations (with the exception of neuropathic pain)	IG: **acupuncture (A)**, **relaxation (Rel)**<br><br>CG: Placebo-**A**	Acupuncturists	Pain is frequently multidimensional; other symptoms, distress, or psychosocial or spiritual concerns can interact with physical pain or impact the effectiveness of pain treatment. A complete whole patient evaluation also includes assessment for other symptoms.	Evidence on **A** and **Rel** is sparse on CIPN in cancer patients.<br><br>**A** for pain in general populations found a very small effect. Moderate effect of placebo-**A**, suggesting that the therapeutic relationship may be an important factor in reducing pain.<br>____________________<br>Overall: Large RCT evidence outside cancer may be applied with caution, given that the pathophysiology of cancer pain is often different than other acute or chronic pain. For some syndromes, such as CIPN, there are studies showing only inefficacy of multiple treatments.
Mind-body therapies	Kwekkeboom 2001 [70]<br><br>Iowa City, Iowa, USA	Secondary analysis using data from women who participated in an RCT including 34 patients of guided **imagery (I)**.	Pain diary (pain intensity, pain distress, pain coping behaviors)	Patients: 18 years or older, breast cancer, gynecologic cancer, Caucasian women with postoperative pain	**Self-management strategies (SMT)**. Pain diary to document pain episodes whenever pain became bothersome. During the initial pain episode, all participants were instructed to take a dose of pain medication. Sixteen persons were randomly assigned to use a guided-**imagery (I)** intervention at the same time that they took their dose of analgesic.	Patients	**SMT** that patients used to help control their pain and cope with pain.	Trend toward more positive affect in the group that used a combination of non-pharmacologic strategies than the group that used only analgesic medications.<br><br>Differences in the other pain-related outcome variables were not significant.<br><br>Techniques used included positioning (Po), distraction (DT),

[1] The number in brackets refer to the comprehensive reference list (see full article).
[2] A list of all abbreviations can be found at the end of Table S2.

Supplementary S4.

Treatments	Study[1]/ Country (cultural context)	Study design	Outcome measures (Patient-reported and others)	Patients (gender, tumor entity)	Intervention group (IG)/ control group (CG)	Who did conduct the intervention?	Concept	Results/Conclusion[2]
								relaxation (Rel), heat application (He), and eating/drinking.<br>___<br>Overall. Nurses may benefit from knowing which **SMT** patients find helpful so that they can encourage their use and teach similar strategies.
Mind-body therapies	Telles et al. 2019 [73]<br><br>Chandigarh, Union territory, India<br><br>Madison, Wisconsin, USA	Review	Neuropathic pain (NP)	Patients with spinal cord injury (SCI) induced NP patients.	IG: **Yoga (Y)**<br>CG: Individually matched controls, not involved in any kind of Y practice	**Y** practitioners, Yogis	Neuropathic pain is a debilitating condition that may result from spinal cord injury (SCI). Like cancer and other disorders, **Y** may promote healing for SCI induced NP, by alleviating pain as it has shown promising results in the other pain disorders.	**Y** could be used in managing SCI induced NP pain by regulating the action of various mechanisms and its associated molecules.<br><br>Modern prescriptive treatment strategies combined with alternative approaches like **Y** should be used in rehabilitation centers and clinics in order to ameliorate chronic NP.<br>___<br>Overall: Practical considerations of careful **Y** as part of an integrative medicine approach for NP associated with SCI are recommended.
Mind-body therapies	Vadiraja et al. 2009 [92]<br><br>Bangalore, India	RCT	Rotterdam symptom checklist (RSCL), Quality of life EORTC QOL 30 C30	Female patients (n=88) with breast cancer	IG (n = 44): **Yoga (Y)** (during 6 weeks, 18 to 24 yoga sessions)<br><br>CG (n = 44): supportive therapy intervention	Trained yoga therapist	Asanas, breathing exercises, meditation **(Me)** and relaxation **(Rel)** techniques	Significant decrease in pain and nausea and vomiting in **Y** group.<br>___<br>Overall: Beneficial findings in managing cancer-related symptoms.
Mind-body therapies	Galantino et al. 2019 [80]<br><br>Galloway, New Jersey, USA	Open-label, single-arm, mixed-methods feasibility trial	Measurement of Sit and Reach, Functional Reach, and Timed Up and Go, Patient Neurotoxicity Questionnaire (PNQ), Functional Assessment of Cancer Therapy—Neurotoxicity (FACT-GOG-NTX), Measurement of biomarkers (salivary	Cancer survivors with CIPN (n = 10)	IG: **Yoga (Y)** and **Meditation (Me)** twice a week for 8 weeks for 1.5 hours, with home program and journaling	Certified **Y** instructors	Research has focused on pharmacological therapies aimed at reducing CIPN pain, which does not treat the loss of sensation and motor weakness.<br><br>CIPN causes balance impairments and	Significant improvements were found in flexibility, balance, and fall risk.<br><br>PNQ improved significantly with other measures improving non-significantly.<br><br>Five themes emerged: (1) vacillation of CIPN pain perception over time; (2) transferability of skills to daily activities; (3) improvement in physical function; (4) perceived

[1] The number in brackets refer to the comprehensive reference list (see full article).
[2] A list of all abbreviations can be found at the end of Table S2.

Supplementary S4.

Treatments	Study[1]/ Country (cultural context)	Study design	Outcome measures (Patient-reported and others)	Patients (gender, tumor entity)	Intervention group (IG)/ control group (CG)	Who did conduct the intervention?	Concept	Results/Conclusion[2]
			cortisol), bio esthesiometer)				leads to changes in elicitability and sensitivity of spinal reflex circuitry associated with postural instability.	relaxation (Rel) as an effect of SYM; and (5) group engagement provided a social context for not feeling isolated with CIPN.<br>Overall: Preliminary data suggest **Y** and **Me** improve QOL, flexibility, and balance in cancer survivors with CIPN.
Mind-body therapies	Doorenbos et al. 2005 [79]<br><br>East Lansing, Michigan, USA	RCT	Interviewers, Physical Symptom Experience Tool,<br>Modified version of the Comorbidity Questionnaire,<br>Center for Epidemiologic Studies Depression Scale	Newly diagnosed cancer patients (n = 237) between 31 to 87 years, the majority of the participants had advanced (stages 3 or 4) cancer (67%).<br><br>The most common site of cancer was breast (38%), followed by lung (35%).	IG (n = 118): **cognitive behavioral intervention (CBI)** 10-contact (5 in person and 5 via telephone), 18-week intervention, and four telephone interviews, occurring at baseline and at weeks 10, 20, and 32.<br><br>Symptoms were matched with specific problem-solving strategies: self-care management, information and decision-making, counseling and support, and communication with providers.<br><br>CG (n = 119)**:** conventional care	Nurse	**CBI** is used to impact symptom limitations. Problem solving is one of several **CBI** approaches used to frame stressful events and drive positive behavioural changes. Four major steps in any **PST** strategy are as follows: problem definition and formulation; generation of alternative solutions; decision making; and solution implementation and verification.	Participants receiving the **CBI** had lower scores of symptoms.<br><br>Younger patients reported more symptom limitations than their older counterparts<br><br>On average, after 10 weeks, the IG reduced symptom limitations by a statistically significant 13 points more than the CG and maintained this advantage over the course of treatment.<br>Overall: **CBI** was key to decreasing symptom limitations. Findings also suggest that nursing interventions may be particularly helpful to younger individuals in managing cancer-related symptom limitations.
Mind-body therapies	Buffart et al. 2012 [52]<br><br>Amsterdam, Netherlands	Systematic literature review and meta-analysis (16 studies thereof 13 RCTs including in total 722 patients)	Psychosocial and physical outcomes	Adults with any cancer diagnosis either during or post treatment	IG: **Yoga Y** including physical postures<br><br>CG: non-**exercise** (**E)** or wait-list	All **Y** classes were led by experienced **Y** instructors.	**Y** is a 'mind-body' **E**, a combination of physical poses with breathing and meditation.	**Y** appeared to be a feasible intervention and beneficial effects on several physical and psychosocial symptoms were reported.<br>Overall: Effect size on functional well-being was small, and they were moderate to large for psychosocial outcomes.
Movement therapies	Expert standard for pain management in nursing (Expertenstandard	Nursing guideline	Pain scales	Adults	For example: **transcutaneous electrical nerve stimulation (TENS)**, **massage (M), aromatherapy (AT), distraction**	Nurses	Not described	Included: 20 guidelines (7 national, 13 international), Cochrane review (17 studies/ 724 participants).

[1] The number in brackets refer to the comprehensive reference list (see full article).
[2] A list of all abbreviations can be found at the end of Table S2.

Supplementary S4.

Treatments	Study[1]/ Country (cultural context)	Study design	Outcome measures (Patient-reported and others)	Patients (gender, tumor entity)	Intervention group (IG)/ control group (CG)	Who did conduct the intervention?	Concept	Results/Conclusion[2]
Mind-body therapies<br><br>Cryotherapy<br><br>Heat applications<br><br>Manipulatives therapies<br><br>TENS/Scrambler therapy<br><br>Other supportive interventions	Schmerzmanagement in der Pflege) 2020 [66]<br><br>Osnabrück, Germany				**therapy (DT), music therapy (MuT)**, TENS versus placebo (sham) TENS, TENS versus usual care, TENS versus no treatment and TENS in addition to usual care versus usual care alone in the management of neuropathic pain			Beneficial effects of **E** in neuropathic pain (NICE 2016). Beneficial effects of **M**, **hot and cold applications** (**He** and **CT**), **DT**, **MuT**, **patient education (PE)**. Unclear evidence for **AT**.<br><br>No evidence of **TENS** efficacy in adults with neuropathic pain (Gibson et al. 2017).<br>____________________<br>Overall: Robust conclusions are not possible because of the limited statements in the included guidelines.
Movement therapies	Fernandes & Kumar 2016 [69]<br><br>Belagavi, Karnataka, India	Single-group pre-post prospective study	mTNS Modified Total Neuropathy Score (mTNS), Berg Balance Score (BBS)	CIPN patients older than 30 years with a mTNS higher than five (n = 25)	Daily **closed kinematic chain exercise CKC-E** on a firm surface for 15 sessions over 3 weeks. Every exercise lasted for 12 minutes.	Patients themselves	CIPN leads often to gait and balance dysfunction. **CKC** have shown increased firing of muscle spindle afferent and improved motion sense. This led to the hypothesis that **CKC** may have an effect on balance.	There was a significant change in values before and after the **E**.<br><br>mTNS score decreased from 13.88 at baseline at 6.5 at the end of intervention. BBS increased from 26 at baseline to 42 after the intervention.<br>____________________<br>Overall: **E** were effective in reducing CIPN symptoms.
Movement therapies<br><br>Mind-body therapies	Kanzawa-Lee et al. 2020 [54]<br><br>Ann Arbor, Michigan, USA	Comprehensive integrative review (7 RCTs, 6 quasi-experimental studies including in total 1037 patients)	CIPN, balance, and fitness	All cancer patients of all age groups who received chemotherapy	Four different types of **exercise** (**E**) were tested in the 13 studies: **Yoga (Y)**, and **E** with **Aerobic** (**Ae**) (n = 7), **strength training (STr)** (n = 9), and **balance training (BT)** (n = 7)<br>components.	All but 4 studies were conducted in a clinical setting. One intervention was delivered by group.	**E** may attenuate CIPN through its influence on blood circulation/oxidative stress, inflammation, pain-inhibiting neurotransmitters, endogenous opioids, growth factors, neuroplasticity, and coping and symptom interaction mechanisms. Although **E** is	13 studies (7 RCTs, 6 quasi-experiments) were reported **E** effects in various adult with CIPN.<br><br>No studies provided high-quality evidence; 2 studies provided moderate-quality evidence.<br><br>All 7 studies with an **Ae E** component led to significant—most studies showing moderate to large—CIPN benefits.<br>____________________<br>Overall: Empirical evidence is insufficient to definitively conclude that **E** interventions ameliorate CIPN. Nurses can

[1] The number in brackets refer to the comprehensive reference list (see full article).
[2] A list of all abbreviations can be found at the end of Table S2.

Supplementary S4.

Treatments	Study[1]/ Country (cultural context)	Study design	Outcome measures (Patient-reported and others)	Patients (gender, tumor entity)	Intervention group (IG)/ control group (CG)	Who did conduct the intervention?	Concept	Results/Conclusion[2]
							recommended for cancer survivors, little is known about specific **E** prescriptions' effectiveness in reducing CIPN and feasibility among individuals who have received neurotoxic chemotherapy.	encourage and educate cancer survivors about the safety and importance (particularly in bolstering balance and fitness and possibly alleviating symptoms) of **E** throughout cancer survivorship.
Movement therapies	Kleckner et al. 2018 [48]<br><br>Rochester, New York, USA	Secondary analysis of a nationwide phase III randomized controlled trial of **E** for fatigue.	Patient-reported CIPN symptoms (numbness, tingling, (hot/coldness in hands/feet, measurement of adherence)	Cancer patients (n = 355, 56 ± 11 years, 93% female, 79% breast cancer) receiving chemotherapy	IG: chemotherapy plus **exercise** (**E**) (60 min) for Cancer Patients (EXCAP©®). EXCAP is a standardized, individualized, moderate-intensity, home-based, six-week progressive **walking (W)** and resistance **E** program.<br><br>CG: chemotherapy	Clinical research associates, with no professional **E** qualifications, received brief training in the delivery of EXCAP by ACSM-certified **E** professionals. EXCAP conformed with ACSM guidelines for **E** prescription.	**E** reduces chronic inflammation, and inflammation appears to play a role in the etiology and treatment of CIPN. **E** changes how sensations from the hands, feet, and rest of the body are processed by the brain, specifically by the thalamus, sensorimotor cortex, and insula, which are all part of interoceptive brain circuitry.	**E** reduced CIPN symptoms of hot/coldness in hands/feet and numbness and tingling compared to the control.<br><br>**E** reduced CIPN symptoms more for patients who were older, male, or had breast cancer.<br>____________________<br>Overall: **E** appears to reduce CIPN symptoms in patients receiving taxane-, platinum-, or vinca alkaloid-based chemotherapy. Clinicians should consider prescribing **E** for these patients.
Movement therapies	McCrary et al. 2019 [84]<br><br>Kensington, New South Wales, Australia	Prospective pilot intervention study, single group pre-post design	Total Neuropathy Score–clinical version (TNSc), EORTC CIPN-20, Functional assessment tools, Disability and quality of life, Neurophysiology	Cancer survivors (n=35), who had completed chemotherapy, with persisting CIPN.	8-week multimodal **E** intervention, 3 weekly sessions. Prescription **of resistance training (RT)**, **balance training (BT)** and **cardiovascular exercises CardE**)	Qualified **E**-physiologist	Not described.	CIPN, dynamic balance, standing balance in eyes open conditions, mobility and quality of life were improved from pre- to post-**E** with no changes over the control period.<br>No changes were observed in sensory or motor neurophysiologic parameters.<br>____________________<br>Overall: 8 weeks of multimodal **E** training can reduce CIPN symptoms and related functional and quality of life deficits in

[1] The number in brackets refer to the comprehensive reference list (see full article).
[2] A list of all abbreviations can be found at the end of Table S2.

Supplementary S4.

Treatments	Study[1]/ Country (cultural context)	Study design	Outcome measures (Patient-reported and others)	Patients (gender, tumor entity)	Intervention group (IG)/ control group (CG)	Who did conduct the intervention?	Concept	Results/Conclusion[2]
								cancer survivors with persistent CIPN.
Movement therapies<br><br>Mind-body therapies<br><br>Other TCM therapies	Mishra et al. 2012 [57]<br><br>Albuquerque, New Mexico, USA	Cochrane Review (40 studies thereof 33 for quantitative analysis including in total 3694 patients)	Health-related quality of life	Adult cancer Patients who had completed active cancer treatment. Some trials included patients currently undergoing treatment.	IG: **exercise (E), strength training (STr)**, **resistance training (RT)**, **walking (W)**, **cycling (cy)**, **Yoga (Y), Qi Gong (QG),** or **Tai Chi (TC)**<br><br>CG**:** Usual care or other non-**E** intervention	Physiologists, sports trainers, **Y** instructors, or other professionals	Not described	40 trials (38 RCT, 2 CCTs), 3694 participants included<br><br>**E** interventions resulted in decreased anxiety at 12 weeks' follow-up, fatigue at 12 weeks' and between 12 weeks' and 6 months' follow-up, and pain at 12 weeks' follow-up.<br><br>Positive trends and impact of **E** intervention existed for depression and body image<br><br>**E** may have beneficial effects on HRQoL and certain HRQoL domains including cancer-specific concerns (e.g. breast cancer), body image/self-esteem, emotional well-being, sexuality, sleep disturbance, social functioning, anxiety, fatigue, and pain at varying follow-up periods.<br>———<br>Overall: Positive results must be interpreted with caution owing to the heterogeneity of **E** programs tested and measures used to assess HRQoL and HRQoL domains, and the risk of bias in many trials.
Movement therapies<br><br>Manipulative therapies	Schönsteiner et al. 2017 [89]<br><br>Ulm, Germany	Randomized exploratory phase 2 study	Functional Assessment of Cancer Therapy/ Gynecologic Oncology Group neurotoxicity subscale (FACT/GOG-NTX), EORTC QLQ-C30 Quantitative sensory testing (QST)	Cancer patients (n = 131; 18 – 70 years), CIPN grade II–III according to National Cancer Institute Common Toxicity Criteria (NCI CTC, version 3.0) and pathological chair-rising test	IG (n = 66): **Whole-body vibration (WBV) including** .Massage **M, passive mobilization PM and** physical **exercise** (**E**)<br><br>CG (n = 65): standard care	Not described	Not described	During the treatment course the proportion of patients with a normal CRT significantly increased to 19% (17% standard, 20% experimental) after 8 sessions, 51% (56% standard, 46% experimental) after 15 sessions and 62% (56% standard, 68% experimental) at follow-up without significant differences between the two arms at the respective time points.<br>———

[1] The number in brackets refer to the comprehensive reference list (see full article).
[2] A list of all abbreviations can be found at the end of Table S2.

Supplementary S4.

Treatments	Study[1]/ Country (cultural context)	Study design	Outcome measures (Patient-reported and others)	Patients (gender, tumor entity)	Intervention group (IG)/ control group (CG)	Who did conduct the intervention?	Concept	Results/Conclusion[2]
				(CRT) [28] (≥10 s) were eligible.				Overall: Reduction of time needed to complete the CRT from baseline to follow-up assessment was significantly higher in the IG the treatment of CIPN with a program including **M**, mobilization as well as physical **E** and **WBV** had a significantly and clinically relevant beneficial impact on symptoms relieve, physical fitness and sensory function.
Movement therapies	Schwenk et al. 2016 [90]<br><br>Tucson, Arizona, USA	Single blinded, randomized controlled pilot study	VPT score, Numeric Rating Scale (NRS), neuropathy-related numbness in feet (NRS score)), Short-Form Health Survey (SF-12), Falls Efficacy Scale-International (FES-I)], body mass index (BMI), history of fall	Cancer patients (n=22), age ≥ 55 years, CIPN	IG (n =11): B: interactive motor adaptation **balance training** (**BT)** program based on wearable sensors for improving balance 2 sessions per week (45min) four 4 weeks.<br>The training sessions included: (1) ankle point-to-point reaching tasks and (2) virtual obstacle crossing tasks (described below). The balance **E** included repetitive, error-dependent forward/backward/sideward/diagonal weight shifting tasks (with the feet in place) and cognitively challenging dynamic weight transfer tasks (obstacle crossing) designed to improve postural balance<br><br>CG (n = 11): normal exercise program	Supervisor	Interactive game-based balance training program	Significant reductions in postural sway parameters in challenging semi-tandem position.<br><br>No significant changes were noted for balance with “eyes closed”, gait speed, and fear of falling.<br>______________________<br>Overall significant reductions in postural sway in the IG after 4 weeks of training during balance assessments with large effect sizes in the “eyes open” condition.
Movement therapies	Streckmann, Zopf et al. 2014 [60]<br><br>Cologne, Germany	Systematic Review (10 RCTs, 8 controlled clinical trials including 841 patients)	Side effects of PNP.	Patients with with peripheral neuropathy (PNP), independent of the derivation	Various types of **exercise (E)** interventions.	Not described	**E** can attenuate motor deficits induced by PNP: **sensorimotor training** (**SM**), **whole-body vibration** (**WBV**) or **Tai Chi** (**TC**) address small and large sensory nerve fibers.	18 studies were included (10 RCTs, 8 CCT). One study on cancer patients.<br><br>Only one RCT assessed the effects of **E** intervention **(SM, endurance training (EN) and resistance training (RT))** in patients with CIPN. Number of patients with reduced deep sensitivity could be diminished significantly in the IG by 87.5 %,

[1] The number in brackets refer to the comprehensive reference list (see full article).
[2] A list of all abbreviations can be found at the end of Table S2.

Supplementary S4.

Treatments	Study[1]/ Country (cultural context)	Study design	Outcome measures (Patient-reported and others)	Patients (gender, tumor entity)	Intervention group (IG)/ control group (CG)	Who did conduct the intervention?	Concept	Results/Conclusion[2]
								while no changes (0 %) were observed in the CG.<br><br>Patient's quality of life as well as their level of activity were also improved significantly. No adverse events occurred.<br>________________________<br>Overall: **E** is feasible, safe, and effective for neuropathic patients. Balance training (**BT**) has the potential to improve sensory and motor symptoms in PNP, while in PNP of metabolic etiology, **EN** can prevent the onset and delay the progression of PNP.
Movement therapies	Tofthagen et al. 2012 [96]<br><br>Tampa, Florida, USA	Literature Review (10 RCTs, 1 single-arm study, 1 cross-over-study,1 quasi-experimental study including in total 935 patients	Neuropathy symptoms, strength, balance	Patients with peripheral neuropathy or high-risk peripheral neuropathy are focused on cancer	**Strength training (Str)** and **balance training (BT) that** focus on community-dwelling adults and examined falls, fall risk, balance, and/or strength as outcome measures	Not described	**STr** and **BT** programs may increase blood supply to peripheral nerves, increase oxygen delivery to mitochondria, and also increase glucose delivery to mitochondria.	13 studies were included: 2 RCTs = **BT** and **STr**, increased walking speed, and decreased fear of falling in participants in a 60-minute, twice a week for 12 weeks, strength, balance, and functional training program. The training program was feasible and safe for patients with peripheral neuropathy.<br><br>Although few differences in balance, muscle strength, fall, or fear of falling were identified, the intervention was determined to be safe and well tolerated in patients with diabetes with peripheral neuropathy.<br>________________________<br>Overall: Several studies described risk for postural instability, falls, and fall-related injury in patients with CIPN and recommended physical therapy (**PT**) as a treatment option, but no studies were identified that evaluate **STr** and **BT** for treatment of CIPN.
Movement therapies	Verhulst et al. 2015 [97]	Review (1 pilot study, 1 case	Strength, balance and pain	Adults with diabetic	Clinical trials on **whole-body vibration (WBV)** interventions,	Not described	The mechanical oscillations cause	Significant reduction of pain in two studies. The search suggests

[1] The number in brackets refer to the comprehensive reference list (see full article).
[2] A list of all abbreviations can be found at the end of Table S2.

Supplementary S4.

Treatments	Study[1]/ Country (cultural context)	Study design	Outcome measures (Patient-reported and others)	Patients (gender, tumor entity)	Intervention group (IG)/ control group (CG)	Who did conduct the intervention?	Concept	Results/Conclusion[2]
	Maastricht, Netherlands	study , 2 RCTs, 1 single-session study)		peripheral neuropathic or with HIV-associated distal symmetrical polyneuropathy	aimed at improving strength, balance and pain for patients with peripheral neuropathies, which were published until 5th June 2014.		energy to be transferred from the vibrating platform to the lower extremities.	insufficient evidence to assess the effectiveness for the effects of **WBV** on neuropathic pain.<br>Overall: Positive effect of **WBV** on neuropathic pain, but more evidence of high-quality studies including also cancer patients and survivors are needed.
Movement therapies	Zimmer et al. 2018 [94]<br><br>Cologne, Germany	RCT (two-armed, monocentric)	Trial Outcome Index (TOI), Functional Assessment of Cancer Therapy/Gynecologic Oncology Group Neurotoxicity (FACT/GOG-NTX) question- naire	Patients (n=30) with metastasized colorectal cancer	IG (n =17): 8-week multimodal **exercise** (**E**) program, including **endurance (EN)**, **resistance (RT)**, and **balance training** (**BT**), coordination practices (**CoT**).<br><br>CG (n = 13): waitlist control group which received written standard recommendation to obtain physical fitness	Qualified sport therapist	Increased physical activity levels are associated with reduced colorectal cancer risk. The applied intervention counteracts a worsening in neuropathic symptoms and improves balance and strength control.	Regarding CIPN (TOI) there were significant differences between groups in the main analysis.<br>Overall: Beneficial effects for the **E** program.
Movement therapies	S3 Guideline Supportive therapy for cancer patients, Feb 2020 [37]<br><br>AWMF, DKG, and German Cancer Aid<br><br>Streckmann, Zopf et al. 2014 [60]	S3 Guideline		Cancer patients	Non-drug methods	Not described	Not described	Data on prophylaxis and treatment of CIPN with non-drug interventions is limited. **Sensorimotor training (SM)** and possibly also **whole-body vibration (WBV)** represent new options in the context of non-drug therapy of CIPN.<br><br>Clear evidence of improvement of functional limitation through non-medicinal procedures such as sports therapy (**SpT**), occupational therapy (**OT**), physiotherapy (**PhyT**) and physical therapy (**PT**) including electrotherapy (**ET**). Furthermore, there is no evidence for a harmful effect of the intervention.<br>Overall: Expert consensus: in manifested chemotherapy-induced polyneuropathy, exercise (**E**) therapy should be given to

[1] The number in brackets refer to the comprehensive reference list (see full article).
[2] A list of all abbreviations can be found at the end of Table S2.

Supplementary S4.

**Treatments**	**Study[1]/ Country (cultural context)**	**Study design**	**Outcome measures (Patient-reported and others)**	**Patients (gender, tumor entity)**	**Intervention group (IG)/ control group (CG)**	**Who did conduct the intervention?**	**Concept**	**Results/Conclusion[2]**
								improve functionality: This may include: - balance (**BT**) exercises - **SM** e.g. been bath - coordination training (**CoT**) – (**WBV**) – fine motor training (**FMT**).
Movement therapies	S3 Guideline Supportive therapy for cancer patients, Feb 2020 [37]<br><br>Streckmann, Kneis et al. 2014, Freiburg, Germany	RCT (IG n=31, CG n=31)	QOL; movement coordination, endurance, strength and therapy-induced side-effects.	Patients with lymphoma	**Exercise (E)** program. 36-week intervention (**sensorimotor training (SM), endurance training (EN), strength training (Str))** twice a week)	Patients supervised by certified sport- or physiotherapists	Not described	QOL: Significant intergroup difference (ΔT1–T0; P = 0.03). PNP: the average incidence was lower in the IG (12%) than in the CG (27%) (ΔT3–T0; P = 0.07), the symptom diminished in 87.5% of the IG, no patient (0%) in CG showed reduced PNP (P < 0.001). At T3 significantly lower number of patients with PNP in the IG (P = 0.002).<br>_____________________________<br>Overall: Due to the highly significant physiological parameters, the study was terminated prematurely.
Movement therapies	Andersen et al. 2020 [38]<br><br>Manitoba, Winnipeg, Canada	Single-blind (blinded assessor) randomized controlled exploratory study	Patient questionnaires, quantitative sensory testing	Patients during and after chemotherapy (n=48)<br><br>Tumor: Female, Stages I to III patients with breast cancer	IG (n = 22): **Physical therapy** (**PT**) = four visits with a physical therapist to develop a nerve-specific home exercise **(E)** and education program were provided to the treatment group at the beginning of chemotherapy<br><br>CG (n = 26=: treatment as usual	Trained physical therapist	**PT** is well established in orthopaedics and plastic surgery for entrapment neuropathies, neuropathic pain, postoperative nerve repair, and regeneration. Due to the success of nerve gliding treatment in orthopaedic therapy, it was hypothesized that **physiotherapy (PhyT)** intervention prior to or during chemotherapy could improve the sensory symptoms of CIPN.	IG showed strong trends toward less pain and pain decreased over time. Pain pressure thresholds were improved in the treatment group.<br>In the nonrandomized subgroup analysis, participants reporting general **E** had preservation of vibration) and normal heat pain thresholds compared with more sedentary participants.<br>_____________________________<br>Overall: **PT** may improve CIPN pain in the upper extremity for patients with breast cancer, and general **E** throughout chemotherapy treatment was observed to have correlated to preservation of sensory function. It was chosen to measure the hands even though CIPN is a bilateral polyneuropathy affecting hands and feet.

[1] The number in brackets refer to the comprehensive reference list (see full article).
[2] A list of all abbreviations can be found at the end of Table S2.

Supplementary S4.

Treatments	Study[1]/ Country (cultural context)	Study design	Outcome measures (Patient-reported and others)	Patients (gender, tumor entity)	Intervention group (IG)/ control group (CG)	Who did conduct the intervention?	Concept	Results/Conclusion[2]
Nutritional therapy	S3 guideline complementary medicine in the treatment of oncology patients, Nov 2020 [63]<br><br>Hershman et al. (2013) –New York, USA<br>Mondal et al. (2014) – Bengal, India<br>Sun et al. (2016) – Shanghai, Shanghai	1x double-blind multicenter trial<br><br>Single institutional prospective multi-arm randomized study<br><br>Multicenter randomized double-blind and placebo-controlled phase II clinical trial	Evaluation of safety and efficacy, nerve conductive electrophysiological examination velocity (NCV), etc.	Patients with breast cancer<br><br>Patients with lung or breast cancer<br><br>Grade ≥3 neuropathy, while receiving chemotherapy	IG: 3,000 mg per day (six capsules) of **Acetyl-l-carnitine (ALC)**<br><br>IG: **ALC** 250 mg OD from day 1 to day 7 in each 6 cycle of CT)<br>3 g/day **ALC** orally for 8 weeks (2016)<br><br>CG: placebo capsule contained 600 mg of cellulose, randomized to another treatment arm	Patient himself	**AIC** serves as an intracellular energy store, stabilizes cellular membranes, is a radical scavenger and has protective effects on mitochondria. Studies on cancer patients showed that the level of carnitine decreases during chemotherapy, which is why the effect of additional administration of carnitine is being tested.	Hershman et al. was able to find worsening or in-creased peripheral neuropathy with **AIC** administration.<br><br>The study by Mondal et al. 2014 showed no improvement in peripheral neuropathy symptoms or Sun et al. 2016 found an improvement in neurotoxicity.<br>___<br>Overall: Studies included in this recommendation provide very different results. Further limitations: small number of studies and methodological deficiencies.
Nutritional therapies	S3 guideline complementary medicine in the treatment of oncology patients, Nov 2020 [63]<br><br>Mondal et al. (2014) – Bengal, India	RCT (4-armed)	Sensory, motor and pain symptoms	Bronchial cancer, breast cancer and ovarian cancer	IG: Methylcobalamine 500 µg TDS from day 1 of the first cycle to 1 month after completion of CT<br><br>CG: Randomized to another treatment arm (Vit B12, **Vitamin E** (**VE**), **Glutamine (G),** Methylcobalamine	No Information	**VB** is synthesized exclusively by microorganisms and stored in the liver. It plays an important role in various metabolic processes, such as the formation of DNA and the breakdown of fatty acids **(FA)**. **VB** supports blood formation. This leads to the study on the correlation between **VB** and peripheral neuropathy during and after chemotherapy.	Methylcobalamine was superior to **G** and Acetyl-l-carnitine (**AIC**) in relieving sensory, motor and pain symptoms.<br>___<br>Overall: There are no sufficient data from RCTs to assess the efficacy of isolated administration of VB against CIPN in oncological patients.<br><br>No recommendation can be made for or against the use of **VB** in these patients.
Nutritional therapy	S3 guideline complementary medicine in the treatment of oncology	9 RCTs	CTCAE, version and specific gradation scales for oxaliplatin-induced peripheral neuropathy	Different types of cancer patients	IG**:** 2x 300 mg per day, **vitamin E (VE)** during chemotherapy up to 3 months after completion<br>300 mg **VE** each day or<br>400 mg **VE** each day	No information	**VE** exists in different forms, which are summarized under the term	Significant differences between **VE** and control were found in five studies. In four studies in favor of the IG compared with the CG.

[1] The number in brackets refer to the comprehensive reference list (see full article).
[2] A list of all abbreviations can be found at the end of Table S2.

Supplementary S4.

Treatments	Study[1]/ Country (cultural context)	Study design	Outcome measures (Patient-reported and others)	Patients (gender, tumor entity)	Intervention group (IG)/ control group (CG)	Who did conduct the intervention?	Concept	Results/Conclusion[2]
	patients, Nov 2020 [63]<br><br>Afonseca (2013) – Sao Paulo, Brazil;<br>Argyriou (2006a, 2006b) - Rion-Patras, Greece; Kottschade (2011) Sioux City, USA;<br>Mondal (2014) – Bangal, India;<br>Pace (2010) – Rome, Italy;<br>Pace (2003) – Rome, Italy;<br>Salehi and Roayaei (2015) – Isfahan, Iran<br>Shamsaei (2017) – Ahvaz, Iran		CIPN score based on electrophysiological values and self-reported symptoms		(Pace et al. 2003; 2010)<br><br>CG**:** Nothing or<br>Placebo (Pace et al. 2003; 2010)		tocopherols. They are fat-soluble and are found especially in vegetable oils, wheat germ, eggs and nuts. The most important effect of vitamin E in the body is an antioxidant property and the protection of body cells from free radicals.	**VE** leads to comparable CIPN symptom course with patients receiving vitamin **B12 (VB)**, but a significantly better course compared to patients receiving **Acetyl-l-carnitine (AlC)** or **Glutamine (G)**.<br><br>4 studies found no significant differences between Intervention and control<br>________________________<br>Overall: **VE** should not be given in these patients for the prevention and therapy of CIPN.<br><br>Reasons: little information about study population, high drop-out rate - low evidence.
Nutrition al therapy<br><br>Acupuncture, acupressure (TCM)	Greenlee et al. 2017 [42]<br><br>New York, USA	Clinical practice guidelines are based on a systematic literature review of published RCTs from 1990 through 2015.		Breast cancer patients	**Omega-3 (O3), fatty acids (FA), vitamin E (VE), acupuncture (A), electroacupucture (EA), alpha-lipoic acid (ALA), dietary modification (DM)**	Well-trained providers	Implementing **integrative therapies (ITs)** in a clinical setting requires a coordinated team approach. Best practices suggest that providers be trained to the highest standard of their profession and educated in other relevant disciplines.	No A-graded or B-graded therapies reported for the prevention or treatment of CIPN.<br><br>**Acetyl-l carnitine (AlC)** is not recommended to prevent CIPN due to a possibility of harm.<br><br>There is insufficient evidence that **O3**, **FA**, **VE**, **EA** help to reduce neuropathy<br>________________________<br>Overall: There is a lack of strong evidence supporting the use of DM supplements (**DS**) or botanical agents (**Phy**) as supportive care and/or to manage breast cancer treatment-related side effects.
Other supportive interventions	Moore et al. 2002 [85]<br><br>Southampton, UK	RCT	European Organization for Research and Treatment of Cancer (EORTC), patients' satisfaction,	Adult lung cancer patients (n=203)	IG (n = 99): **nurse-led follow-up (NLF)** (Monday Friday), clinic and telephone assessment, communication with GP and primary care team, regular discussion with medical team.	Nurse specialists in lung cancer	The high degree of psychological morbidity among cancer patients suggests a need for close monitoring	IG had less severe dyspnea at 3 months and had better scores for emotional functioning and less peripheral neuropathy at 12 months.

[1] The number in brackets refer to the comprehensive reference list (see full article).
[2] A list of all abbreviations can be found at the end of Table S2.

Supplementary S4.

Treatments	Study[1]/ Country (cultural context)	Study design	Outcome measures (Patient-reported and others)	Patients (gender, tumor entity)	Intervention group (IG)/ control group (CG)	Who did conduct the intervention?	Concept	Results/Conclusion[2]
			measurement of symptom free survival, progression free survival, use of resources, general practitioners' satisfaction, and comparison of costs		CG (n = 103): Conventional care: routine outpatient appointments. (One post-treatment appointment, then appointments at two- or three-month intervals)		and support. Doctors and nurses often fail to detect patients' emotional distress and patients report that appointments are so "high speed" that they have little time to raise concerns.	IG scored significantly better in most satisfaction subscales at 3, 6, and 12 (for all subscales at 3 months)<br>__________<br>Overall: **NLF** was acceptable to lung cancer patients and general practitioners and led to positive outcomes.
Other TCM therapies	Wayne et al. 2018 [62]<br><br>Boston, Massachusetts, USA	Systematic review and meta-analysis (n=22 qualitative + 15 RCT)	Fatigue, sleep difficulty, depression, pain, and quality of life.	Patients with cancer-related symptoms	Clinical trials in with primary interventions **Tai Chi** (**TC)** and Qi Gong (**QG)**	Not described	**TC** and **QG** share a common history, which integrates elements of **Traditional Chinese Medicine (TCM),** martial arts conditioning, and lifestyle philosophy. Both incorporate elements of slow, gentle movement, awareness and regulation of breathing, as well as intentional direction of thoughts, attention, imagery, and sensation.	Two additional non-controlled studies in breast cancer also reported positive effects of **TC** and **QG** on pain (Lee et al. 2010 Galantino et al, 2013).<br>__________<br>Overall: Hints of positive effects with **TC** and **QG** in reducing pain.
Other TCM therapies	van Vu et al. 2017 [61]<br><br>Hong Kong, China	Systematic Review (15 RCTs, 8 CCTs)	Patient-reported physical symptoms (fatigue, pain, dyspnea, and weakness, lack of energy, nausea, dry mouth, constipation, early satiety, vomiting, and anorexia) and/or psychological symptoms (depression, anxiety, and mood disturbance) and quality of life.	Various cancer patients	**Qi Gong (QG)** was compared to usual care, placebo or other interventions.	Not described	**QG** consists of a series of simple, repeated practices including body posture or/and movements, breathing practice, and **meditation (Me)** performed in synchrony.	22 studies were included. No significant effect of **QG** on pain. A significant effect was observed on neuropathy symptoms only.<br>__________<br>Overall: Hints for effectiveness to reduce physical and psychological symptoms.

[1] The number in brackets refer to the comprehensive reference list (see full article).
[2] A list of all abbreviations can be found at the end of Table S2.

Supplementary S4.

**Treatments**	**Study[1]/ Country (cultural context)**	**Study design**	**Outcome measures (Patient-reported and others)**	**Patients (gender, tumor entity)**	**Intervention group (IG)/ control group (CG)**	**Who did conduct the intervention?**	**Concept**	**Results/Conclusion[2]**
Mind-body therapies<br><br>Hydrotherapy<br><br>Acupuncture, acupressure (TCM)<br><br>Manipulatives therapies<br><br>TENS/Scrambler therapy	Hökkä et al. 2014 [100]<br><br>Oulu, Finland	Systematic review (9 RCTs, 2 Quasi-experimental studies including 1047 patients	Perceived pain control and experience, pain intensity and pain interference	Patients with advanced cancer	Physical modalities: **massage (M)**, **physical therapy (PT), Transcutaneous electrical nerve stimulation TENS, acupuncture (A), reflexology (R), and wrapped warm footbath (FB**)<br><br>Cognitive modalities: **relaxation (Rel)**, **distraction therapy (DT), imagery (I) exercises** (**E**)	**M:** registered nurses, therapists and physiotherapists<br><br>**TENS:** medical researcher<br><br>**A:** radiation oncologist and a certified medical acupuncturist<br><br>**R**: certified reflexologists and women who had no training in **R** but were trained in lay foot manipulation protocol	Cancer pain is a multidimensional phenomenon and often a combination of different pain mechanisms (neuropathic pain and nociceptive pain). **NPI** have potential to reduce cancer pain.	Eleven studies were included. **M** was examined most frequently. **A** variety of measurement instruments were used to assess pain across the studies. There was some evidence that **M** might reduce pain immediately after the intervention, but no sustained effects were reported. **TENS** was shown to have the potential to decrease cancer bone pain. **A** was shown to reduce pain immediately but the benefit was transient and not significant compared with the social attention group.<br><br>All NPI were quite well tolerated.<br>____<br>Overall: It was not possible to draw conclusions about the effects and safety of the **NPI** in reducing cancer pain, but there is a need for more rigorous trials.
Phytotherapy<br><br>Rhythmical embrocations	Ostermann et al. 2008 [86]<br><br>Herdecke, Germany	Prospective observational study	Mood Scale (Bf-S), Pain Perception Scale (sensory PPS, affective PPS)	Patients (n=100) with chronic low back pain, age between 20 and 70 years	**Rhythmical embrocations** (RE) with herbal medicinewith a lavender/peat extract (Solum Oil, Wala Heilmittel GmbH)<br><br>**RE** according of the back with solum oil. 3 times within 24 days. 30 minutes of therapy followed by 20 minutes of rest.	One trained and experienced study nurse	Not described	The mean score of Zerssens adjective mood scale Bf-S was reduced from 25.8 before T1 to 13.3 after T3<br><br>Patients' mean sensory pain perception score was reduced from 18.8 at T1 to 15.2 at T3.<br><br>Pain perception score decreased from 29.8 at T1 to 21.3 at T3.<br><br>Pain intensity (VAS) immediately after treatment was reduced consistently, but also showed a tendency to decrease from T1 to T3.<br><br>In 37 patients, pain intensity at T3 was reduced below the individual patient target estimated at T1.

[1] The number in brackets refer to the comprehensive reference list (see full article).
[2] A list of all abbreviations can be found at the end of Table S2.

Supplementary S4.

**Treatments**	**Study[1]/ Country (cultural context)**	**Study design**	**Outcome measures (Patient-reported and others)**	**Patients (gender, tumor entity)**	**Intervention group (IG)/ control group (CG)**	**Who did conduct the intervention?**	**Concept**	**Results/Conclusion[2]**
								Overall: **RE** with Solum Oil is a promising and sensible complementary method for the treatment of chronic low back pain. A central sedative effect due to the volatile oil of lavender may be assumed.
Phytotherapy<br><br>Manipulative therapies	Lee et al. 2015 [56]<br><br>Seoul, Republic of Korea (South Korea)	Meta-Analysis (English, Chinese, Korean) (Twelve RCTs, including 559 participants)	Cancer pain	Adults with cancer pain (all types of cancer)	IG: all types of **massage (M), foot reflexology (FR), aromatherapy-massage (AT-M),**<br><br>CG: conventional care or no **M**	Not reported	**M** defined as the therapeutic manipulation by use of hands or mechanical devices for maintaining the suppleness of the body.	9 RCTs, 3 Controlled Clinical Trials (CCTs) included.<br>In 9 high quality studies based on the PEDro scale, reduction in cancer pain after **M**.<br><br>**M** significantly reduced cancer pain compared with no **M** or conventional care, particularly for the short term.<br><br>**FR** appears to be the most effective type of **M**, by comparison with body-**M** and **AT-M**.<br><br>Significant effects for **AT-M** in 2 studies (SMD −1.26 [95% CI −1.83 to -0.69]; P < .0001). However, the overall results show that the pain relief is short-lived (two weeks).<br>___<br>Overall: **M** is effective for the relief of cancer pain, especially for surgery-related pain. Among the various types of **M, FR** appeared to be more effective than body or **AT-M**.
Reiki	S3 guideline complementary medicine in the treatment of oncology patients, Nov 2020 [63]<br><br>Agdal et al (2011) - Odense, Denmark;	Systematic Review (Agdal et al. 2011), Cross-over study (Tsang et al. 2007)	Visual Analog sSale (Edmonton Symptom Assessment System [ESAS]) each session of **Rei** or rest	Patients with cancer pain (variety of cancers) (2007, 2011)	IG: **Reiki** (**Rei)**<br><br>CG: without **Rei**	Not described	**Rei** is a form of energy healing originating in Japan, in which special touches are used by appropriately trained practitioners. Through the laying	IG showed significantly better scores compared to CG (2011).<br><br>The study found no significant differences between IG and CG (2007).<br>___<br>Overall: It cannot be ruled out that the effect is attributable to subject attention or expectations.

[1] The number in brackets refer to the comprehensive reference list (see full article).
[2] A list of all abbreviations can be found at the end of Table S2.

Supplementary S4.

Treatments	Study[1]/ Country (cultural context)	Study design	Outcome measures (Patient-reported and others)	Patients (gender, tumor entity)	Intervention group (IG)/ control group (CG)	Who did conduct the intervention?	Concept	Results/Conclusion[2]
	Tsang (2007) - Calgary, Canada						on of hands or the sending of “energy” from a distance, the powers of the **Rei** practitioner are to be transferred to the patient and serve to strengthen him.	Due to the open design of the study and the small number of participants, the validity of the results is limited (2007).<br><br>In addition, little information on blinding, few statistical parameters, and because of the lack of a placebo group, it cannot be ruled out that the effect is attributable to subject attention or expectation (2011).
Hyperthermia	S3 guideline complementary medicine in the treatment of oncology patients,<br>Nov 2020 [63]<br><br>Kim et al. (2015) - Gwangju, Korea	Case—control study	Numeric Rating Scale (NRS), measurement of opioid dose NRS.	Patients with cancer pain (lung cancer)	IG: **Hyperthermia (HyT)** treatment about 60 min and a temperature between 39-42°C was reached in the area of the tumor.<br><br>CG: Only conventional cancer therapy.	Not described	**HyT** does not usually have the function of killing the cancer cells, but of making the tumor more sensitive to chemotherapy and radiotherapy. The prerequisite is either local or generalized heating to at least 40-43°.	No significant difference in pain intensity was found between groups.<br><br>A significantly higher opioid need was found in the IG at T1.<br><br>There were higher and therefore worse scores in the IG group, but the difference between the groups was only significant at T1.<br>____________________<br>Overall: It must be critically noted that in the CG at baseline, only 47.7% of the originally made patients still participated in the study.
Rhythmical embrocations	Aghabati et al. 2008 [104]<br><br>Teheran, Iran	Randomized and three-groups experimental study	Visual Analogue Scale (VAS) of pain, Rhoten Fatigue Scale (RFS	Cancer patients undergoing chemotherapy (n=90).<br><br>Patients had a diagnosis of cancer; had a normal level of consciousness (Glassco Coma Scale, GCS=15); 15–65 years, and had resided in the unit for at least 5 days	IG (n=30): **therapeutic touch** (**TT**)<br><br>Placebo group (n=30): mimic **TT**<br><br>CG (n=30): usual care	Interventions were conducted by practitioners	Physical body is surrounded by an aura and is penetrated and kept alive by a universal energy called prana (meaning vital force) that flows through the body and is transformed by chakras or non-physical vortices. Energy imbalance results in illness, which can be intuitively assessed	**TT** (significant) was more effective in decreasing pain and fatigue of cancer patients undergoing chemotherapy than the usual care group, while the placebo group indicated a decreasing trend in pain and fatigue scores com-pared with the usual care group.<br>____________________<br>Overall: The findings support a growing body of evidence that non-pharmacological interventions such as **TT** are effective for decreasing pain and fatigue for cancer patients undergoing chemotherapy.

[1] The number in brackets refer to the comprehensive reference list (see full article).
[2] A list of all abbreviations can be found at the end of Table S2.

Supplementary S4.

Treatments	Study[1]/ Country (cultural context)	Study design	Outcome measures (Patient-reported and others)	Patients (gender, tumor entity)	Intervention group (IG)/ control group (CG)	Who did conduct the intervention?	Concept	Results/Conclusion[2]
							in a form of psychic diagnosis, and then treated hands-on.	
Rhythmical embrocations	S3 guideline complementary medicine in the treatment of oncology patients, Nov 2020 [63]<br><br>Frank et al. (2007) - Boston, USA; Tabatabaee et al. (2016) - Tehran, Iran; Smarel et al. (1998) - Wayne, USA	RCT (2007), Systematic Review (1998, 2016)	Visual Analogue Scale (VAS),<br><br>Examination of various endpoints affected by pain (general activity, mood, walking ability, relationship with others, sleep) over four weeks.	Patients with cancer pain<br><br>Breast cancer patients (2007)<br><br>Breast cancer patients (1998)<br><br>Men with different cancer diagnoses (2016)	**Therapeutic touch (TT)** of women before and shortly after a breast biopsy (2007)<br><br>**TT** before and after the operation followed by a conversation and another group twice a rest period again followed by a conversation (1998)<br><br>IG: **TT**<br>CG: no intervention<br>**Placebo group**: Placebo (2016)	Trained practitioner or a sham intervention mimicking (2007)	**TT** belongs to energy healing and describes a kind of laying on of hands by a therapist. The patient is treated with or without direct contact of the hands. It is about guiding and shaping the individual energy of the treated by the therapist working with their energy fields. The focus is on balancing the person's energy and stimulating the body to activate its own natural healing powers	No significant differences between IG and CG in terms of pain after biopsy.<br>Patients who reported pain before biopsy had less pain after biopsy.<br>Patients who reported no pain before biopsy reported more pain after biopsy (2007).<br>No significant differences between IG and CG (1988).<br><br>Pain, which affected the patients' general activity, improved significantly in the **TT**-group compared to the placebo and CG (2016).<br>____________________<br>Overall: Number of subjects in the study was too small to provide meaningful results, and it cannot be ruled out that patients saw through the assignment to groups.<br><br>Without the initial comparability, the results of the study cannot be interpreted unambiguously, as the IG might have had better values already at baseline (systematic review).
Phytotherapy<br><br>Mind-body-therapies<br><br>Manipulatives therapies<br><br>Rhythmical embrocations<br><br>Other supportive interventions	Bardia et al. 2006 [15]<br><br>Rochester, Minnesota, USA	Systematic Review (of RCTs) including 18 RCTs	Pain scales	Patients with cancer related pain<br><br>A total of 1,499 study participants with cancer (not further described)	Intervention:<br>**Homeopathy** (**Ho**), **acupuncture** (**A**), **reflexology** (**R**), **hypnosis** (**H**), **imagery** (**I**), **relaxation** (**Rel**), **support groups** (**SG**), **music therapy** (**MuT**), **dietary supplements** (**DS**), **herbal medicine** (**Phy**), **DS** that do not contain herbs, **massage** (**M**), **aromatherapy** (**AT**), **magnet/laser therapy** (**MLT**), **healing touch** (**HeTo**), **Reiki** (**Rei**)	Not described in detail. In one of the trials evaluating **M** therapy, senior nursing students delivered the 10-minute intervention after a 1-hour training that included all	CAM have been used both as an alternative to conventional medicine (alternative medicine) and complementary to conventional medicine (complementary medicine). It has been suggested	18 trials were included. Seven trials reported significant benefit for: **A**, **SG**, **H**, **Rel**, **I**, **Phy** (oral)<br><br>Seven studies reported immediate postintervention or short-term benefit for: **A**, **MuT**, **Phy**,(oral) **M**, **HeTo.**<br>Four studies reported no benefit for **MuT**, **M**.<br>**AT-M**: One study added lavender **AT** to **M** and found no difference in effect on pain.

[1] The number in brackets refer to the comprehensive reference list (see full article).
[2] A list of all abbreviations can be found at the end of Table S2.

Supplementary S4.

Treatments	Study[1]/ Country (cultural context)	Study design	Outcome measures (Patient-reported and others)	Patients (gender, tumor entity)	Intervention group (IG)/ control group (CG)	Who did conduct the intervention?	Concept	Results/Conclusion[2]
						elements of study design and implementation. Other studies neglected to include any qualifications at all on the interventionists.	that they should be used in conjunction with conventional therapies in an integrative fashion (integrative medicine) and integrated with oncology clinics.	Overall: **H, I, SG, A**, and **HT** seem promising, particularly in the short term, but none can be recommended because of a paucity of rigorous trials. Future research should focus on methodologically strong RCTs to determine potential efficacy of these CAM interventions.
TENS/Scrambler therapy	Coyne et al. 2013 [67]<br><br>Richmond, Virginia, USA	Expanded trial, Single arm trial	Numeric rating scale (NRS), Brief Pain Inventory and European Organization for Treatment and Cancer QLC-CIPN-20 over time	16 men and 23 women<br><br>The cause of pain was CIPN in 33, post-mastectomy pain in 3, post herpetic neuropathy in 2, and radiation related pain in 1 person.	Each **Scrambler therapy (ST)** patient was given a 45-minute daily treatment for 10 consecutive days. The stimulus was increased to the maximum intensity individually bearable by the patient that did not cause any additional pain or discomfort.<br><br>No CG	Not described	**ST** is a novel approach to pain control that attempts to relieve pain by providing "non-pain" information via cutaneous nerves to block the effect of pain information. **ST** has relieved refractory chronic pain.	The "now" pain scores reduced from 6.6 before treatment to 4.5 at 14 days, 4.6, 4.8 and 4.6 at 1, 2 and 3 months.<br><br>Clinically important and statistically significant improvements were seen in average, least, and worst pain.<br><br>Overall: **ST** appeared to relieve cancer associated chronic neuropathic pain both acutely and chronically, and provided sustained improvements in many indicators of quality of life.
TENS/Scrambler therapy	Gewandter et al. 2019 [65]<br><br>Rochester, New York, USA	Single-arm study	The European Organization for Research and Treatment of Cancer-CIPN20 (EORTCCIPN20),Utah Early Neuropathy Score (UENS) and a forced choice monofilament test,<br><br>Interviews	The majority of the enrolled participants were female (58%) and Caucasian (90%). Many participants (n= 29) had tried gabapentin (57%), duloxetine (31%), or opioids (24%) previously to treat their CIPN symptoms.<br><br>The most common classes of neurotoxic chemotherapy agents were platinum agents	**TENS** intervention:<br>The device emits a 60–100-Hz stimulation with a 200–400-μs pulse duration and uses a biphasic waveform with an alternating leading phase.	Patients themselves, it was a wireless, home-based **TENS**	**TENS** is a safe, non-pharmacologic, and inexpensive therapy commonly used for pain control. In animal models, TENS reduces the enhanced central excitability of nociceptive neurons induced by inflammation and nerve injury and decreases release of the excitatory neurotransmitter glutamate in the	Significant improvements were observed with the EORTC-CIPN20 (percent change from baseline:(13%), SF-MPQ-2 (52%), numeric rating scale of pain (38%), tingling (30%), numbness (20%), and cramping (53%), and UENS large fiber sensation sub score (48%).<br><br>77% of the participants reported that they preferred to use the device on the continuous setting throughout the day more than using it for one session in the morning and one session in the evening.<br><br>Overall: It is feasible to evaluate **TENS** for CIPN using a wireless,

[1] The number in brackets refer to the comprehensive reference list (see full article).
[2] A list of all abbreviations can be found at the end of Table S2.

Supplementary S4.

**Treatments**	**Study[1]/ Country (cultural context)**	**Study design**	**Outcome measures (Patient-reported and others)**	**Patients (gender, tumor entity)**	**Intervention group (IG)/ control group (CG)**	**Who did conduct the intervention?**	**Concept**	**Results/Conclusion[2]**
				(45%) and taxanes (31%)			dorsal horn of the spinal cord.	home-based device and that further evaluation of **TENS** for CIPN in a randomized clinical trial is warranted.
TENS/Scrambler therapy	Loprinzi et al. 2020 [71]<br><br>Rochester, New York, USA	RCT, randomized Phase II pilot trial. Two arms	EORTC CIPN20, three-item 0–10, NAS questionnaire regarding CIPN associated pain, tingling, and numbness, second questionnaire that addressed the amount of CIPN pain, CIPN numbness and CIPN tingling.	Adult cancer patients (n = 50) with chronic CIPN symptoms for at least 3 months and CIPN-related tingling or pain at least 4/10. Chronic CIPN of at least moderate severity.	IG (n = 25): **ST** 30 min, given for 10 consecutive weekdays in Mayo Clinic.<br><br>CG (n = 25): **transcutaneous electrical nerve stimulation (TENS)** 30 min per day, for 14 days at home.	**ST**: not reported.<br>**TENS**: patients themselves after education by study nurse and written instruction.	Electrical stimulation to replace endogenous pain information with synthetic “non-pain” or “normal-self” electrical signals. These signals are hypothesized to travel through the peripheral afferent somatosensory C fibers to the brain, innervating the region of pain.	Twice as many **ST**-treated patients who had at least a 50% documented improvement during the 2 treatment weeks, from their baseline pain, tingling, and numbness scores, when compared with the **TENS**-treated patients.<br><br>Global impression of Change scores for “neuropathy symptoms,” pain, and quality of life were similarly improved during the treatment weeks.<br><br>IG were more likely than those in the CG to recommend their treatment to other patients, during both the 2-week treatment period and the 8-week follow-up period. Minimal toxicity was observed.<br>____________________________<br>Overall: Patients suffering from CIPN, **ST** improves problematic symptoms more than does therapy with a **TENS** unit.

[1] The number in brackets refer to the comprehensive reference list (see full article).
[2] A list of all abbreviations can be found at the end of Table S2.

Supplementary S4.

List of abbreviations

Acetyl-l-carnitine	AIC	External applications	EAP	Massage	M	Reflexology	R
Acupressure	AP	Fatty acids	FA	Meditation	Me	Rehabilitation intervention	RI
Acupuncture	A	Fine motor training	FMT	Menthol	Men	Reiki	Rei
Aerobic	Ae	Foot massage	FM	Mind-body modalities	MBM	Relaxation	Rel
Alpha-lipoic acid	ALA	Frozen gloves	FG	Mind-body therapies	MBT	Resistance training	RT
Aromatherapy	AT	Footbath	FB	Movement therapies	MT	Rhythmical embrocations	RE
Balance training	BT	Foot reflexology	FR	Music therapy	MuT	Scrambler therapy	ST
Cardiovascular exercise	CardE	Glutamine	G	Natural products	NP	Self-management strategies	SMT
Chinese medicine	CH	Goshajinkigan	Go	Nerve conduction studies	NCS	Sensorimotor training	SM
Closed kinematic chain exercises	CKC-E	Hand and foot baths	HFB	Non-pharmacological interventions	NPI	Sports therapy	SpT
Cognitive behavioural intervention	CBI	Healing touch	HeTo	Nurse-led follow-up	NLF	Support groups	SG
Complementary and Alternative Medicine	CAM	Heat application	He	Nutritional therapy	NT	Strength training	STr
Complementary and Integrative Medicine	CIM	Henna application	HA	Occupational therapy	OT	Tactile stimulation	TS
Complementary treatments	CTs	Homeopathy	Ho	Omega-3	O3	Tai Chi	TC
Compression	Com	Humor therapy	HT	Pain management strategies	PMS	Therapeutic touch	TT
Coordination training	CoT	Hydroelectric bath	Heb	Passive mobilisation	PM	Topical C. colocynthis	TCC
Cryocompression	CC	Hydrotherapy (Kneipp)	HTK	Patient education	PE	Topical therapy	ToT
Cryotherapy	CT	Hyperthermia	HyT	Physical activity	PhA	Traditional Chinese Medicine	TCM
Cupping	C	Hypnosis	H	Physical therapy	PT	Transcutaneous electrical nerve stimulation	TENS
Cycling	Cy	Hypothermia	Hypo	Physiotherapy	PhyT	Vitamin B12	VB
Dietary modification	DM	Imagery	I	Phytotherapy (incl. herbal medicines, botanical agents)	Phy	Vitamin B complex	VBC
Dietary supplements	DS	Integrative therapies	ITs	Positioning	Po	Vitamin E	VE
Distraction therapy/training	DT	Kneipp therapy	KT	Problem-solving therapy	PST	Walking	W
Electroacupuncture	EA	Magnesium replacement	MR	Progressive muscle relaxation	PMR	Whole-body vibration	WBV
Electrotherapy	ET	Magnet/Laser therapy	MLT	Psychosocial approaches	PA	Yoga	Y
Exercise (all types)	E	Manual therapy	MaT	Qi Gong	QG		

[1] The number in brackets refer to the comprehensive reference list (see full article).
[2] A list of all abbreviations can be found at the end of Table S2.
