# Supplementary material for: Prevention and Treatment of Chemotherapy-Induced Peripheral Neuropathy (CIPN) with Non-Pharmacological Interventions: Clinical Recommendations from a Systematic Scoping Review and an Expert Consensus Process
Source: Med Sci (Basel). 2023 Jan 30;11(1):15. doi: 10.3390/medsci11010015 (PMC9944490; doi:10.3390/medsci11010015)
Supplement: Supplementary file 1 [file medsci-11-00015-s001.zip › Supplementary S6, Table S4.pdf]

Supplementary S6.

Table S4. Expert judgements of nursing procedures for treating CIPN based on consented rounds of consensus finding

Treatments	Interventions	Safety	Clinical experience		Effort of training	Practical feasibility
			Prophylactic application	Therapeutic application		
		**S**	**CE** (x of the 6 institutions voted x/6)		**ET**	**PF**
Phytotherapy	Aconit oil application	S	1/6 apply it, E cannot be assessed	E3 5/6	ET1	PF
Phytotherapy + Rhythmical embrocation	Aconit oil – rhythmical embrocation (e.g., hand and foot)	S	/	E 4 5/6	ET4	PFt
Phytotherapy	Solum oil application	S	/	E1 1/6	ET1	PF
Movement therapy (Sensorimotor training)	Sugar oil peeling (1 tablespoon olive oil and 1 dessertspoon sugar)	S	/	E3 1/6	ET1	PF
Hydrotherapy + Phytotherapy	Alkaline bath for hand/foot, then Aconit oil application	S	/	E3 1/6	ET1	PF
Phytotherapy	Flaxseed bath (3 tablespoons flaxseed with 2,5 l water, 5 min cooking)	S	1/6, E cannot be assessed	E4 1/6	ET1	PF
Phytotherapy	Arnica comp/Formica oil application	S	1/6, E cannot be assessed	E3 1/6	ET1	PF
Phytotherapy + Rhythmical embrocation	Arnica comp/Formica oil – rhythmical embrocation	S	/	E4 1/6	ET4	PFt
Phytotherapy	Arnica comp/Formica ointment (for stronger effect of Aconit)	S	/	E3-4 1/6	ET1	PF
Phytotherapy	Rosemary ointment	S	/	E3-4 1/6	ET1	PF
Movement therapy (Sensorimotor training)	Tactile stimulation (special naturopathic procedures: beeswax kneading, brush massage, electric toothbrush, hedgehog ball massage, needle stimulating mat, rape bath, quartz sand bath)	S	4/6, E cannot be assessed	E2-3 6/6	ET1	PF
Cryotherapy	frozen gloves and socks	S	4/6, E cannot be assessed	/	ET2	PF
Compression	medical gloves one size smaller than usual size	S	2/6, E cannot be assessed	/	ET1	PF
Other care intervention	Copper ointment (0.4%) for cold feet and feelings of numbness	S	/	E2 1/6	ET1	PF
Phytotherapy	Peppermint oil application for heat sensations and paresthesia	S	/	E2 1/6	ET1	PF
Phytotherapy	Eucalyptus oil application for heat sensations and paresthesia	S	/	E2 1/6	ET1	PF
Hydrotherapy	Cold knee and/or arm showers	S	/	E3 1/6	ET1	PF

Abbreviations: **S** = Safe; **CE** = Clinical experience (rated on a numerical scale 0 to 5 with 0 = no effect and 5 = maximum effect); **ET** = Effort of training (Education requirements in addition to a nursing grade; 0 = no additional instructions or education needed; 1 = instructions needed, 2 = application under guidance, 3 = repeated practice needed, 4 = basic training of Rhythmical embrocation (200 hours) recommended, but partial skills can be acquired with less than 200 hours, 5 = basic training of Rhythmical embrocation (200 hours) needed; **PF** = Practical Feasibility (PFt = feasibility limited due to time requirements; PFtt = feasibility strongly limited due to time requirements; PFc = feasibility limited due to high costs (> 30 € per month)
